# Radiocleavable rare-earth nanoactivators targeting over-expressed folate receptors induce mitochondrial dysfunction and remodel immune suppressive microenvironment in pancreatic cancer

**DOI:** 10.1186/s12951-025-03657-8

**Published:** 2025-08-12

**Authors:** Tanvi Gupta, Shang-Rung Wu, Li-Chan Chang, Forn-Chia Lin, Yan-Shen Shan, Chen-Sheng Yeh, Wen-Pin Su

**Affiliations:** 1 Institute of Clinical Medicine, College of Medicine, National Cheng Kung University, No. 35, Rd. Xiaodong, Tainan, 704 Taiwan; 2 School of Dentistry & Institute of Oral Medicine, College of Medicine, National Cheng Kung University, Tainan City, 701 Taiwan; 3 Department of Radiation Oncology, College of Medicine, National Cheng Kung University Hospital, National Cheng Kung University, Tainan, 704 Taiwan; 4 Department of Surgery, College of Medicine, National Cheng Kung University Hospital, National Cheng Kung University, Tainan, 704 Taiwan; 5 Department of Chemistry, National Cheng Kung University, Tainan, 701 Taiwan; 6 Center of Applied Nanomedicine, National Cheng Kung University, Tainan, 704 Taiwan; 7 Department of Medicinal and Applied Chemistry, Kaohsiung Medical University, Kaohsiung, 807 Taiwan; 8 Departments of Oncology and Internal Medicine, National Cheng Kung University Hospital, College of Medicine, National Cheng Kung University, Tainan, 704 Taiwan; 9 Clinical Medicine Research Center, National Cheng Kung University Hospital, College of Medicine, National Cheng Kung University, Tainan, 704 Taiwan

**Keywords:** Nano therapy, Folic acid, Folate receptor, Ferroptosis, Immunogenic cell death

## Abstract

Pancreatic cancer is a fatal cancer with poor prognosis and survival rate, often diagnosed usually in the advanced stage of disease. The conventional methods are usually considered for surgery or chemotherapy, and neo-adjuvant therapies have improved the survival rate in the patients. Folic acid plays a crucial role in the synthesis, metabolism, and repair of DNA; thereby, it is considered one of the biomolecules for cancer-targeted therapy for highly expressed receptors to overcome poor vasculature and dense tumor stroma, as in pancreatic cancer. This study strategizes for improving the therapeutic efficacy of pancreatic cancer via folate receptor-guided nanoparticles. The conjugation of folic acid (FA) to the LiYF_4_:Ce^3+^nanoparticles (SCNP-FA) with the photocleavage chemical molecule; firstly enters the cells through receptor-mediated endocytosis and then, releases FA intracellularly upon the trigger of radiation in a controlled manner. This nano-based approach induces ferroptosis to provoke immunogenic cell death (ICD) with higher generation of reactive oxygen species (ROS) and accumulation of lipid peroxides. It shows an abundant damage to the mitochondria and a decrease in mitochondrial membrane potential (MMP) upon treatment. This targeted therapy remodels the immunosuppressive tumor microenvironment and releases damage-associated molecular patterns (DAMPs) to initiate an immune response. These findings reveal the anti-tumor response with folate receptor-guided nanoparticles in pancreatic cancer.

**Graphical abstract:**

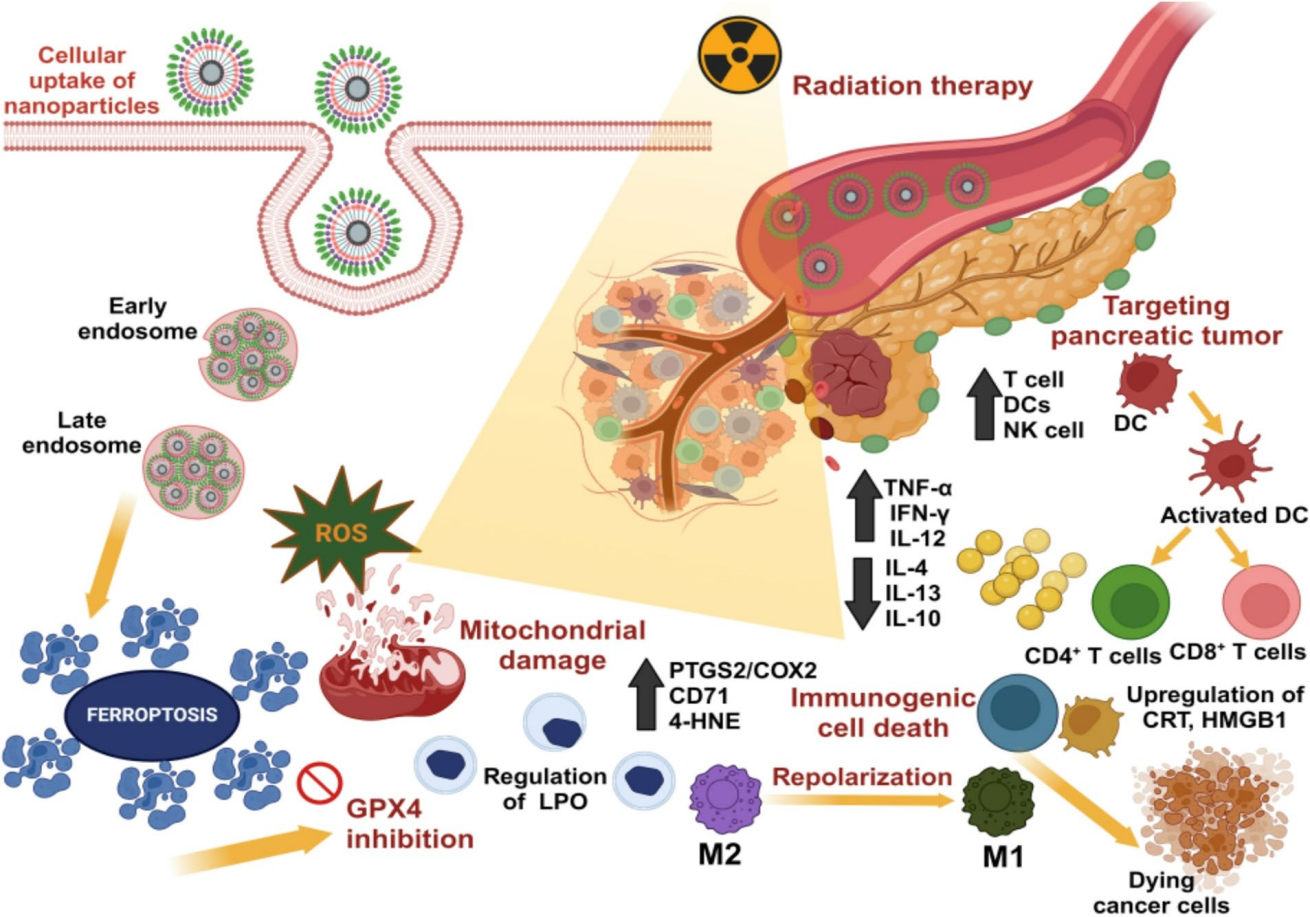

**Supplementary Information:**

The online version contains supplementary material available at 10.1186/s12951-025-03657-8.

## Introduction

Pancreatic cancer is one of the deadliest among solid tumors and is expected to become the 2nd leading cause of death due to cancer in the coming years [1]. The incidence rate has risen by 0.5-1% per year, and the 5-year survival rate has increased to 12% with improvements in systemic therapy. The most preferred treatment is surgical resection, but about 10–15% of patients have localized or resectable tumors [2, 3]. While the standard care of treatment involves combining surgery with chemotherapy or radiotherapy, which has little impact and often comes with multiple side effects [4]. Due to its aggressive nature, the cancer grows rapidly and usually reaches the advanced stage of disease due to poor prognosis. The current treatment methods fail to cure due to high vasculature and dense stroma underlying the tumor microenvironment, which results in poor therapeutic effect and chemoresistance [5]. More developments need to be addressed with targeted therapies and immunomodulation of the tumor microenvironment to improve the therapeutic efficacy [6]. 

Nano-based therapy can potentially target cancer cells residing inside tumors specifically and enhance effectiveness. It results in strong interaction between the cells and immune cells, producing cell-mediated immune responses, thereby suppressing tumor growth [7]. Metal nanoparticles are synthesized with the core of metals, which possess high surface area and volume ratio, with characteristic features for clinical use. Due to their low cytotoxicity, mono-disperse nature, size-dependent surface reactivity, and interaction with specific receptors/ligands, through which they can enter inside the cells and organelles as well as interact with proteins, enzymes, or DNA, makes them more suitable for the modern medicine. They can destroy tumor cells by generating reactive oxygen species, cellular changes, and inhibition of proliferation. Also, they promote the enhanced drug delivery system for the targeted delivery of drugs to reach the cancer cells, reducing the toxic side effects of drugs and enhancing therapeutic efficacy [8]. Due to its vast vascular system and poor lymphatic clearance, the metal nanoparticles are helpful in the targeted therapy of the tumor tissue via the enhanced permeability and retention (EPR) effect. Many studies have confirmed that metal nanoparticles loaded with drugs having pH-responsive, temperature, electrical, and light-sensitive properties release drugs abundantly under the influence of physical and chemical conditions to enhance the anti-tumor effect [9, 10]. 

Folate or folic acid is the natural form of vitamin B9 and plays an important role in various biological processes. It plays a crucial role in nucleotide synthesis via the methylation of uracil to thymine, which is involved in DNA repair and synthesis [11]. Folate is associated with cancer prevention in various cancer types such as oral cancer [12] esophageal adenocarcinoma, squamous cell carcinoma [13] renal cell cancer [14] leukemia [15] skin cancer [16] colon [17] breast cancer [18] and ovarian cancer [19]. The folate expression levels have been higher in many human cancer types, such as lung, colorectal, ovarian, breast, brain, and endometrial cancer, compared to healthy tissues [20–22]. Little has been explored on pancreatic cancer as folate receptor targeting could improve the anti-tumor efficacy. With the use of nanomaterials, the efficacy and potency of folic acid can be enhanced by targeting the folate receptors on the tumor tissue.

Regulated cell death (RCD), also known as programmed cell death, is regulated by bio-macromolecules. Ferroptosis is one of the RCD types, where the alterations in the intracellular or extracellular microenvironment lead to the activation of signal transduction and, ultimately, lead to cancer cell death. It also plays a crucial role in the immune response. It usually shows morphological and bioenergetic characteristics, such as the shrinkage or rupture of mitochondria, a decrease in mitochondrial cristae, the generation of reactive oxygen species (ROS), the inactivation of the glutathione peroxidase 4 (GPX4) enzyme, and an increase in lipid peroxidation products. At present, the compounds that induce ferroptosis are effective in treating only some cancer types, as others show sensitivity towards ferroptosis. So, through nanotechnology loading, the ferroptosis inducer can potentially target the tumor site with the maximum drug localized at the specific site, thereby improving the anti-tumor effect via ferroptosis [23]. It has been found to enhance the immune responses by induction of immunogenic cell death (ICD) [24]. The dying cancer cells release damage-associated molecular patterns (DAMPs), such as high mobility group box 1 (HMGB1) and calreticulin (CRT), into the tumor microenvironment, promoting dendritic cell maturation and recruiting immune cells for the amplification of ICD [25]. Also, the nanoparticles that can induce ferroptosis act as immunostimulants, which leads to the maturation of dendritic cells (DCs), which further recruit T cells to enter the tumor microenvironment and kill more cancer cells. They can also repolarize (pro-tumorigenic) M2 macrophages, which help in tumor progression into (tumoricidal) M1 macrophages, inhibiting tumor progression [26]. The other cells that help in the immunotherapeutic response upon treatment of cancer cells are CD8 + T cells, DCs, NK, MDSCs, Tregs, and TAMs, which help in anti-tumor immunity and tumor suppression [27]. Despite the whole mechanism still being elusive for treating pancreatic cancer, which has not been explored in this context.

In this study, we demonstrated that the radio-cleavable rare-earth metallic nanoparticles conjugated with folic acid release the controlled drug from the nanocarrier upon the trigger of the radiation effect. It induces ferroptosis majorly and activates the ICD in the pancreatic cancer cells. It also leads to the morphological changes in the cell organelles, causing mitochondrial dysfunction and an increase in ROS and lipid peroxidation. With the advantage of nanotherapy, the drug delivers to the targeted site of the tumor and internalizes into the cell via receptor-mediated endocytosis for its enhanced anti-tumor response. Folic acid has not been studied for pancreatic cancer, and the conventional treatments opted for have poor survival rates and side effects due to toxic drugs. This nano-based approach significantly improves the immune-based therapeutic effect in pancreatic cancer.

## Results and discussion

### Characterization of SCNPs and FA release from the nanocarrier

**Fig. 1:**
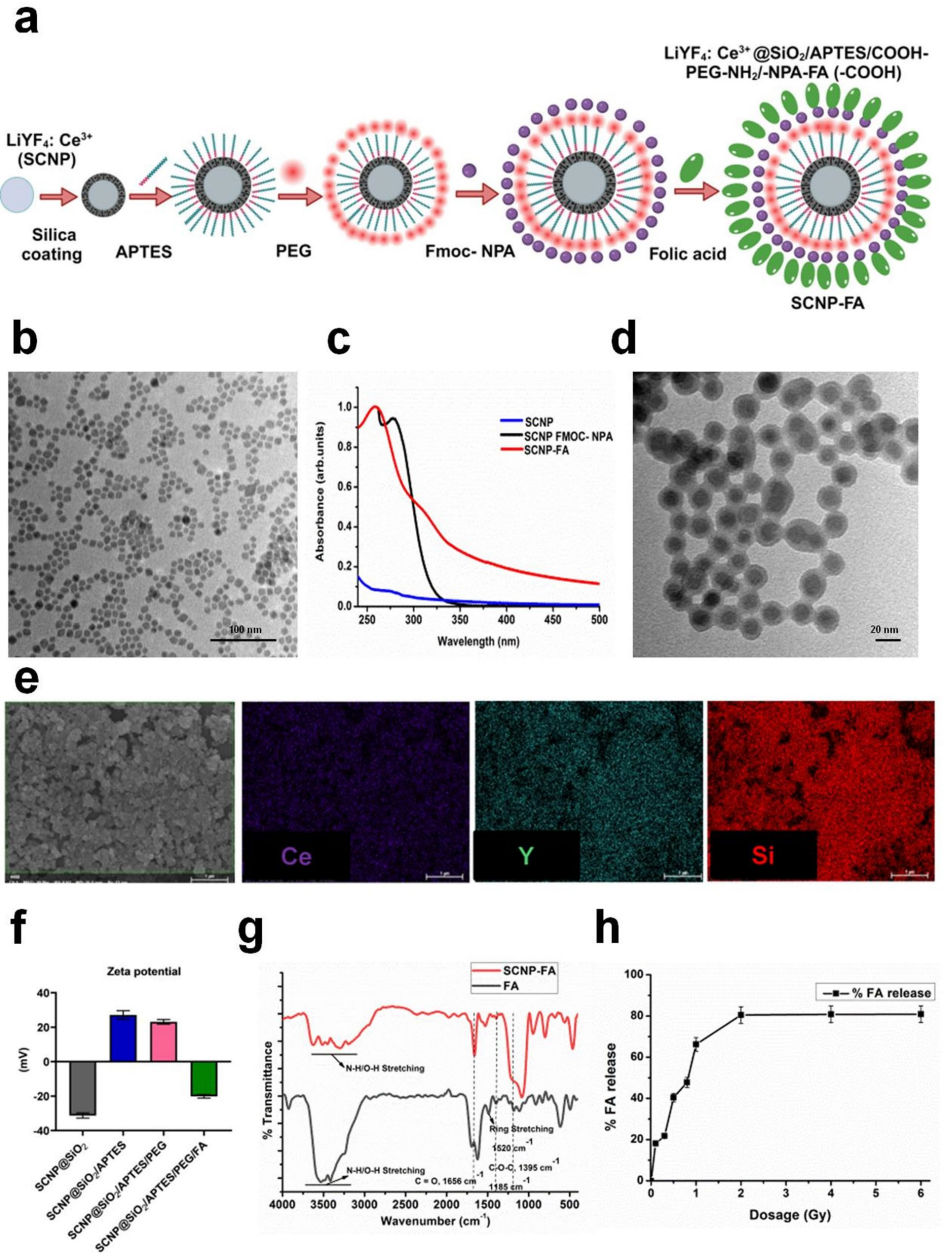
Design of scintillating nanoparticles (SCNPs) to release folic acid and its characterization. (**a**) Illustration of synthesis of LiYF_4_:Ce^3+^ NPs conjugated to folic acid (FA). (**b**) TEM image of LiYF_4_:Ce^3+^ NPs. (**c**) UV absorbance spectrum confirming the modification of SCNPs with Fmoc-NPA and conjugated with FA. (**d**) TEM image of SCNP-FA. (**e**) Elemental mapping of synthesized SCNP-FA. (**f**) Zeta potential after silica, APTES, PEG modification, and conjugation with FA (*n* = 3). (**g**) FTIR spectrum of free drug FA and SCNP-FA (**h**) X-ray dose-dependent release of FA upon photo-cleavage process

The synthesized LiYF_4_: Ce^3+^ SCNPs were prepared by thermal decomposition, as shown in the scheme illustration of the surface modification of the nanoparticles, similar to our previous publication [28]. In this study, we have conjugated the drug Folic Acid (FA) (Fig. 1a**)**. The SCNPs were depicted through a transmission electron microscope (TEM), with an average size of 13–15 nm **(**Fig. 1b**)**. The absorbance spectrum of SCNPs with the Fmoc-NPA is important as it is responsible for the photo-cleavage, and this NPA molecule is light-sensitive and could be cleaved upon irradiation. The folic acid drug was conjugated to NPA with the EDC-NHS chemistry to obtain SCNP@SiO_2_/PEG/NPA-FA. The absorbance spectrum of FA at 256 nm absorbance peak confirmed the successful conjugation of the drug **(**Fig. 1c**)**. The size of SCNP@SiO_2_/PEG/NPA-FA was determined through TEM, about 43 ± 2 nm **(**Fig. 1d**)**. The elemental mapping of SCNP@SiO_2_/PEG/NPA-FA was also performed to confirm the presence of different elements through HR-TEM **(**Fig. 1e and Figure S1a**)**. The morphology of the SCNPs appears to be hexagonal, as determined by the selected electron diffraction (SAED) area, as shown in (Figure S1b-c). The HR-TEM shows the crystalline nature of the nanoparticles with the interplanar spacing of 0.417 and 0.245 nm in SCNPs and 0.498 nm in SCNP@SiO_2_/PEG/NPA-FA (Figure S2a-b). The surface charge of SCNP@SiO_2_ was determined to be −31.25 mV and was changed to + 27.15 mV upon the modification with APTES due to the presence of an amine group. The SCNP@SiO_2_/APTES was conjugated with COOH-PEG-NH_2_ by EDC-NHS chemistry, showed + 23.12 mV surface charge due to the presence of amine group on its surface and finally after the conjugation of FA, the surface charge of SCNP@SiO_2_/PEG/NPA-FA was determined to be −20.12 mV due to the presence of –COOH group **(**Fig. 1f**)**. The nanoparticles, free FA drug, and surface functional groups were determined through Fourier transform infrared (FTIR) analysis **(**Fig. 1g**)**. The absorption standard curve was plotted through the linear fitting of absorbance intensity against the known concentration of FA to determine the quantification of FA conjugated to nanoparticles (Figure S3a-b). With this standard curve, the FA was quantified from the initial concentration of FA in the conjugated reaction and the unreacted FA concentration in the reaction. It was determined that 53.75 nM of FA can be conjugated to 1 ppm NPs (Figure S3c). The Raman spectroscopy was also performed to determine the vibrational patterns and peaks upon the conjugation of FA to SCNPs (Figure S4). This nanoparticle involves the synthesis of rare-earth metals, which have unique properties involved in the cancer treatment for tumor inhibition. Silica coating of the nanoparticles is usually used to improve biocompatibility, enhance efficacy, and reduce toxicity. Conjugation with PEG provides enhanced biodistribution and longer circulation period, with the amine group covalently attaching targeted ligands or drugs. The fluorenylmethoxycarbonyl-2-nitro-phenylalanine (Fmoc-NPA), a photolabile linker, which is further de-protected by secondary amine, piperidine, for the nanoparticles to become light-sensitive, and the folic acid is conjugated to the NPA molecule, which can be cleaved upon radiation. This nanoparticle is unique because it can allow controlled drug release only upon radiation. It can achieve high efficiency at low radiation dose due to its nanoparticle and radiotherapy sensitization along with active targeting at the overexpressed folate receptors on the cancer cells. It releases the drug at the specific tumor site, avoiding any off-target effects on the healthy organs, which can provide a targeted response. Folate serves as a tumor-targeting ligand due to the selective overexpression of folate receptors on the cancer cells and has minimal expression on the normal tissues and has enhanced receptor-mediated internalization, which, thereby, allows enhanced drug delivery at the specific tumor site and avoids side effects in the healthy organs. This nano-based strategy aims for the controlled release of the drug upon irradiation to avoid any toxic effects, which shows the dose-dependent release of FA upon the different dosages of X-rays from 0.1 to 6 Gy, which showed the maximum FA release of 80% from 2 to 6 Gy **(**Fig. 1h**)**. The stability of nanoparticles was determined before proceeding with the in vitro or in vivo studies, where SCNP@SiO_2_/PEG/NPA-FA was dispersed in a PBS, culture medium with pH 5.5 and pH 7.5 to monitor changes in an acidic or basic environment. This was observed for about 7 days, incubated at 37 °C under dark conditions. After the indicated time points, the solution was taken out to determine whether FA was released in the solution. It was analyzed through UV absorbance, which showed no peak, and through TEM to determine its morphology, which showed no apparent changes, and no precipitation was observed (Figure S5).

### Expression level of folate receptors and cellular uptake in vitro

**Fig. 2:**
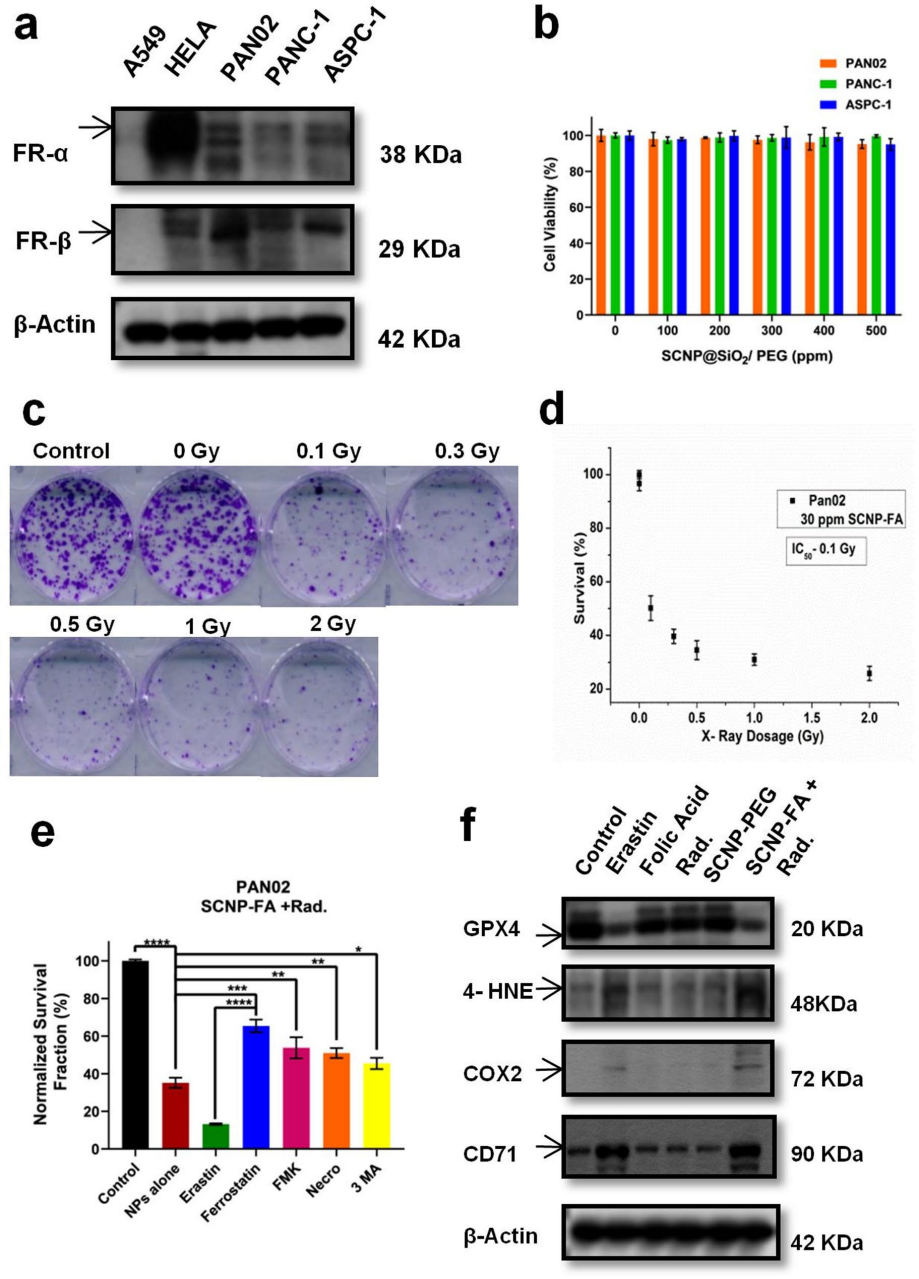
NPs targeting over-expressed folate receptors induce cell death by promoting ferroptosis. (**a**) Protein expression levels of folate receptors α and β in A549 (negative control), HeLa (positive control), PAN02, PANC-1, and ASPC-1 (*n* = 3). (**b**) Cytotoxicity of SCNP-PEG confirmed by MTT assay in PAN02, PANC-1, and ASPC-1 (*n* = 3). (**c**) Colony assay of PAN02 treated with SCNP-FA induced radiation. (**d**) Survival percentage of PAN02 with IC_50_ of 0.1 Gy (*n* = 3). (**e**) Different cell death inhibitors in PAN02 treated with SCNP-FA induced radiation (*n* = 3). (**f**) Protein expression levels of different markers of ferroptosis, such as GPX4, 4-HNE, COX2, and CD71 in PAN02 after various treatments. Erastin acts as a positive control (*n* = 3). Statistical significance was determined by Student’s t-test. Data are presented as mean ± SD (*P < 0.05, **P < 0.01 *** *P* < 0.001, ****P < 0.0001)

Pancreatic cancer shows high expression levels of folate receptor present in two isoforms- folate receptor α (FOLR1) and folate receptor β (FOLR2), which involves targeting the receptor with a drug that can easily be taken up in the cancer cell lines for its anti-tumor effect. It has already been known that Hela cells are folate receptor-positive, and A549 cells are folate receptor-negative [29, 30]. Here, we determined the protein expression levels of both FR-α and FR-β in the human and murine pancreatic cell lines. FR-β showed a strong expression level in all pancreatic cell lines compared to FR-α, but the PAN02 cell line showed a higher expression level than the other human cell lines **(**Fig. 2a, FigureS27). To uncover the cellular uptake of the SCNP-FA in all the cell lines, an additional group was added to compare the excess amount of FA with the SCNP-FA. It was observed that the pancreatic cell lines showed higher cellular uptake with SCNP-FA, and the HeLa cells with positive folate expression. Still, the additional presence of FA leads to a decline in cellular uptake, indicating that the ability to target folate receptors with excess FA decreases (Figure S6). Therefore, these results suggest that nanoparticles show a targeted response to the over-expressed folate receptor cell lines.

### Cytotoxicity study in vitro and NPs kill tumor cells via ferroptosis

**Fig. 3:**
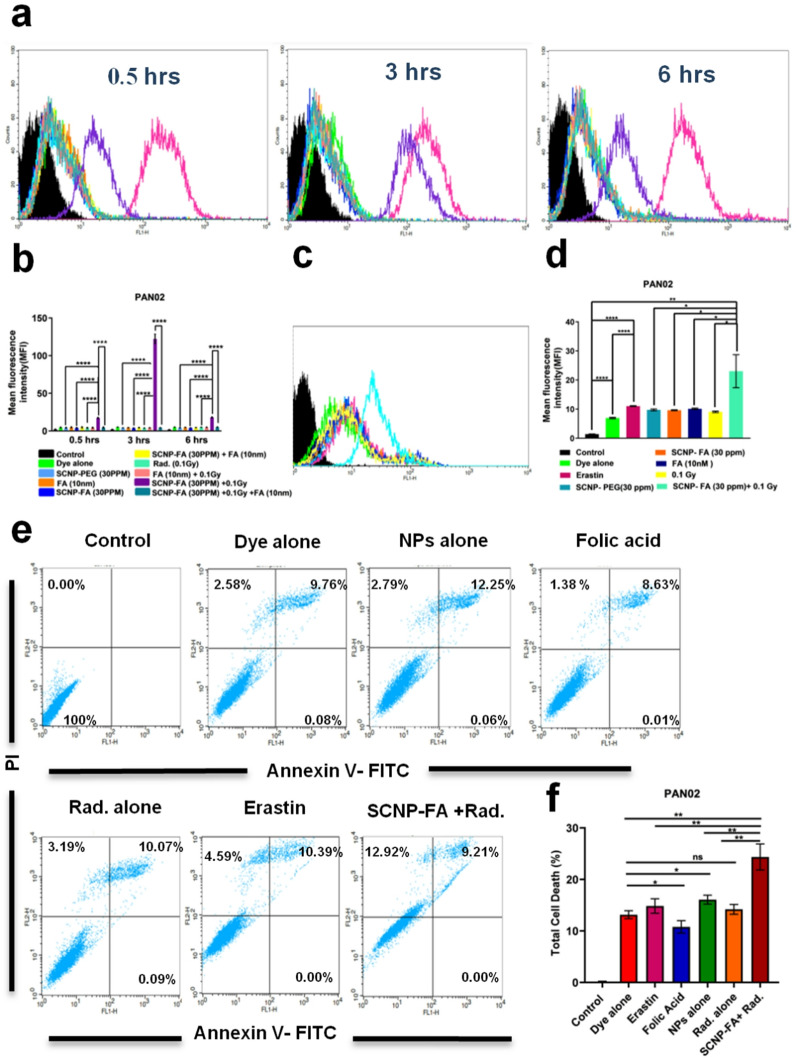
Determining ROS production and cell death via ferroptosis. (**a**) Intracellular ROS detected in a time-dependent manner by H2DCFDA in different treated groups in PAN02, with their respective histogram peak depending on their fluorescence intensity. The black curve represents the untreated control group, green curve represents the H2DCFDA dye alone group, light blue curve represents the SCNP-PEG alone treated group, orange curve represents the FA alone treated group, dark blue curve represents the SCNP-FA alone treated group, yellow curve represents the SCNP-FA with excess FA treated group, turquoise curve represents radiation alone treated group, pink curve represents FA with radiation treated group, purple curve represents SCNP-FA with radiation treated group, dark green curve represents SCNP-FA with excess FA and radiation treated group and dark pink curve represents the positive H_2_O_2_ control treated group. The significant rightward shift in fluorescence intensity indicative of elevated ROS generation.(**b**) Mean fluorescence intensity by flow cytometric analysis (*n* = 3). (**c**) Lipid ROS detected by C11-BODIPY in different treated groups in PAN02, with their respective histogram peak depending on their fluorescence intensity. The black curve represents the untreated control group, green curve represents the C11-BODIDY dye alone group, pink curve represents the positive erastin control treated group, light blue curve represents the SCNP-PEG alone treated group, orange curve represents the SCNP-FA alone treated group, dark blue curve represents the FA alone treated group, yellow represents the radiation alone treated group and turquoise curve represents the SCNP-FA with radiation treated group. The significant rightward shift in fluorescence intensity indicative of elevated lipid ROS generation.(**d**) Mean fluorescence intensity by flow cytometric analysis (*n* = 3). (**e**) Flow cytometric analysis of determining cell death by Annexin V-FITC/PI staining in different treated groups with erastin as a positive control. (**f**) Total cell death percentage in PAN02 upon treatment (*n* = 3). Statistical significance was determined by ordinary two-way ANOVA and Student’s t-test. Data are presented as mean ± SD (*P < 0.05, **P < 0.01)

We confirmed the cytotoxicity of SCNP@SiO_2_/PEG to evaluate the biocompatibility of the nanoparticles in the pancreatic cell lines. It was checked in the murine-PAN02 and human-PANC-1 and ASPC-1 cell lines, and no changes in the cell viability were found with the different concentrations of nanoparticles **(**Fig. 2b**)**. A colony assay was performed to determine the inhibitory dosage of SCNP-FA caused by X-ray radiation (Rad.), and it revealed IC_50_ for PAN02 was 0.1 Gy with 30 ppm of SCNP-FA **(**Fig. 2c-d**)**. It was observed that this treatment was very effective in killing cancer cells with a minimal dose of X-ray. The colony cell survival was also determined in PANC-1 and ASPC-1 in a dose-dependent manner for X-ray (Figure S7a-d). To further explore the mechanism of SCNP-FA + Rad. - induced cell death, cell death inhibitors such as ferrostatin-1, Z-VAD-FMK, necrostatin-1, and 3-methyladenine were used as these compounds are responsible for inhibiting ferroptosis, apoptosis, necrosis, and autophagy, respectively [31]. Erastin was used as a positive control as it is responsible for inducing ferroptosis, which ultimately led to cell death via nanoparticles, while upon treatment with ferrostatin-1, the cells in PAN02 were rescued, and it was also confirmed in PANC-1 and ASPC-1 cells (Figure S8a-b). It was also observed that other inhibitors could also modulate the cell death, indicating a mixed type of cell death caused by these nanoparticles, but about 65% of cells were rescued due to ferrostatin-1 compared to other inhibitors, which was about 50% (Fig. 2e). Therefore, the protein expression level of GPX4 was analyzed, which is associated with ferroptosis [32] showed decreased expression upon the treatment with SCNP-FA + Rad., indicating inactivation of glutathione peroxidase 4. There are certain parameters that are involved in this study that influence cell death. Folate can be sufficient to disrupt the intracellular redox homeostasis, especially in the case of targeted delivery via receptor-mediated endocytosis. The nanoparticle platform amplifies the ROS production due to the presence of high-Z elements and radiosensitizing nanoparticles. The folate uptake in the cancer cells influences the endosomal and lysosomal conditions, with the presence of intracellular esterases, which accelerate the release from the nanoparticles. Importantly, out of all the cell lines, PAN02 showed higher expression of the folate receptors **(**Fig. 2a**)**, which allows preferential uptake of folic acid from the nanoparticles for greater therapeutic payload effect, enhancing efficacy at even lower release fractions. So, it was confirmed that each cell line will have a different dosage for its inhibitory effect. And, we further moved our experiments with their respective dosage for each cell line and found similar results with different dosages. Lipid peroxidation is usually affected by unsaturated fatty acids residing inside the cell membrane. The lipid peroxidation products include lipid hydroperoxides and reactive aldehydes such as malondialdehyde (MDA) and 4-hydroxynonenal (4-HNE), the regulators for ferroptosis [33]. Herein, the expression level of 4-HNE increased in SCNP-FA + Rad. compared to the other alone treatment groups. Over-expression of certain genes/proteins is considered a biomarker for ferroptosis, such as prostaglandin-endoperoxide synthase 2/cyclooxygenase-2 (PTGS2/COX2), an enzyme responsible for prostaglandin biosynthesis [34]. The expression level of COX2 enhanced in SCNP-FA + Rad. compared to the other alone treatment groups. The transferrin receptor (CD71) plays a crucial role in driving ferroptosis through its interaction via transferrin [35]. The protein expression of CD71 was also up-regulated in SCNP-FA + Rad. in all pancreatic cell lines **(**Fig. 2f, Figure S8c-d, Figure S27, 28). Due to lipid peroxidation, polyunsaturated fatty acids (PUFAs) are oxidized by reactive oxygen species (ROS) to produce lipid hydroperoxides, which indicates that ferroptosis is ROS-dependent [36]. In order to confirm ferroptosis occurred due to SCNP-FA + Rad., cytosolic and lipid ROS were detected by 2´,7´-dichlorodihydrofluorescein diacetate (H2DCFDA) and 4,4-difluoro-5-(4-phenyl-1,3-butadienyl)−4-bora-3a,4a-diaza-s-indacene-3-undecanoic acid (BODIPY^®^ 581/591 C11) fluorescent probes respectively. The time-dependent treatment was followed after the radiation exposure of 0.5, 3, and 6 h to confirm the cytosolic ROS through flow cytometry, where the PBS, SCNP-PEG, FA, Rad., and SCNP-FA showed no changes in the ROS level. It was observed that SCNP-FA + Rad. increased ROS level at 0.5 h resulted in the highest level of ROS at 3 h and decreased at 6 h. It indicates that the production of ROS starts to increase upon the release of the FA drug from the nanocarrier upon the radiation treatment and reaches the maximum level at 3 h (Fig. 3a, b). The same results were observed in PANC-1 and ASPC-1 cells, as shown in Figure S9a-b. We also performed an additional experiment with SCNP-PEG at increasing concentrations and different time points, such as 24 and 48 h, subjected to radiation, which showed no significant ROS generation, determined through H2DCFDA assay. This finding supports the biological inert nature of the SCNP-PEG under irradiation and confirms that the therapeutic effect occurs specifically upon the FA release and not from the carrier itself (Figure S29a-d). The lipid ROS was also detected upon radiation treatment, showing an increased level of SCNP-FA + Rad. compared to the alone treatment groups (Fig. 3c, d). Similar results were observed in PANC-1 and ASPC-1, as shown in Figure S10a-b. The nanoparticles generate ROS in cells under X-ray irradiation due to their ability to absorb and convert X-ray energy into secondary electrons. Then, these electrons will interact with water and oxygen molecules in the local environment, producing a variety of ROS such as hydroxyl radicals and hydrogen peroxide. This nanoparticle contains high-Z elements, which amplify energy deposition via the photoelectric effect. The FA is only released from the nanoparticle upon radiation trigger in the cancer cells with overexpressed folate receptors present on their surface. Annexin V-FITC/PI staining through flow cytometry [37] was performed to determine cell death. SCNP-FA + Rad treatment led to a significant increase of Annexin-V- positive and PI- positive cells, indicating dual ferroptotic/apoptotic cell death compared to the other alone treatment groups, which had minimal effect on the cell death **(**Fig. 3e, f**)**. This was also confirmed in PANC-1 and ASPC-1, as shown in Figure S11a-b. Minimal early apoptotic population suggests that cells bypass early apoptotic signaling, undergoing rapid lipid peroxidation-driven membrane disruption, consistent with ferroptotic-like morphology. Together, it indicates that nanoparticles release FA upon the radiation treatment, leading to cell death due to ferroptosis.

### Cellular function led to dysfunction upon treatment with nanoparticles in cancer cells

**Fig. 4:**
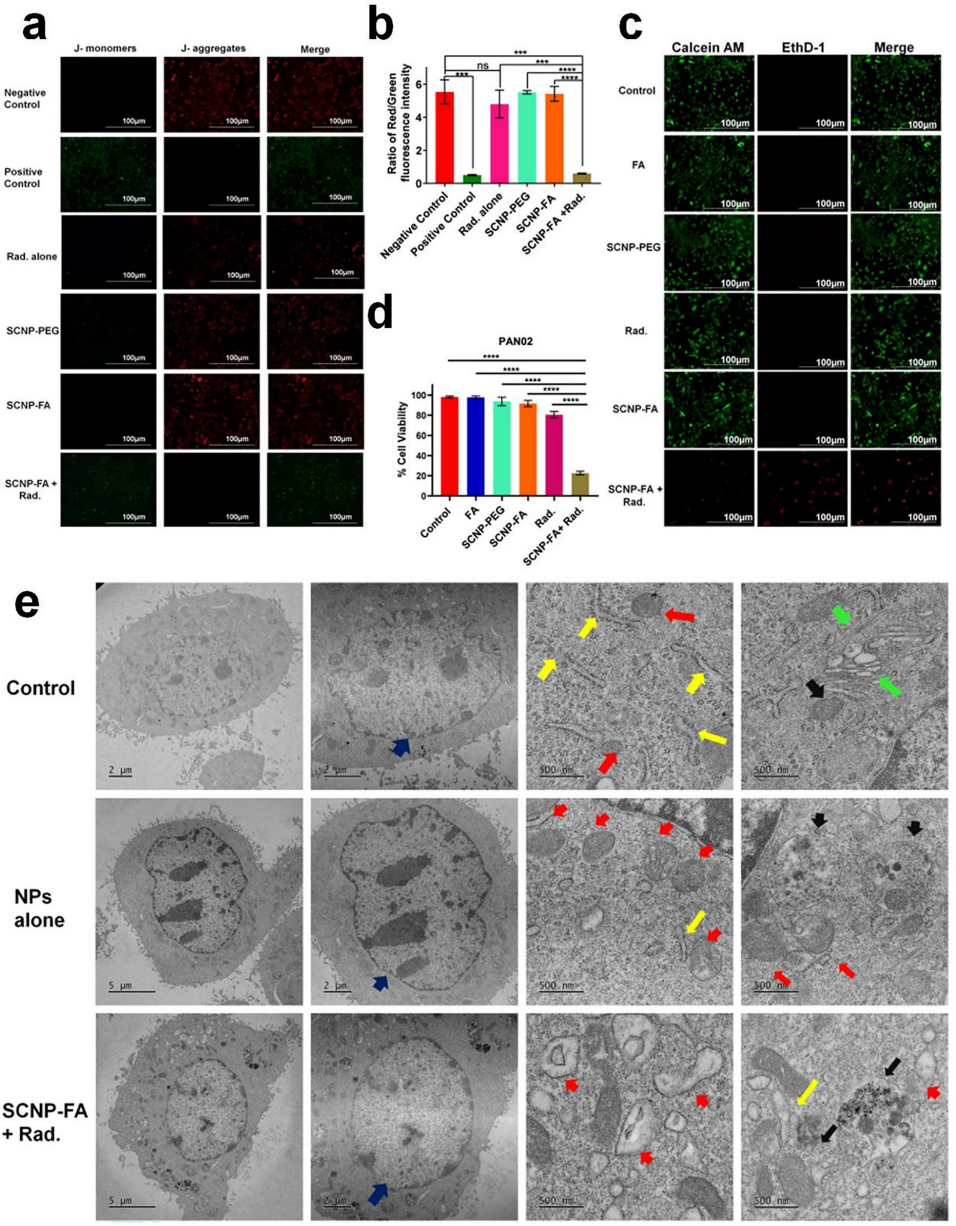
SCNP-FA-induced radiation treatment promotes cellular loss. (**a**) Determining mitochondrial membrane potential (MMP, ΔΨm) by JC-1 assay upon treatment with different groups with CCCP as a positive control. (**b**) The ratio of red/green fluorescence intensity signals (*n* = 3). (**c**) Live/dead staining by Calcein AM and EthD-1 in various treated groups. (**d**) Cell viability percentage in PAN02 (*n* = 3). The scale bar stands for 100 μm. (**e**) TEM image of the PAN02 cells treated with PBS (24 h), NPs alone (24 h), and SCNP-FA induced radiation (24 h). Different arrowheads marked in the image show various organelles residing inside the cell. Blue- shows the nucleus, Red- shows the mitochondria, Yellow- shows the endoplasmic reticulum, Green- shows the Golgi apparatus, and Black- shows the endosome, where different stages of the endosome can be observed upon the treatment. Three different cells were examined in each treatment group. Statistical significance was determined by Student’s t-test. Data are presented as mean ± SD (**P < 0.01 *** *P* < 0.001, ****P < 0.0001)

Mitochondrial membrane potential (MMP, ΔΨm) is generated by complexes I, III, and IV, which are essential for energy storage after oxidative phosphorylation, and therefore, it helps in the regulation of ferroptosis [38]. We investigated the role of MMP by JC-1 assay in pancreatic cell lines to understand the effect on membrane polarization. Usually, if the mitochondria are healthy, they will have higher MMP, forming JC-1 aggregates (red). However, they will possess lower MMP if unhealthy or dying, forming JC-1 monomers (green). It was observed that after the nanoparticle treatment alone, it didn’t affect the MMP; also, the radiation alone showed a minimal decrease in MMP, but, comparatively, it led to a decrease in MMP upon the nanoparticle-induced radiation treatment (Fig. 4a, b). This result was also observed in PANC-1 and ASPC-1 cells, as shown in Figure S12a-b. Next, co-staining with calcein AM (for live cells) and Ethidium homodimer-1 (EthD-1 for dead cells) was performed to determine the number of live and dead cells in various treatment groups. It was observed that radiation alone showed minimal effect compared to the other treatments, which had no effect, whereas the highest amount of dead cells was observed in SCNP-FA + Rad. treatment group **(**Fig. 4c, d**)**. We also confirmed the live and dead cells in the PANC-1 and ASPC-1 cells, as shown in Figure S13a-b. One of the morphological features of ferroptosis is designated with the “ballooning phenotype,” which is referred to as loss of membrane integrity, cytoskeleton rearrangement, swelling and ballooning of organelles, especially mitochondria and endoplasmic reticulum due to lipid peroxidation [39, 40]. To check the morphology of cancer cells upon treatment with SCNP-FA + Rad., it was observed there was a ballooning phenotype similar to the positive control, erastin, whereas the other groups showed no obvious behaviour in all the three pancreatic cell lines (Figure S14a-c). In the case of ferroptosis, the cells usually exhibit necrotic changes in morphology, showing swelling in the cytoplasm and loss of membrane integrity. Usually, the mitochondrial abnormalities are most prominent with loss of cristae and membrane, while the nuclear changes are not dominant. Compared to necrosis, ferroptosis is associated with multiple mechanisms and ultimately leads to programmed cell death [41, 42]. Next, to examine the internalization of nanoparticles and determine the ultra-structural features of PAN02 cells upon treatment with SCNP-FA + Rad., the morphology of the cell organelles was investigated through TEM analysis. It was found that significant cellular uptake of nanoparticles takes place through cell-mediated endocytosis upon treatment within 24 h. The nanoparticles were seen inside the endo-lysosomal membranes and were uniformly distributed upon the treatment with nanoparticles alone, compared to the nanoparticle-induced radiation group, which showed the aggregation of nanoparticles inside the endosome, indicating the digestion upon killing of cancer cells. The mitochondria led to the loss of cristae and membrane integrity as well as shrunken in size, swelling, and rupture of the outer membrane were observed, indicating the ability of cell targeting by nanocarrier, which led to the controlled release of FA upon radiation through FA receptor binding. Also, the endoplasmic reticulum showed swelling upon the nanoparticle-induced radiation compared to nanoparticles alone or the control group **(**Fig. 4e**)**. These results indicate that the cellular function of cancer cells was compromised upon treatment with nanoparticle-induced radiation.

### Mitochondrial dynamics are affected due to nanoparticles and induce ICD markers

**Fig. 5:**
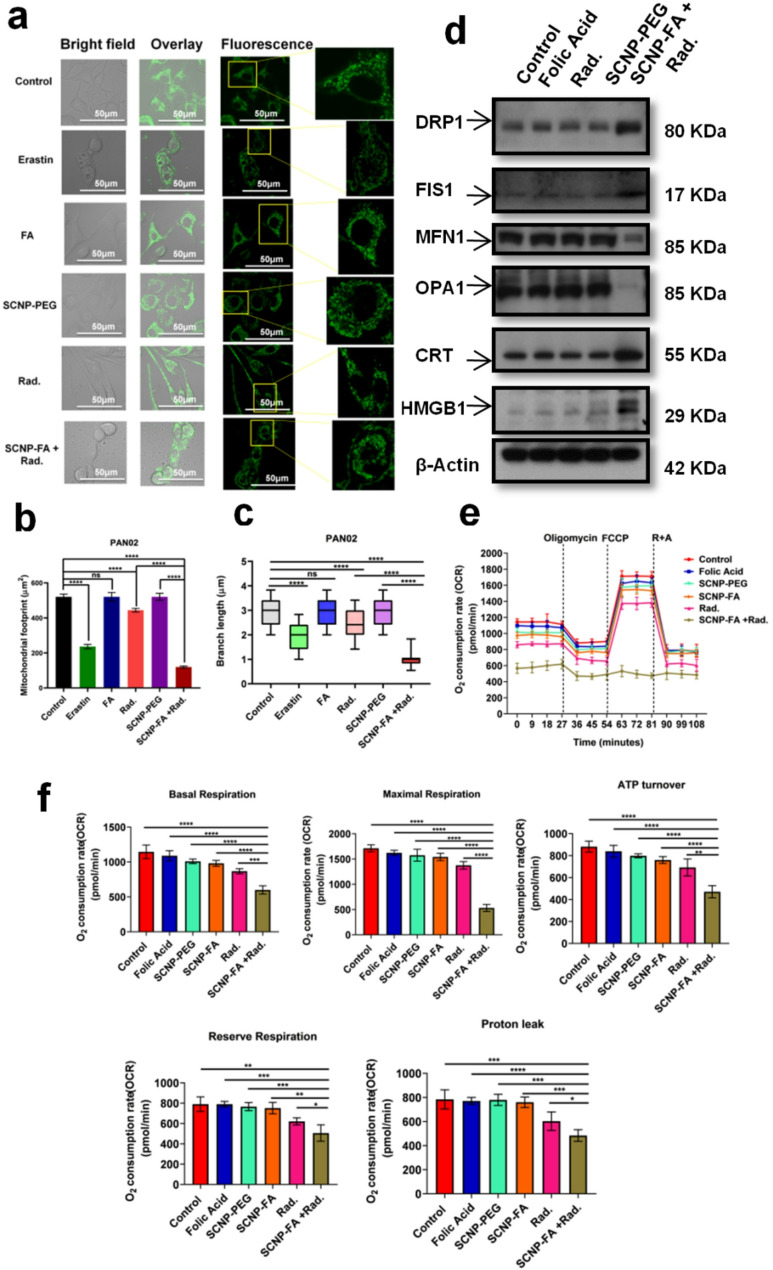
SCNP-FA-induced radiation treatment promotes mitochondrial dynamics to induce ICD. (**a**) Mitochondrial morphology examined by mitotracker green in PAN02 cells treated with various groups and erastin as a positive control. The scale bar stands for 50 μm. (**b**) mitochondrial footprint. (**c**) branch length. Three different areas were examined in each treatment group. (**d**) Protein expression levels for fission, fusion, and ICD markers were analyzed upon treatment with various groups (*n* = 3). (**e**) Seahorse analysis to measure oxygen consumption rate (OCR) upon treatment with various groups. (**f**) Basal respiration, maximal respiration, ATP turnover, reserve respiration, and proton leak were measured. Statistical significance was determined by Student’s t-test. Data are presented as mean ± SD (*P < 0.05, **P < 0.01 *** *P* < 0.001, ****P < 0.0001)

**Fig. 6:**
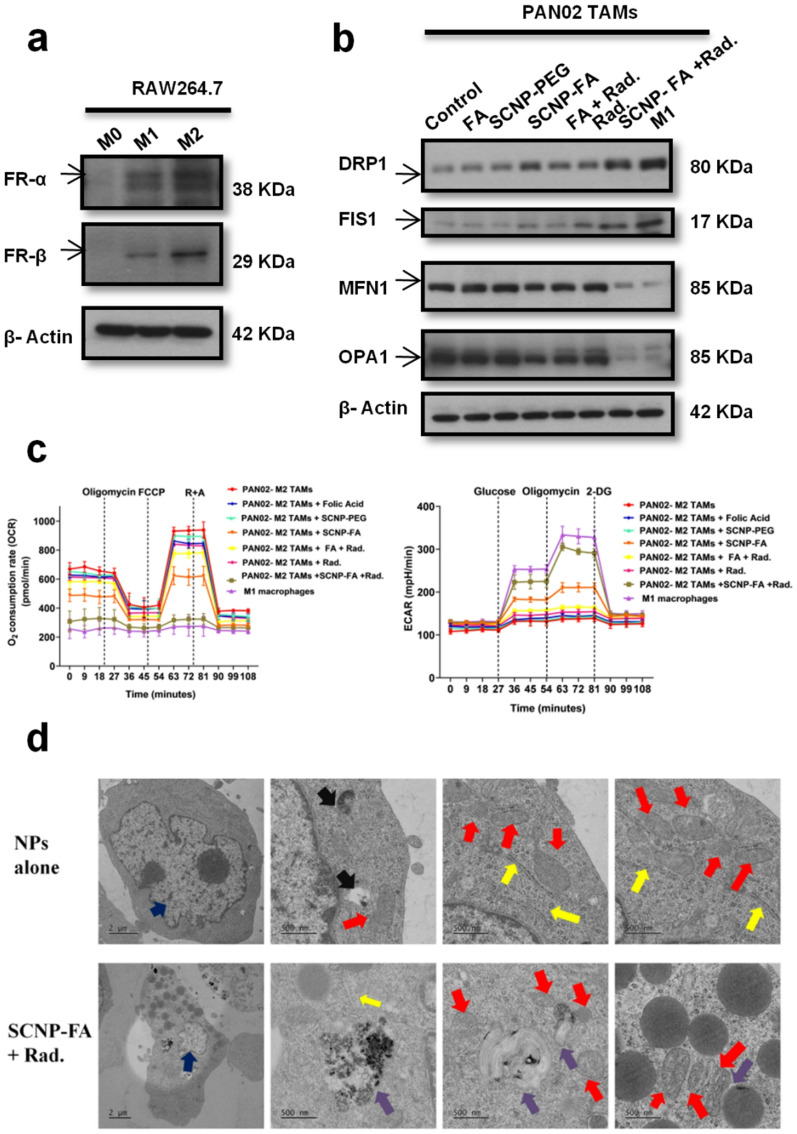
SCNP-FA-induced radiation treatment on tumor-associated macrophages (TAMs) alters mitochondrial dynamics. (**a**) Protein expression levels of folate receptors α and β in M0, M1, M2 type macrophages (*n* = 3). (**b**)Protein expression levels for fission and fusion were analyzed upon treatment with various groups (*n* = 3). (**c**) Seahorse analysis to measure oxygen consumption rate (OCR) and extracellular acidification rate (ECAR) upon treatment with various groups. (**d**) TEM image of the RAW264.7 cells treated with NPs alone (24 h) and SCNP-FA induced radiation (24 h). Different arrowheads marked in the image show various organelles residing inside the cell. Blue- shows the nucleus, Red- shows the mitochondria, Yellow- shows the endoplasmic reticulum, Purple- shows the nanoparticle internalization in the lysosome, and Black- shows the endosome. Three different cells were examined in each treatment group

Mitochondria are dynamic organelles that undergo fission and fusion to maintain membrane integrity and cellular homeostasis. Fusion occurs when the outer membrane and inner membrane of the two mitochondria join with the involvement of mitofusin proteins such as mitofusin 1 (MFN1), mitofusin 2 (MFN2), and optic atrophy protein 1 (OPA1) [27]. Fission occurs when the mitochondria divide into two organelles due to dynamin-related protein 1 (Drp-1). FIS1 (Mitochondrial Fission Protein 1) plays an important role in mitochondrial dynamics, especially in mitochondrial fission, contributing to multiple cellular processes such as cell fate, cell signaling, and energy production. Both fusion and fission are closely related to ferroptosis, interfering with mitochondrial dynamics [43]. The morphology and quantity of mitochondria were evaluated to verify the mitochondrial alterations upon the treatment of nanoparticle-induced radiation in pancreatic cell lines. The control group showed normal mitochondria with a structured tubular-like pattern as well as alone treatment groups indicated no obvious changes, whereas the SCNP-FA + Rad. group showed puncta structures with aggregated and swollen mitochondria, resulting in fragmentation and fission, which was significantly confirmed with the mitochondrial footprint and branch length **(**Fig. 5a-c**)**. The other two cell lines also revealed the same pattern of the mitochondria (Figure S15a-b). The protein expression levels of fission markers were confirmed in PAN02 cells, where DRP-1 and FIS1 showed increased expression upon SCNP-FA + Rad. treatment compared to the other groups. The fusion markers, MFN1 and OPA1, decreased expression upon SCNP-FA + Rad. treatment compared to the other groups **(**Fig. 5d, Figure S27**)**. The ASPC-1 and PANC-1 cells also confirmed the same results (Figure S16, S28). Immunogenic cell death (ICD) is cell death activated by the immune system to trigger immune responses [44], and is associated with the release of various damage-associated molecular patterns (DAMPs) such as calcium reticulum protein (CRT) and high mobility group protein 1 (HMGB1) [45]. Some research studies have stated ferroptosis as ICD and that it affects various immune cells in the tumor microenvironment (TME) [46–48]. To further evaluate whether SCNP-FA + Rad. exerts an effect on CRT and HMGB1, the protein expression levels of both the markers were checked and found they were highly expressed upon the nanoparticle-induced radiation treatment compared to other groups in all three pancreatic cancer cell lines (Fig. 5d). To evaluate the mitochondrial function as the oxidative phosphorylation complexes were altered, the Seahorse Mito-Stress test was used to determine the oxygen consumption rate (OCR) in PAN02, thereby detecting basal respiration, maximal respiration, ATP turnover, reserve respiration, and proton leak. Therefore, we found that the respiration decreased significantly upon the SCNP-FA + Rad. treatment compared to other groups, which showed only a minimal effect **(**Fig. 5e, f**)**. FA is not a classical ferroptosis inducer; our findings suggest that its controlled, receptor-mediated intracellular delivery via functionalized nanoparticles can indirectly contribute to ferroptotic cell death and subsequent ICD. The folate receptors are overexpressed on the pancreatic cancer cells, which allow efficient nanoparticle uptake and enhanced intracellular accumulation of FA. Once internalized, the localized release of FA may cause metabolic stress through the dysregulation of one-carbon metabolism and NADPH homeostasis, pathways critical for maintaining cellular redox balance. Excess FA has been reported to modulate redox-sensitive pathways and disrupt GPX4, a key regulator of ferroptosis [49, 50]. This sensitizes the cells to lipid peroxidation, leading to ferroptotic cell death, particularly involving X-ray irradiation, which can amplify ROS production. The controlled release of FA from the nanoparticles likely enhances the immunogenic signaling by creating an intracellular environment of oxidative stress and membrane destabilization. Therefore, our nanosystem not only leverages FA as a targeting ligand but also exploits its biological activity in redox modulation, allowing a dual-functional role in facilitating selective delivery and contributing to ferroptosis-induced ICD. To further confirm whether the macrophages express folate receptors for targeting, the protein expression levels revealed that both the M1 and M2 macrophages were positive for both folate receptors. However, M2 showed higher expression than M1 (Fig. 6a, FigureS27). Next, to understand the mitochondrial dynamics of M2-tumor associated macrophages (TAMs) co-cultured with RAW264.7 and PAN02 cells, it was observed that the protein expression levels of fission markers such as DRP1 and FIS1, were highly expressed than the fusion markers such as MFN1 and OPA1 in the nanoparticle induced radiation treatment similar to the M1 macrophage serves as a positive control, followed by low expression level in SCNP-FA alone (Fig. 6b, Figure S27). Furthermore, to evaluate how mitochondrial respiration and glycolysis were affected upon treatment, a Seahorse assay was performed for the M2-TAMs, which resulted in a decrease in OCR of basal respiration, maximal respiration, ATP turnover, reserve respiration, and proton leak in SCNP-FA + Rad. treatment and increase in extracellular acidification rate (ECAR) of glycolysis, glycolytic capacity, and glycolytic reserve in SCNP-FA + Rad. treatment compared to the other treatments, and a similar effect was observed in the M1 macrophage (Fig. 6c, S17**)**. Next, to examine the morphology of the macrophages upon treatment with nanoparticles and observe the internalization process was performed through TEM. As it has been confirmed above, M2 macrophages express folate receptors, which can easily allow the penetration of nanoparticles inside the endo-lysosomal membranes through cell-mediated endocytosis within 24 h. The nanoparticle alone did not affect the morphology of any organelles, whereas the nanoparticle-induced radiation treatment showed that the cell was shrunken, and the nucleus size was reduced. More of the nanoparticles have been digested inside the endo-lysosomes. It can be seen that more lysosomes are present, and some of the mitochondria have led to the loss of cristae, which confirms the cell targeting via folate receptors **(**Fig. 6d). The MMP of the M2-TAMs was also investigated, and various treatments were subjected to the JC-1 assay to understand the effect on membrane polarization. The SCNP-FA + Rad. treatment showed a lower MMP, similar to the M1 macrophage, while the other treatments showed an enhanced MMP (Figure S18). Finally, the mitochondrial alterations were also confirmed by mitotracker dye to observe the morphology and quantity of mitochondria subjected to various treatments. The control, SCNP-PEG, and FA groups showed normal mitochondria. In contrast, the SCNP-FA + Rad. group showed fragmented mitochondria with aggregated puncta structures similar to the M1 macrophage, confirmed by the decreased mitochondrial footprint and branch length (Figure S19a-c). Thus, mitochondrial disruption occurs upon the nanoparticle-induced radiation treatment and induces ICD, which may have a promising role in immune-based therapy.

### Nanoparticle-induced radiation effect exerted anti-tumor response in vivo

**Fig. 7:**
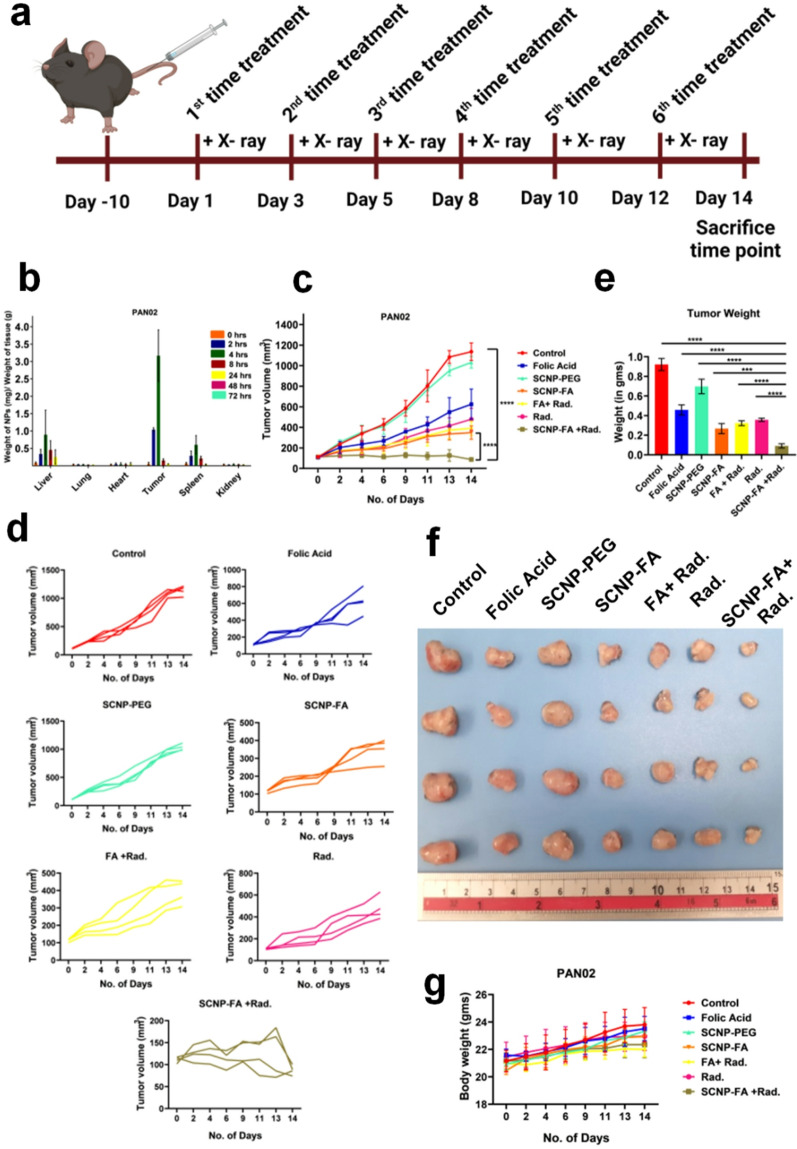
SCNP-FA-induced radiation treatment suppresses pancreatic cancer development. (**a**) Scheme of tumor inoculation and treatment administration. (**b**) PAN02 tumor-bearing mice were intravenously treated with SCNP-FA, and ICP-MS determined the bio-distribution of NPs for the Y^3+^ concentration at different time intervals (0, 2, 4, 8, 24, 48, and 72 h) (*n* = 3). (**c**) Tumor growth curve in PAN02 subjected to various treatments (*n* = 4). (**d**) individual tumor growth curves in control, FA alone, SCNP-PEG, SCNP-FA, FA + Rad., Rad. and SCNP-FA + Rad. groups monitored for 2 weeks (*n* = 4). (**e**) tumor weight (*n* = 4). (**f**) Visualized image of tumors after the sacrifice of mice upon the treatment. (**g**) body weight measured upon completion of treatment (*n* = 4). Statistical significance was determined by ordinary two-way Anova and Student’s t-test. Data are presented as mean ± SD (*** *P* < 0.001, ****P < 0.0001)

**Fig. 8:**
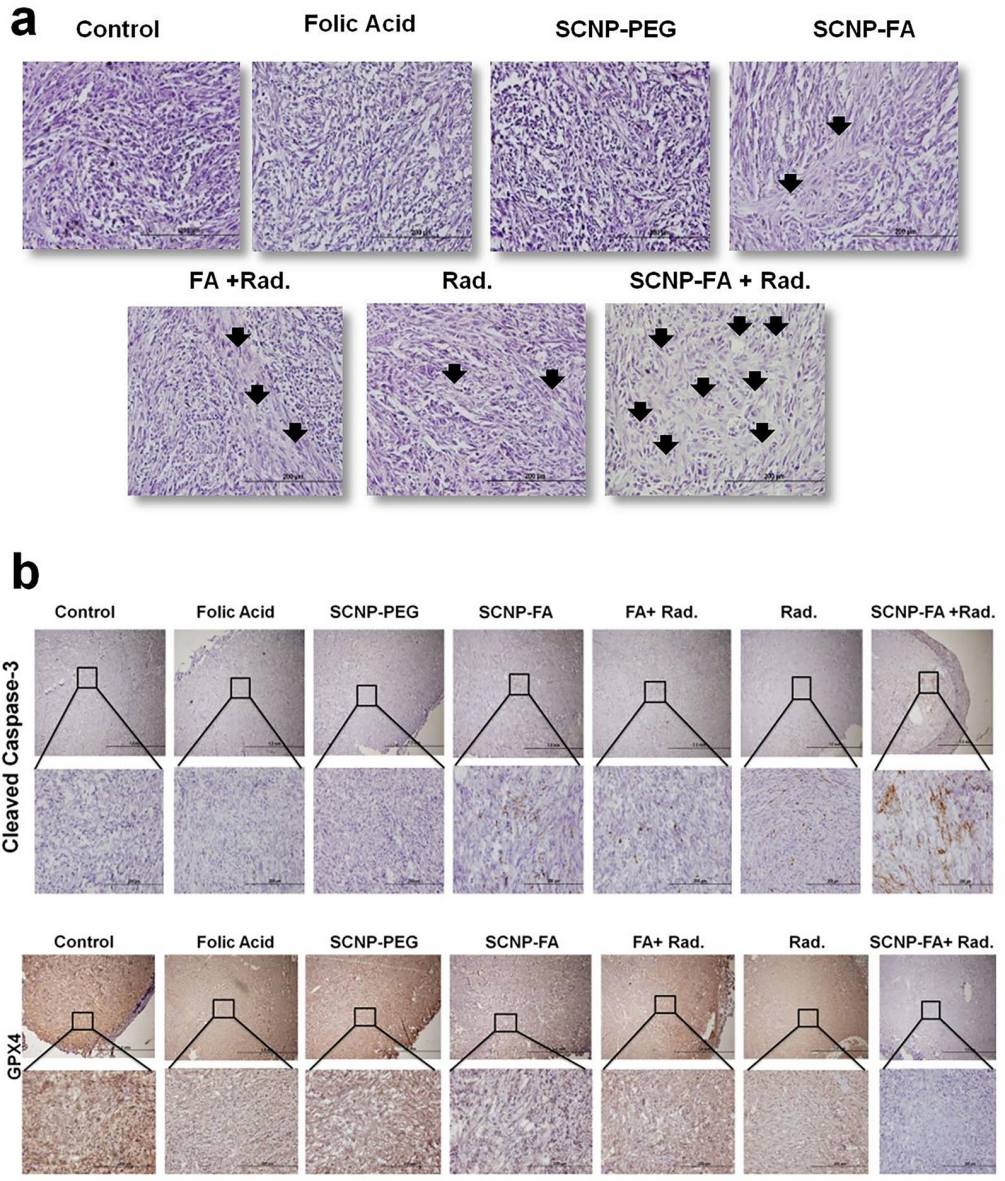
Immunohistochemical staining performed to determine the cell death. (**a**) H&E staining of tumor tissues shows the cell features upon various treatments. The black arrowheads indicate cell death. The scale bar stands for 200 μm. (**b**) IHC staining of tumor tissues was performed to determine the expression of cleaved caspase-3 and GPX4 upon different treatments. The magnified image confirms the location of cleaved caspase-3 in the cytoplasm and perinuclear region and the GPX4 in the cytoplasm and nucleus. The scale bar stands for 1.0 mm and 200 μm

After confirming the potential results in vitro through this effective treatment, resulting in cell death, we further investigated this treatment in vivo. The PAN02 subcutaneous model was used, and nanoparticles/drugs were intravenously injected. After the tumor volume reached 100 mm [3]the treatment with nanoparticles/drug was administered thrice a week and exposed to radiation depending upon the desired treatment group (Fig. 7a). A biodistribution study was performed in vivo to confirm the accumulation of SCNP-FA in the tumor. The nanoparticle accumulation was monitored at different time intervals (0, 2, 4, 8, 24, 48 and 72 h) by injecting the nanoparticles intravenously in mice. It was observed that the highest accumulation of nanoparticles occurred at 4 h. The liver and spleen also take up the nanoparticles, but at a much lower rate than the tumor site (Fig. 7b). Nearly no nanoparticles were detected in the heart, lungs, and kidneys. It was also observed that after 24 h, the nanoparticles were cleared from the liver, which ensures good pharmacokinetics [51], minimizing long-term accumulation in major organs. This rapid elimination significantly reduces the risk of systemic toxicity. Moreover, this nanoparticle surface is functionalized with PEG chains, which are well-known to enhance biocompatibility, reduce opsonisation, and prevent non-specific uptake by the mononuclear phagocyte system. The transient circulation and subsequent clearance of these nanoparticles indicate a favourable pharmacokinetic profile, reducing potential off-target effects and ensuring minimal interferences with physiological processes. Different treatments were examined in this subcutaneous model, which included- Control, folic acid (300 nM), SCNP-PEG (1000 ppm), SCNP-FA (1000 ppm), FA (300 nM) + Rad. (0.5 Gy), Rad. (0.5 Gy) and SCNP-FA (1000 ppm) + Rad. (0.5 Gy). After 4 h of nanoparticle administration, the radiation was exposed when the accumulation was higher. It was found that the radiation dose of 0.1 Gy was not effective in the in vivo treatment studies, as observed in the in vitro studies, because of the aggressive nature of the tumor proliferating at a very high rate and therapeutic resistance, as the cells alone were not able to mimic the exact tumor microenvironment. To address this, we selected a slightly higher dose of 0.5 Gy, carefully balancing between achieving sufficient FA release at the tumor site and avoiding damage to the surrounding healthy tissues. This intermediate dose proved effective in triggering therapeutic responses without introducing significant off-target toxicity. Therefore, to enhance the therapeutic efficacy, 0.5 Gy was used for the in vivo experiments and to further explore the tumor microenvironment. The tumor volume in control and SCNP-PEG did not change, but the folic acid, Rad., FA + Rad., and SCNP-FA showed a small decrease in tumor volume. Notably, the tumor volume was significantly reduced in SCNP-FA + Rad. compared to the other treatment groups, mitigating the rapid tumor growth (Fig. 7c, d). The tumor weights were also measured after the end-point of the study, which showed a decrease after SCNP-FA + Rad. treatment compared to other treatment groups (Fig. 7e, f). The body weights of all the mice were measured every 2 days, and no influential changes were observed upon treatment (Fig. 7g). Next, the liver, spleen, heart, lungs, and kidneys were examined to determine the toxicity in the non-tumor part of the organs after 14 days of treatment. All the treated groups showed no inflammation or tissue changes (Figure S20). The serum and urine biochemical tests were also performed to determine liver and kidney function, revealing no significant difference in all the groups (Figure S21). The results indicate good biocompatibility and biosafety of nanoparticles, ensuring no toxic effects. The hematoxylin and eosin (H&E) staining for the tumor sections was also performed to determine cell integrity and tissue morphology. It was observed that control, FA, and SCNP-PEG had no obvious effect, but minimal changes were observed in Rad., FA + Rad., and SCNP-FA. It was observed that the tissue morphology was damaged, and cell death occurred in SCNP-FA + Rad., which was visible through the abundant loss of nuclei (Fig. 8a). Next, the cell death markers for programmed cell death and ferroptosis were checked by Cleaved Caspase-3 and GPX4 in the tumor sections, respectively. It was found that the expression of cleaved caspase-3 was higher in SCNP-FA + Rad. in the cytoplasmic and perinuclear regions compared to the other treatment groups. The expression level of GPX4 was inhibited in the SCNP-FA + Rad. and observed in the nuclear and cytoplasmic regions. This indicates that the cell death occurred due to the release of FA from the nanocarrier upon radiation, which, thereby, bound to the folate receptor of pancreatic cancer and resulted in the generation of a high amount of ROS, which led to lipid peroxidation **(**Fig. 8b**)**. These results demonstrated that SCNP-FA + Rad. can inhibit tumor growth and potentially treat pancreatic cancer.

### Nanoparticle-induced radiation treatment remodeled the immune microenvironment to inhibit pancreatic tumor growth

**Fig. 9:**
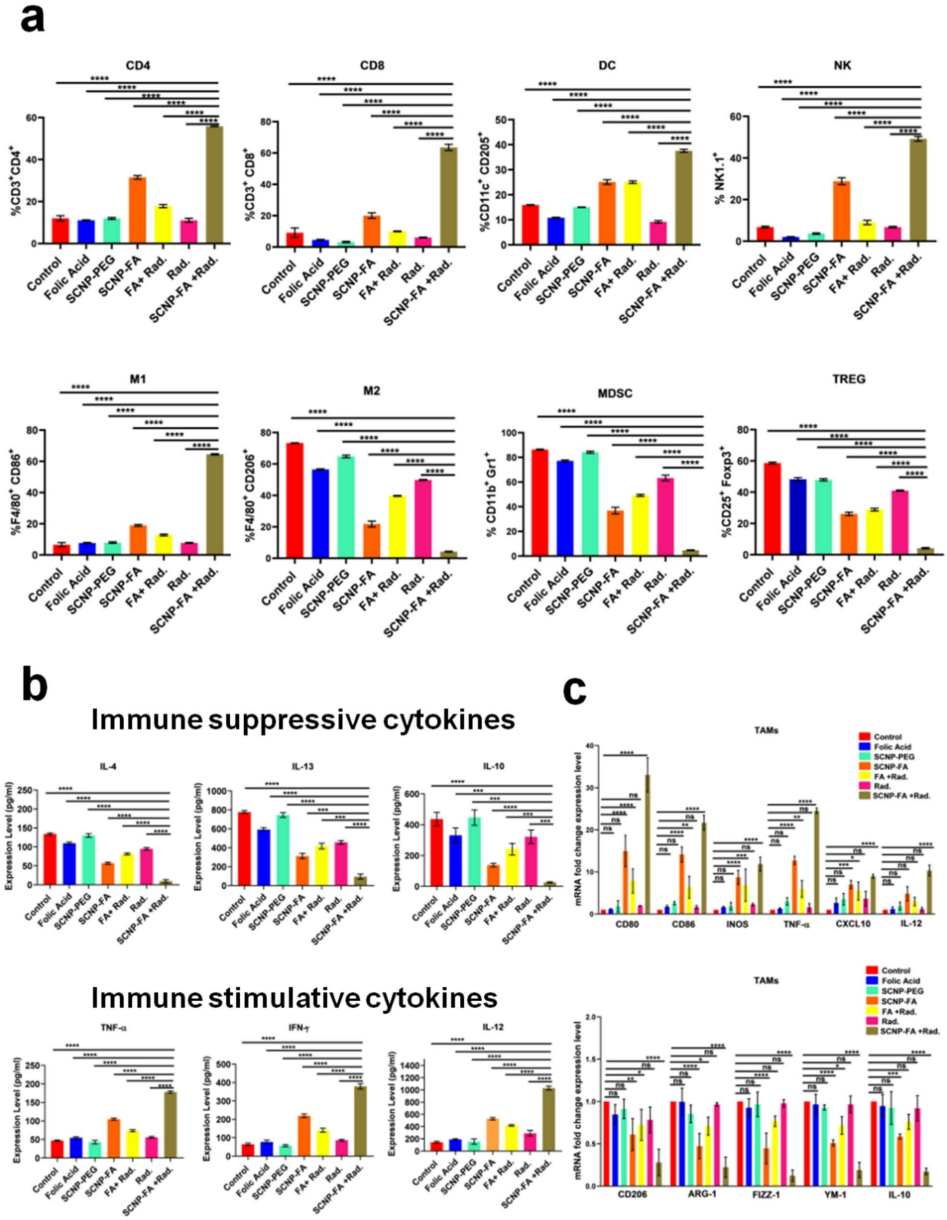
SCNP-FA-induced radiation treatment remodels the immune microenvironment to suppress pancreatic cancer. (**a**) Immune cells such as CD4 + T cells, CD8 + T cells, DCs, NK cells, M1 TAMs, M2 TAMs, MDSCs and Tregs detected in the pancreatic tumor microenvironment by flow cytometric analysis. (**b**) Secretion of immune suppressive cytokines (IL-4, IL-13, and IL-10) and immune stimulative cytokines (TNF-α, IFN-γ, IL-12) residing in the pancreatic tumor microenvironment by ELISA. (**c**) mRNA expression level of immune suppressive and immune stimulative genes in PAN02 TAMs, followed by different treatment groups, detected by qPCR (*n* = 3). Statistical significance was determined by Student’s t-test and ordinary two-way ANOVA. Data are presented as mean ± SD (*P < 0.05, **P < 0.01 *** *P* < 0.001, ****P < 0.0001)

**Fig. 10:**
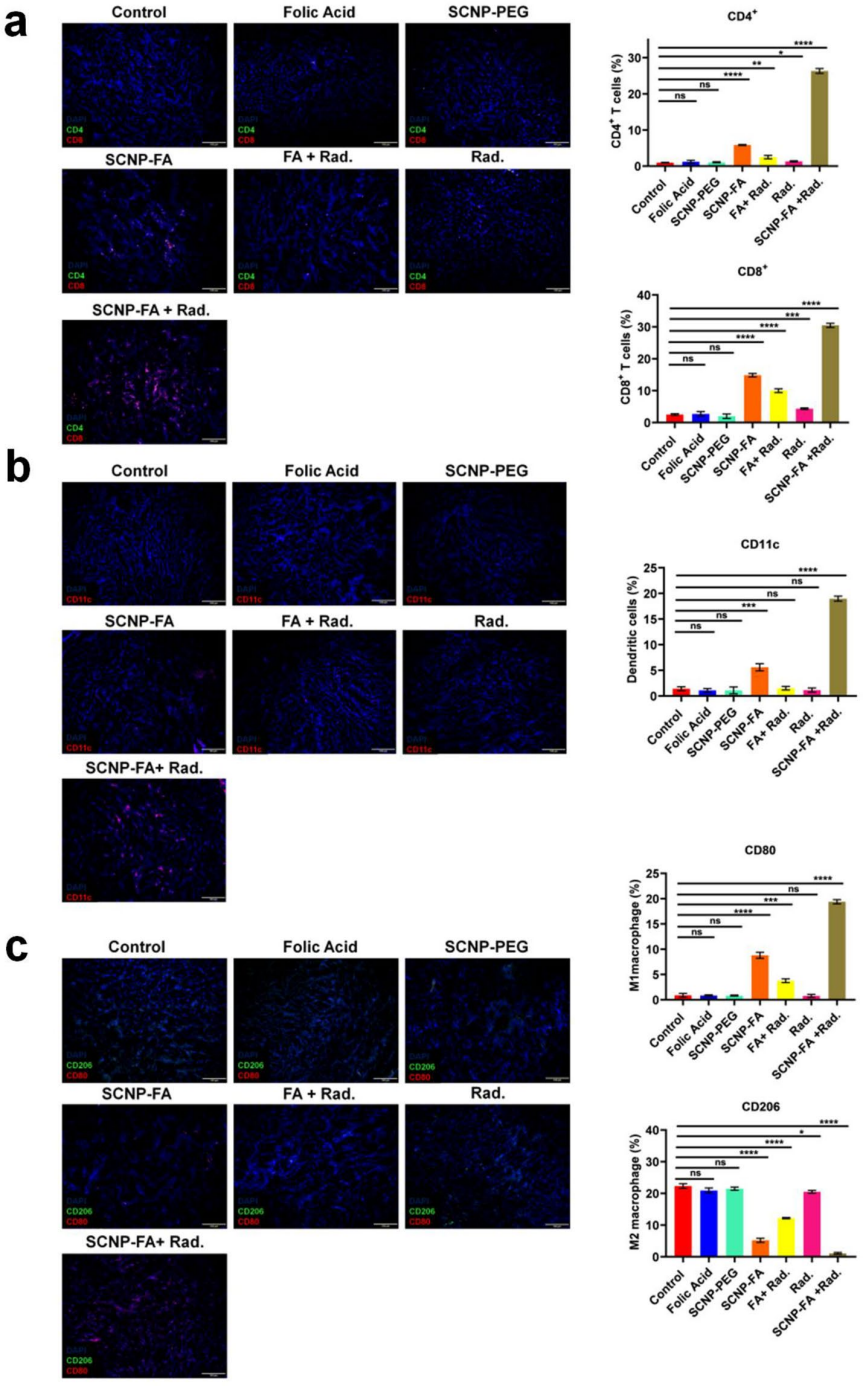
Immunofluorescence staining reveals a strong immune response. (**a**) Expression of CD4 + and CD8 + T cells (**b**) Expression of CD11c, dendritic cells (**c**) Expression of CD80, M1 TAMs and CD206, M2 TAMs within the pancreatic tumor microenvironment upon different treatments. The scale bar stands for 100 μm. Statistical significance was determined by Student’s t-test. Data are presented as mean ± SD (*P < 0.05, **P < 0.01 *** *P* < 0.001, ****P < 0.0001)

Tumor microenvironment (TME) is a vast and complex environment composed of cancer cells, stroma, abundant populations of immune cells, blood and dense lymphatic vasculature, and various cellular components [52]. The crosstalk between the cancer cells and their microenvironment is a key regulator for tumor growth. Precisely, the unhealthy/dying cancer cells communicate with the immune cells through the release of multiple signals during ferroptosis, thereby being helpful in the modulation of anti-tumor immune responses [53, 54]. Studies have shown that CD8 + T cells kill cancer cells due to the induction of ferroptosis and can sensitize tumor cells by producing IFN-γ and exerting an anti-tumor effect [55–57]. The shift from M2 to M1 possesses a strong anti-tumor response of tumor-associated macrophages (TAMs) determined for phagocytosis and tumor inhibition. Studies have confirmed that targeted ferroptosis in TAMs helps eliminate pro-tumorigenic M2 macrophages and reprograms TAMs into tumoricidal M1 macrophages, leading to tumor suppression [58–60]. To evaluate whether SCNP-FA + Rad. treatment exerts an immunoregulatory effect; the various populations of immune cells inside the tumor microenvironment were determined through flow cytometry. The immune stimulative cells, including CD8 + T cells, CD4 + T cells, Dendritic cells (DC), Natural killer cells (NK), and M1 tumor-associated macrophages were up-regulated. In contrast, myeloid-derived suppressor cells (MDSCs), T regulatory cells (Tregs), and M2 tumor-associated macrophages were down-regulated upon the SCNP-FA + Rad. Treatment. Comparatively, SCNP-FA alone showed a minimal effect on the immune cells, while the other groups showed no apparent effect **(**Fig. 9a, S22-26**)**. Accordingly, the immune stimulatory cytokines, including TNF-α, IFN-γ, and IL-12, were significantly increased. In contrast, the immune suppressive cytokines, including IL-4, IL-13, and IL-10, were significantly decreased upon SCNP-FA + Rad. treatment detected through enzyme-linked immunosorbent assay (ELISA) (Fig. 9b). Furthermore, to confirm the gene expression levels of TAMs upon the therapy, a quantitative polymerase chain reaction (qPCR) was performed. It was found that the mRNA expression levels of genes for the M1 phenotype, such as CD86, CD80, iNOS, TNF-α, CXCL-10, and IL-12, were up-regulated, whereas the genes for the M2 phenotype, such as CD206, ARG-1, FIZZ-1, YM-1, and IL-10 were down-regulated upon the SCNP-FA + Rad. treatment compared to the other treatment groups (Fig. 9c). Next, immunofluorescence staining was performed for the immune cells, including T cells, DC cells, and M1 and M2 tumor-associated macrophages, to examine the histology sections for immune infiltration. It was observed that the CD8 + T cells, CD4 + T cells, and DCs cells were abundantly present in SCNP-FA + Rad. treatment group compared to the other groups. The M1 marker, CD80, showed increased accumulation, while the M2 marker, CD206, decreased upon SCNP-FA + Rad. treatment (Fig. 10a-c). Collectively, these results indicate SCNP-FA + Rad. treatment led to repolarizing the M2 phenotype to the M1 phenotype and remodeling the immune microenvironment to suppress pancreatic cancer development.

## Conclusion

In summary, our study emphasized that the folic acid-controlled release from the nanocarrier upon radiation can provoke immunogenic cell death in murine and human pancreatic cells through ferroptosis and programmed cell death. In addition, nanoparticles conjugated with the drug penetrated inside the cell through endocytosis, targeting the folate receptor overexpressed in pancreatic cancer. This study is a comprehensive exploration of the interconnected mechanisms that are responsible for tumor cell death. It involves the use of nanoparticles for the controlled drug release of folic acid to target the overexpressed folate receptors on the cancer cells through receptor-mediated endocytosis. It does not use the conventional ferroptosis inducer, such as erastin or RSL3, which are more commonly used in studies for ferroptosis-induced cell death; whereas, here, the folic acid indirectly contributes to ferroptosis through modulation of cellular redox homeostasis and metabolic stress pathways, particularly with X-ray irradiation. Next, it uncovers that the major cause of cell death is through ferroptosis, which is linked to immunogenic cell death, causing release of DAMPs such as CRT and HMGB1. The cellular homeostasis is also affected, determining the main powerhouse of energy, mitochondria, which are disrupted and cause dysfunction in the cellular processes. The interrelated processes involved in ferroptosis have been explored, such as cellular homeostasis, protein markers, and morphological changes. In addition to this, the tumor microenvironment has been studied in a detailed manner. It gives us a clear picture of how the remodeling of the immunosuppressive tumor microenvironment happens upon nanoparticle-induced radiation treatment. Notably, the remodeling of immune cells inside the tumor microenvironment, manifested with an increase of CD8 + T cells, CD4 + T cells, DC cells, NK cells, M1 TAMs, and a decrease of Tregs, MDSCs, and M2 TAMs upon the treatment, led to inhibition of tumor growth. This study exhibits a promising approach to treating pancreatic cancer with the controlled release of folic acid from the nanocarrier to improve its therapeutic efficacy and trigger immune responses. Future work could integrate fluorophore-labeled FA or FRET-based reporters to non-invasively monitor release kinetics in vivo, to realize FA release half-life in tumor versus normal tissues, which would better characterize the targeting specificity, and further dissect the detailed mechanism [61]. The translational application of this study may go beyond pancreatic cancer; in other cancer types, overexpressing folate receptors is linked with an immunosuppressive microenvironment.

## Experimental methods

### Chemical reagents

Igepal C0-520, sodium oleate, ethanol, hexane, oleic acid (90%), 1- octadecene (90%) Tetraethyl orthosilicate (TEOS), ammonium hydroxide (NH_3_), piperidine (> 99%), 3 Aminopropyltriethoxysilane (99%) (APTES), 1(3 Dimethylaminopropyl)−3- ethylcarbodiimide hydrochloride (98%) (EDC), N Hydroxysulfosuccinimide sodium salt (> 98%) NHS, ammonium fluoride (> 99.99%), lithium hydroxide (99.995%), yttrium III chloride (99.9%), cerium chloride (99%), Amino PEG acid, MW3400, Fmoc-2- nitro-L-phenylalanine (> 99.5%), Folic acid were purchased from Sigma Aldrich, J.J Baker, Acros Organics, Alfa Aesar, Nanocs, Chemimpex and Cayman chemical respectively. DMEM, RPMI medium, and FBS were purchased from Gibco. Erastin (B1524, ApeXBio), Ferrostatin-1(HY-100579), Z-Vad-FMK (HY-1665), Necrostatin-1 (HY-15760), 3-methyladenine (HY-19312) were purchased from MedChemExpress. Mitotracker green, H2DCFDA dye, and C11- Bodipy dye were purchased from Invitrogen. Live and dead staining was performed with calcein AM and Ethidium homodimer-1 purchased from Thermofisher Scientific. The JC-1 assay kit was purchased from MedChemExpress to determine mitochondrial membrane potential. Annexin V- FITC apoptosis detection kit was purchased from Pharmigen to analyze apoptotic cells through flow cytometry.

### Characterization of nanoparticles

The images of the nanoparticles’ size and shape were taken using the Hitachi H-7500 transmission electron microscope (TEM). Fourier transform infrared spectroscopy (FTIR) spectra were taken by JASCO 200E FTIR. HR-TEM (High-resolution) images were taken by JEOL JEM-2100 F Cs STEM. Photoluminescence (PL) was performed by Fluoromax-4 spectrofluorometer (Horiba Scientific). Dynamic light scattering and zeta potential measurements were performed by Zetasizer 3000 HS-Advanced. Ultraviolet-visible (UV- vis) spectra were taken with UV-Vis spectrometer Hewlett-Packard 8453. XRD was performed using an X-ray diffractometer, Shimadzu XRD-7000 S. The concentration of Yttrium in nanoparticles was recorded with Icap^™^ 7400 ICP-OES, Thermo Scientific. The absorbance readings for the MTT analysis were recorded with a continuous wavelength microdisk analyzer (Molecular devices Spectramax 340PC384).

### Cell lines with culture conditions

PAN02, PANC-1, and ASPC-1, mouse and human pancreatic cells were cultured in DMEM and RPMI-1640 medium, respectively. All the cells were maintained in high glucose DMEM or RPMI-1640, with 10% FBS and 1% P/S/A (antibiotic solution) at 37 °C in a humidified atmosphere of 5% CO2. The cells were seeded at their respective density to achieve 70–80% confluence, followed by the next day. The cells were incubated with/without nanoparticles depending on their treatment group, for 24 h, followed up on the second day. On the third day, the cells were washed with 1X PBS twice and replaced with fresh medium. Later on, they were exposed to the radiation dose depending upon their respective treatment group.

### Animal studies

C57BL/6 male mice (6–8 weeks old) were used for all the animal studies. The Pan02 cells (mouse pancreatic cells) were dissolved in 50 µl of solvent (1:1 PBS with matrigel). The injection of the 10^7^ cancer cells was subcutaneously implanted with a 0.3 ml 30G insulin needle in the ventral flank of mice. The tumor size was measured as volume (mm^3^) = 1/2 (length x width [2]), and after 10 days, the tumor volume reached between 100 and 120 mm [3]; therefore, the mice later started receiving the treatment. All animal treatments and procedures were approved by the Institutional Animal Care and Use Committee (IACUC NO. 112240) of National Cheng Kung University (NCKU), and animal care was provided in accordance with the Laboratory Animal Welfare Act and the Guide for Care and Use of Laboratory Animals.

### Synthesis of LiYF_4_: Ce^3+^nanoparticles (SCNPs)

All the protocols for the synthesis of nanoparticles were followed from the previously published paper. The Y(OA)_3_/Ce(OA)_3_ complex was added to 16 ml of oleic acid, 5 ml of octadecene, and 2 ml of hexane in the round-bottom flask. It was further subjected to heating at 150 °C at a rate of 2 °C/min for 1 h and 30 min and cooled to about 50 °C. Further, LiOH and NH_4_F were dissolved in the fresh 10 ml of methanol solution and added to the ongoing reaction, which was kept on continuous stirring. The reaction mixture was subjected to heating of 70 °C at a rate of 2 °C/min for the complete evaporation of methanol and then the temperature was raised to 110 °C at a rate of 1 °C/min for about 1 h and next, for another 1 h for the degas with the continuous purging of the argon gas. Later, the reaction was heated at 310 °C at a rate of 10.5 °C/min for 1 h 30 min, maintained in argon gas. Later, the cooling off of the thermal decomposition reaction was done, the SCNPs were first washed with ethanol twice and then twice with ethanol and hexane in the ratio of 4:1. Finally, they were dispersed in 10 ml of hexane. The conjugation of folic acid (FA) to the nanoparticles was achieved using EDC-NHS chemistry. The FA standard solution was prepared in DMSO and stored at 4 °C in dark conditions. The standard curve was plotted by taking readings at eight concentrations of FA dissolved in 1 M NaOH solution. The concentrations were from 0.003mM to 0.05mM and measured by the UV spectrophotometer. The three independent experiments were performed to prove the reproducibility of the data. The maximum absorbance intensity was observed at a 256 nm peak and was plotted with the respective FA concentrations to obtain an analytical curve. The calibration equation was calculated using the linear fit regression analysis.

### Folic acid release triggered by X-ray

The SCNP-FA (1000 ppm) was added in the middle of a 35 mm culture dish, and each dish was subjected to different dosages of X-ray from 0 to 6 Gy by RS 2000 X-ray irradiator (Rad Source Technologies, GA). After the X-ray, the nanoparticles were collected in an eppendorf tube and centrifuged at 15,000 rpm for 15 min to collect nanoparticles from the photo-cleaved FA. The supernatant was dissolved in IM NaOH to determine the FA concentration in each group using a UV spectrophotometer.

### Cytotoxicity studies in vitro

The mouse and human pancreatic cell lines- PAN02, PANC-1, and ASPC-1 cells were treated with nanoparticles to evaluate the cytotoxicity in the cells. The PAN02 (3 × 10^3^), PANC-1 (4 × 10^3^), and ASPC-1 (5 × 10^3^) cells were seeded in a 96-well assay plate and incubated at 37 °C overnight. The next day, the cells were treated with the nanoparticles, depending on their different concentrations, and incubated for 24 h. The next day, the cells were washed with 1X PBS twice and replaced with fresh medium with the MTT reagent (1:10), which was added to each well and incubated for 3 hrs at 37 °C. Later on, the medium was replaced with 150µL DMSO for dissolving formazan. The absorbance readings were taken at 492 nm by a microplate reader spectrophotometer. The colony assay was performed by seeding cells in 6-well culture plates where PAN02 (500), PANC-1 and ASPC-1 (1000) cells were seeded each per well and incubated for 24 h at 37 °C with 5% CO_2_. The next day, the cells were treated with the nanoparticle conjugated with folic acid for another 24 h. After 24 h, the wells were washed out with 1X PBS twice, fresh medium was added to each well, and the plates were taken for the irradiation treatment at different doses (0.1 Gy, 0.3 Gy, 0.5 Gy, 1 Gy, and 2 Gy) by RS 2000 X-ray irradiator (Rad Source Technologies). The colonies for the irradiated groups were compared with the control group without adding SCNP-FA and irradiation treatment. After the irradiation treatment, the cells were incubated for 10–12 days to form colonies. When the colonies were developed, they were first washed with 1X PBS, and then methanol was added for 10 min to fix the colonies. Later, the colonies were stained with a crystal violet solution for 1 h, and then the colonies were washed with distilled water and dried. The colonies were scanned, and they were counted through the ImageJ software.

### Western blotting analysis

The cell lysates were prepared, and equal amounts of protein were loaded into each lane of 10% SDS-PAGE gel. After electrophoresis, the proteins were transferred to a PVDF membrane (Millipore) for 2 h. The membranes were blocked with 5% skim milk for 1 h at room temperature, then was washed thrice with TBST buffer for 10 min each, and then the primary antibodies were added for overnight incubation at 4 °C. The next day, the HRP-conjugated secondary antibodies were added and incubated for 1 h at room temperature, which were then washed with TBST buffer thrice for 10 min each. The blots were developed using ECL blotting reagents.

### Antibodies

In vitro antibodies used were FRα (Abcam, ab221543), FRβ (Genetex, 105822), β- Actin (Novus, NB600-501), Cox-2 (SantaCruz, sc19999), CD71 (Cell Signaling, 13113 and Genetex, GTX102596), 4-Hydroxynonenal (R&D, MAB3249-SP), GPX4 (Abclonal, A1933), FIS1 (Proteintech, 10956-1-AP), OPA1 (Proteintech, 27733-1-AP), DRP-1 (Proteintech, 12957-1-AP), MFN-1 (Proteintech, 13798-1-AP), Calreticulin (Proteintech, 10292-1-AP), HMGB1 (Proteintech, 10829-1-AP), Cleaved Caspase-3 (Cell Signaling Technology, 9661 S), Alexa Fluor 594 anti-mouse CD80 (Biolegend, 104754), Alexa Fluor 594 anti-mouse CD8a (Biolegend, 100758), Alexa Fluor 647 anti-mouse CD11c (Biolegend, 117312), Alexa Fluor 488 anti-mouse CD206 (Biolegend, 141710), Alexa Fluor 488 (Thermofisher, LFR53-0041-82), CD45 anti-mouse (Biolegend, 103130), NK 1.1 anti-mouse (Biolegend, 108708), CD3 anti-mouse (Biolegend, 100204), CD8 anti-mouse (Biolegend, 100708), CD4 anti-mouse (Biolegend, 100405), F4/80 anti-mouse (Biolegend, 123109), CD86 anti-mouse (Biolegend, 105005), CD206 anti-mouse (Biolegend, 141705), CD11c anti-mouse (Biolegend, 117310), CD205 anti-mouse (Biolegend, 138214), CD25 anti-mouse (Biolegend, 101910), Foxp3 anti-mouse (Biolegend, 126404), CD11b anti-mouse (Biolegend, 101207), Gr1^+^ anti-mouse (Biolegend, 108406).

### Fluorescence microscopy

The PAN02 (5 × 10^3^), PANC-1 (15 × 10^3^) and ASPC-1 (3 × 10^4^) cells were seeded in the 35 mm or PAN02 (1 × 10^5^), PANC-1 (2 × 10^5^) and ASPC-1 (3 × 10^5^) in 6-well culture dishes. After the cells were treated with nanoparticles and irradiated, the cells were washed twice with PBS, stained with 2µM of JC-1 dye, and incubated for about 15–20 min at 37 °C. The images were captured using an inverted microscope to determine the mitochondrial membrane potential (MMP). The cells incubated with 100nM of mitotracker green dye for 15–20 min at 37 °C were captured using a confocal microscope FV3000 (Olympus) to determine the intracellular localization of mitochondria. The live and dead staining was performed with calcein-AM and ethidium homodimer-1 dye, where the cells were incubated for about 30 min at 37 °C, and images were captured. The images were quantified using ImageJ software to determine the red/green fluorescence intensity and mitochondrial footprint using ImageJ with Fiji software.

### Flow cytometry analysis

For in vitro experiments, cells were seeded in a 6-cm culture dish where PAN02 (1 × 105), Panc-1 (3 × 105), and ASPC-1 (5 × 10^5^) cells were incubated overnight. After 24 h, the cells were treated with the nanoparticle or different treatment groups. The next day, the cells were washed with 1X PBS twice and replaced with fresh medium followed by respective dose according to the cell line. The cells were treated with 25µM of H2DCFDA dye to determine the cellular ROS at different time intervals of 0.5 h, 3 h, and 6 h for 30 min and were trypsinized, centrifuged, and washed with PBS twice. Similarly, the 5µM of C11-BODIPY lipid peroxidation dye was added to cells in serum-free medium for 30 min, and later were trypsinized, centrifuged, and washed with PBS twice. The determination of cellular apoptosis was performed with the Annexin V- FITC apoptosis detection kit. For in vivo experiments, cells were treated with the designated antibodies such as CD45, NK 1.1, CD3 CD8, CD4, F4/80, CD86, CD206, CD11c, CD205, CD25, Foxp3, CD11b and Gr1^+^ to determine the population of CD4, CD8 T cells, NK cells, DCs, MDSCs, Tregs, M1 TAMs and M2 TAMs. The cells were finally resuspended in 1X PBS, analyzed by the Cell Quest Pro software (BD Biosciences), and detected on the BD FACS Calibur machine (BD Biosciences).

### Biodistribution analysis

The tumor-bearing Pan02 mice were treated with 100 µl, 1000 ppm of SCNP-FA dissolved in PBS via tail vein injection. The treatment was done for 0, 2, 4, 8, 24, 48, and 72 h, and later, the mice were sacrificed to detect the accumulation of nanoparticles in the tumor and other organs (lung, liver, heart, kidney, and spleen) of the body. The organs and tumor were washed twice with PBS and stored in 4% paraformaldehyde solution for 1 day at 4 °C. The samples were dissected into small pieces, digested in aqua regia for 1 week, and kept in a fume hood. Later, they were filtered into a fresh tube, and the yttrium concentration was determined by ICP-MS. All the data were obtained in triplicate.

### Biosafety of SCNP-FA in vivo

C57BL/6 male mice (6–8 weeks old) were administered with 100 µl of PBS or SCNP-FA (1000ppm) through tail vein injection. The body weight was monitored daily for about 7 days after the treatment. Later, the mice were sacrificed to collect blood and urine from the control and treated groups for serum and biochemical analysis. All the organs (heart, liver, lung, kidney, spleen, and pancreas) were collected and subjected to H&E staining to observe histological changes.

### Serum and biochemical analysis

The blood was withdrawn from the heart of mice, and heparin sodium was added to it immediately. The blood samples were centrifuged at 1300 rpm for 10 min at 4 °C to separate serum from the blood. The serum samples were used for biochemical analysis to determine total bilirubin (T-Bil), alkaline phosphatase (ALP), aspartate aminotransferase (AST/GOT), alanine aminotransferase (ALT/GPT) for hepatic inflammation index, blood urea nitrogen (BUN), creatine (CRE) and uric acid (UA) for kidney function index by FUJI DRI-CHEM 4000 (Fujifilm). The urine from mice was collected by the metabolic cage and centrifuged at 3000 rpm for 10 min to separate the impurities. The urine samples were used to determine protein, albumin, creatinine, pH value, glucose, bilirubin, blood, ketone bodies, nitrite, and leukocyte by RT-4010 analyzer (Arkray). All the results were taken in triplicate.

### H&E staining

All organs (heart, liver, lung, kidney, pancreas, and spleen) and tumors were embedded in paraffin, and the sections were obtained at a thickness of 5 μm. The sections were deparaffinized with xylene, rehydrated with 100%, 95%, and 75% ethanol, washed with PBS, and stained for 45 s with hematoxylin solution. The sections were washed with tap water thrice and stained for 10 min with eosin solution. Later, the sections were immersed, followed by 75% ethanol, 95% ethanol, and xylene, and air-dried to mount the medium with a coverslip for visualization. The sections were visualized under the Olympus BX51 microscope, and three different areas were captured for each group.

### Immunohistochemistry staining

The tumor samples were embedded in paraffin, and the sections were obtained at 5 μm thickness. The sections were deparaffinized, rehydrated, and incubated overnight with the target primary antibody at 4 °C. The next day, incubation with secondary antibody and washing with PBS twice and staining was performed with an ABC Peroxidase Standard Staining kit (Thermofisher Scientific) and washed with PBS twice, followed by staining with DAB Peroxidase Substrate Kit (Vector Laboratories) according to the manufacturer’s protocol and washed with PBS and double distilled water twice. Later, the sections were stained for 45 s with hematoxylin solution and rinsed with tap water. The sections were air-dried and mounted for visualization under the Olympus BX51 microscope, and three different areas were captured for each group.

### Anti-tumor effect in vivo

PAN02 tumor-bearing mice were subjected to different treatments according to their respective groups, and 100 µl of the treatment was administered via tail vein injection thrice per week. In the SCNP-FA + X- ray group, the mice were irradiated with 0.5 Gy after 4 h of administration according to the highest accumulation of nanoparticles in the tumor site by RS2000 X-ray irradiator (Rad Source Technologies). After the treatment, the mice were monitored every other day for body weight and tumor volume. The mice were sacrificed, and all the tumors and organs (heart, liver, lung, spleen, and kidney) were collected to confirm the biosafety after the treatment, as well as blood and urine samples were collected to determine the serum and biochemical analysis. All the results were obtained in quadruplicates.

### Transmission electron microscopy for the treatment of cells with nanoparticles

The PAN02 and RAW264.7 cells were treated with nanoparticles alone, and nanoparticles were exposed to radiation for 24 h. After the cells were washed with PBS twice and were fixed in a fixative solution comprised of 2.5% glutaraldehyde and 3 mM CaCl_3_ in 0.1 M cacodylate buffer for about 1 h at room temperature. The cells were washed with 0.1 M cacodylate buffer, and then the cells were fixed for 1 h in 0.1 M cacodylate buffer comprised of 1% osmium tetroxide and 1.5% potassium ferricyanide at 4 °C. The cells were washed gently with distilled water, followed by dehydration in graded ethanol of 70%, 90%, and 95% for about 15 min each, and finally, 100% for about three times and 30 min each. After dehydration, the cells were infiltrated into the stages with spur resin-ethanol solutions comprising 50%, 75%, and 100% resin, and each stage was exposed for about 1 h. After infiltration, the cells were kept in 100% spur resin overnight. The cells were embedded in the fresh resin and polymerized at 70 °C for about 24 h. The embedded cells were cut with an ultramicrotome (Ultracut S, Leica Reichart) into 70 nm ultrathin sections with a diamond knife. The sections were stained with uranyl acetate and lead citrate. The images were taken by JEOL JEM-1400 at 120 keV, equipped with a CCD camera (Ultrascan, Gatan).

### Enzyme-linked immunosorbent assay (ELISA) analysis

The fresh tumor samples were washed with PBS, and homogenates comprising protease inhibitors (Merck) were obtained in PBS with the help of a pestle. The homogenate solution was further subjected to centrifugation by 3 K centrifugal tubes (Pall Corporation), and the solution was used for the detection of various cytokines such as mouse IL-10 Duoset ELISA (#DY417, R&D systems), mouse IL-4 Duoset ELISA (#DY404, R&D systems), mouse TNF-α Duoset ELISA (#DY410, R&D systems), mouse IL-12 Duoset ELISA (#DY419, R&D systems), mouse IL-13 Duoset ELISA (#DY413, R&D systems), mouse IFN-γ Duoset ELISA (#DY485, R&D systems), mouse CXCL10/IP-10 Duoset ELISA (#DY466, R&D systems) according to the manufacturer’s protocol.

### Immunofluorescence staining

The tumor samples were washed with PBS for 1 day and fixed in 4% paraformaldehyde for 2 days, followed by washing with PBS and subjected to 10%, 20%, 30%, and 35% sucrose for 2 days each at 4 °C. The tumor samples were dried gently to drain out the solution, and then the OCT solution was added to the samples and kept at −20 °C overnight to get them frozen. The sections were cut with the help of cryostat, and the sections were later washed with PBS thrice for 5 min each and then blocked with 1% BSA (Merck) in TBST for 1 h at room temperature. The sections were stained with the target antibodies such as Alexa Fluor^®^594 anti-mouse CD80 (#104754, Biolegend), Alexa Fluor^®^488 anti-mouse CD206 (#141710, Biolegend), Alexa Fluor^®^594 anti-mouse CD8 (#100758, Biolegend), Alexa Fluor^®^488 anti-mouse CD4 (#LFR53-0041-82, Thermofisher Scientific), Alexa Fluor^®^647 anti-mouse CD11c (#117312, Biolegend), 4 × 6- diamidino-2phenylindole (DAPI) (#D3571, Thermofisher Scientific) for 1 h. The sections were rewashed with PBS to remove the extra staining dye and air-dried gently. The sections were finally covered with the coverslip and the mounting medium. The images were obtained under the Olympus BX51 microscope, and three different fields were taken for each treatment group, which were analyzed using Image J software.

### Co-culture method for TAMs

The 2 × 10^5^ RAW264.7 cells were seeded in the 6 cm culture dish overnight. Next day, the cells are treated with IL-13 (20ng/ml) (# C02015, CroyezBioscience) and IL-4 (20ng/ml) (#C02006, CroyezBioscience) for 48 h to obtain M2 macrophages and treated with IFN-γ (20ng/ml) (#C02055, CroyezBioscience) and LPS (100ng/ml) (#916374, Merck) for 24 h to obtain M1 macrophages. The 1 × 10^5^ RAW264.7 cells were seeded in a 6-well plate, and 2 × 10^4^ PAN02 cells were seeded in a 0.4 μm pore transparent PET membrane (#353090, Corning) for another 24 h separately. The inserts with PAN02 cells and the 6-well plate with RAW264.7 cells were combined upon treatment. The drug or nanoparticles were added to the inserts and 6 well plates. After 24 h, the nanoparticles and drug were washed out with PBS and exposed to radiation depending upon the treatment groups for another 24 h.

### RNA isolation and real-time quantitative PCR

For lysis, the cells were washed with ice-cold PBS first and gently scratched with the cell scrapper upon adding Trizol (#15596026, Thermofisher Scientific). Next, chloroform was added and vortexed before incubation on ice. The centrifugation was done to obtain the three different layers separating the colorless upper aqueous phase containing RNA. For isolation of RNA, the isopropanol was added to the tube, incubated on ice, and centrifuged to obtain a small white-gel-like pellet at the bottom. The pellet was washed with 75% ethanol, vortexed, and centrifuged. The supernatant was discarded, and the RNA pellet was air-dried to remove any ethanol in the tube. The pellet was dissolved in 20 µl nuclease-free water and incubated in the water bath at 45–50 °C for 10–15 min. The absorbance values of RNA were obtained on Ultra- microvolume spectrophotometer (Thermo, Nanodrop 2000c) at 260/280nm. Next, for cDNA synthesis, the reaction mix was created with an iScript cDNA synthesis kit (#1708891, BioRad), and the reaction was run in a BioRad T100 thermal cycler. For, quantitative polymerase chain reaction (qPCR) was performed with KAPA SYBR FAST, ABI PRISM TM (#KK4603, Merck) in Step One Plus Real-Time PCR machine (Thermo Fisher Scientific). The primers used were CD80, CD86, INOS, TNF-α, CXCL10/IP-10, IL-12, CD206, ARG1, FIZZ-1, YM-1, IL-10 and GADPH. The expression level of the targeted gene was normalized with the expression level of GADPH.

### Metabolic energy analysis

The PAN02 cells were seeded in the plate; the hydrate cartridge channels were activated overnight at 37 °C. The next day, the drug was added to the wells, and the materials were purchased from XFe24 FluxPaks (#102340100, Agilent Technologies) and performed using Seahorse XFe24 Analyzer (Agilent Technologies) to measure oxygen consumption rate (OCR) according to the manufacturer’s protocol. The drug treatments involved 5µM oligomycin (#HY-N6782, MedChemExpress), 2.5µM FCCP (#HY-100410, MedChemExpress), 2.5µM rotenone (HY-B1756, MedChemExpress) and antimycin A (#A8674, Merck). The OCR readings were calculated for basal respiration, ATP production, maximum respiration, and spare respiratory capacity.

### Statistical analysis

All the experiments were performed independently in triplicates and were expressed as mean ± standard deviation (SD). The graphs were plotted using the OriginPro 8.5 software, and images were quantified using ImageJ software. All the statistical analysis was performed using GraphPad Prism software. The significant differences between treated and control groups were calculated using Student’s t-test and two-way ANOVA, *P < 0.05, **P < 0.01, ***P < 0.001, **** *P* < 0.0001. For pairwise comparisons between two groups, an unpaired two-tailed Student’s t-test was used. For comparisons involving two independent variables, like different treatments and different time points, a two-way ANOVA was used, followed by Tukey’s multiple comparisons test to identify specific group differences. A P value of < 0.05 will be considered statistically significant.

## Supplementary Information

Supplementary Material 1

## Data Availability

No datasets were generated or analysed during the current study.
