# Supplementary material for: Radiocleavable rare-earth nanoactivators targeting over-expressed folate receptors induce mitochondrial dysfunction and remodel immune suppressive microenvironment in pancreatic cancer
Source: J Nanobiotechnology. 2025 Aug 12;23:562. doi: 10.1186/s12951-025-03657-8 (PMC12341285; doi:10.1186/s12951-025-03657-8)
Supplement: Supplementary file 1 — Supplementary Material 1 [file 12951_2025_3657_MOESM1_ESM.docx]

**Radiocleavable Rare-Earth Nanoactivators targeting Over-expressed Folate receptors induce Mitochondrial Dysfunction and Remodel Immune Suppressive Microenvironment in Pancreatic Cancer**

***Tanvi Gupta^1^, Shang-Rung Wu^2^, Li-Chan Chang^1^, Forn-Chia Lin^3^, Yan-Shen Shan^1, 4^, Chen-Sheng Yeh ^5, 6, 7^, Wen-Pin Su^1, 6, 8, 9*^***

^1^Institute of Clinical Medicine, College of Medicine, National Cheng Kung University, Tainan 704, Taiwan

^2^School of Dentistry & Institute of Oral Medicine, College of Medicine, National Cheng Kung University, Tainan City, 701, Taiwan

^3^Department of Radiation Oncology, National Cheng Kung University Hospital, College of Medicine, National Cheng Kung University, Tainan 704, Taiwan

^4^Department of Surgery, National Cheng Kung University Hospital, College of Medicine, National Cheng Kung University, Tainan 704, Taiwan

^5^Department of Chemistry, National Cheng Kung University, Tainan 701, Taiwan

^6^Center of Applied Nanomedicine, National Cheng Kung University, Tainan 704, Taiwan

^7^Department of Medicinal and Applied Chemistry, Kaohsiung Medical University, Kaohsiung 807, Taiwan

^8^Departments of Oncology and Internal Medicine, National Cheng Kung University Hospital, College of Medicine, National Cheng Kung University, Tainan 704, Taiwan

^9^Clinical Medicine Research Center, National Cheng Kung University Hospital, College of Medicine, National Cheng Kung University, Tainan 704, Taiwan

**^*^Corresponding Author:**

**Prof. Wen-Pin Su, Institute of Clinical Medicine, College of Medicine, National Cheng Kung University, No. 35, Rd. Xiaodong, Tainan 704, Taiwan.**

**Email:** [**wpsu@mail.ncku.edu.tw**](mailto:wpsu@mail.ncku.edu.tw)

**Table of Contents**

Supplementary Figure 1. Elemental Mapping and Selected area diffraction pattern (SAED)...**4**

Supplementary Figure 2. HR-TEM image of synthesized nanoparticles…………………........**5**

Supplementary Figure 3. FA drug concentration and quantification……………………...…..**6**

Supplementary Figure 4. Raman spectroscopy showing different vibrational groups in free FA drug, SCNP-PEG, and SCNP-FA…………………………………………………………..….**7**

Supplementary Figure 5. Stability of SCNP-FA under different physiological conditions…...**8**

Supplementary Figure 6. Cellular uptake of NPs in different cancer cell lines…………….....**9**

Supplementary Figure 7. Colony assay for determining cell death in human pancreatic cell lines…………………………………………………………………………………………..**10**

Supplementary Figure 8. SCNP-FA-induced radiation treatment promotes ferroptosis……..**11**

Supplementary Figure 9. Intracellular ROS production via ferroptosis…………………**12-13**

Supplementary Figure 10. Lipid ROS generation via ferroptosis…………………………...**14**

Supplementary Figure 11. Determining cell death via ferroptosis…………………………..**15**

Supplementary Figure 12. Determining mitochondrial membrane potential (MMP, ΔΨm)...**16**

Supplementary Figure 13. Determining cell death by live/dead staining…………………...**17**

Supplementary Figure 14. Morphological changes in cells induce ferroptosis……………..**18**

Supplementary Figure 15. Mitochondrial morphological changes upon treatment………...**19**

Supplementary Figure 16. Mitochondrial dynamics induces ICD……………………….....**20**

Supplementary Figure 17. Statistical Analysis of Seahorse OCR and ECAR after treatment in tumor-associated macrophages……………………………………………………………....**21**

Supplementary Figure 18. Determining mitochondrial membrane potential (MMP, ΔΨm)...**22**

Supplementary Figure 19. Mitochondrial morphological changes upon treatment in PAN02 TAMs………………………………………………………………………………………...**23**

Supplementary Figure 20. Biosafety confirms no apparent inflammation…………………..**24**

Supplementary Figure 21. Biochemical analysis confirms no apparent toxicity…………...**25**

Supplementary Figure 22. Gating Strategy by flow cytometry .…………………………….**26**

Supplementary Figure 23. Gating of CD4+ and CD8+ T cells……………………………....**27**

Supplementary Figure 24. Gating of DCs and M1 TAMs cells……………………………...**28**

Supplementary Figure 25. Gating of M2 TAMs and MDSCs cells………………………….**29**

Supplementary Figure 26. Gating of Tregs and NK cells…………………………………....**30**

Supplementary Figure 27. Quantification of Western Blots………………………………....**31**

Supplementary Figure 28. Quantification of Western Blots ...……………………………....**32**

Supplementary Figure 29. Intracellular ROS production with SCNP-PEG shows no significant change.…………………........................................................................................**33**


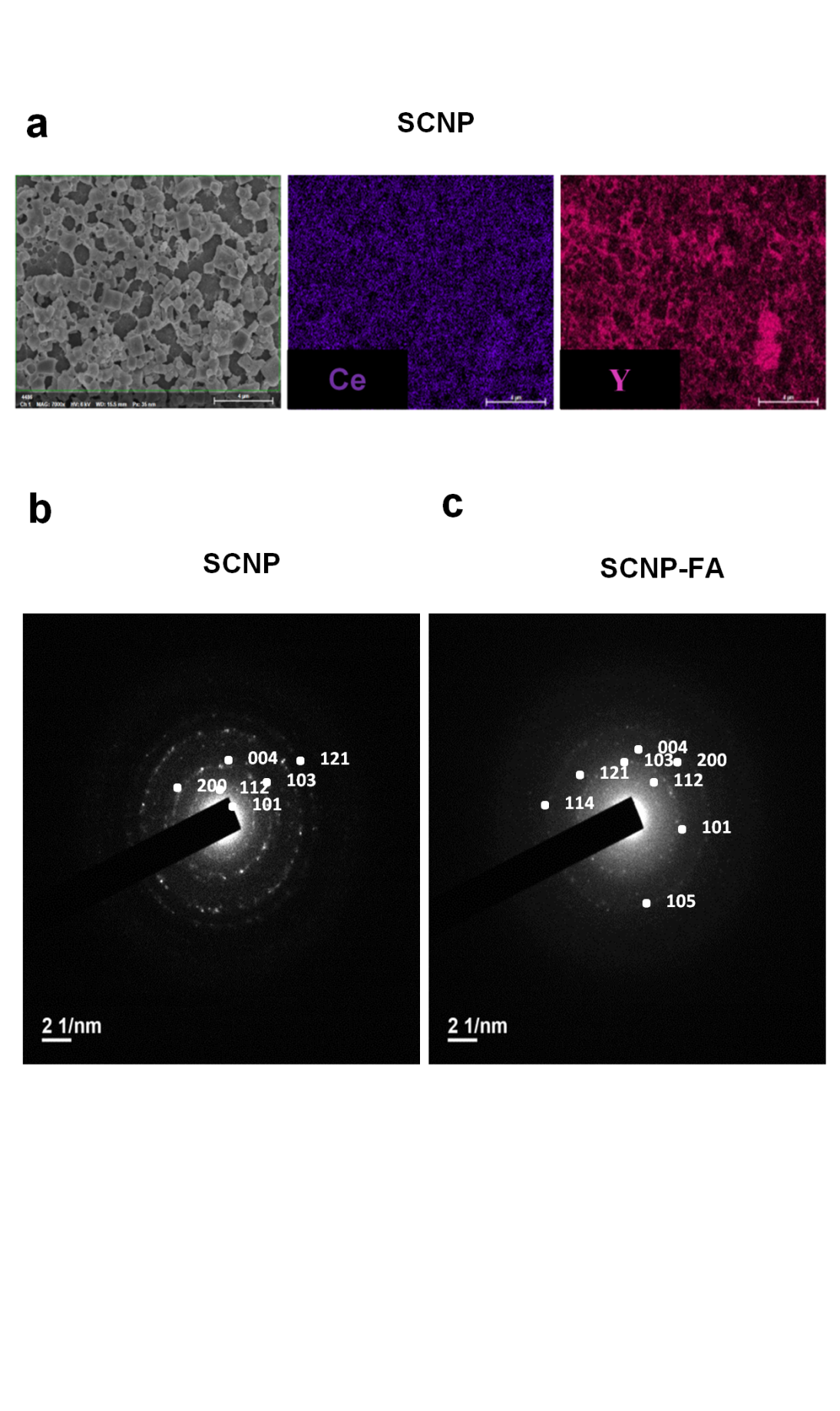


**Supplementary Figure 1. Elemental Mapping and Selected area diffraction pattern (SAED).** (a) Elemental mapping of SCNP (b) SAED pattern of SCNP (c) SAED pattern of SCNP-FA.


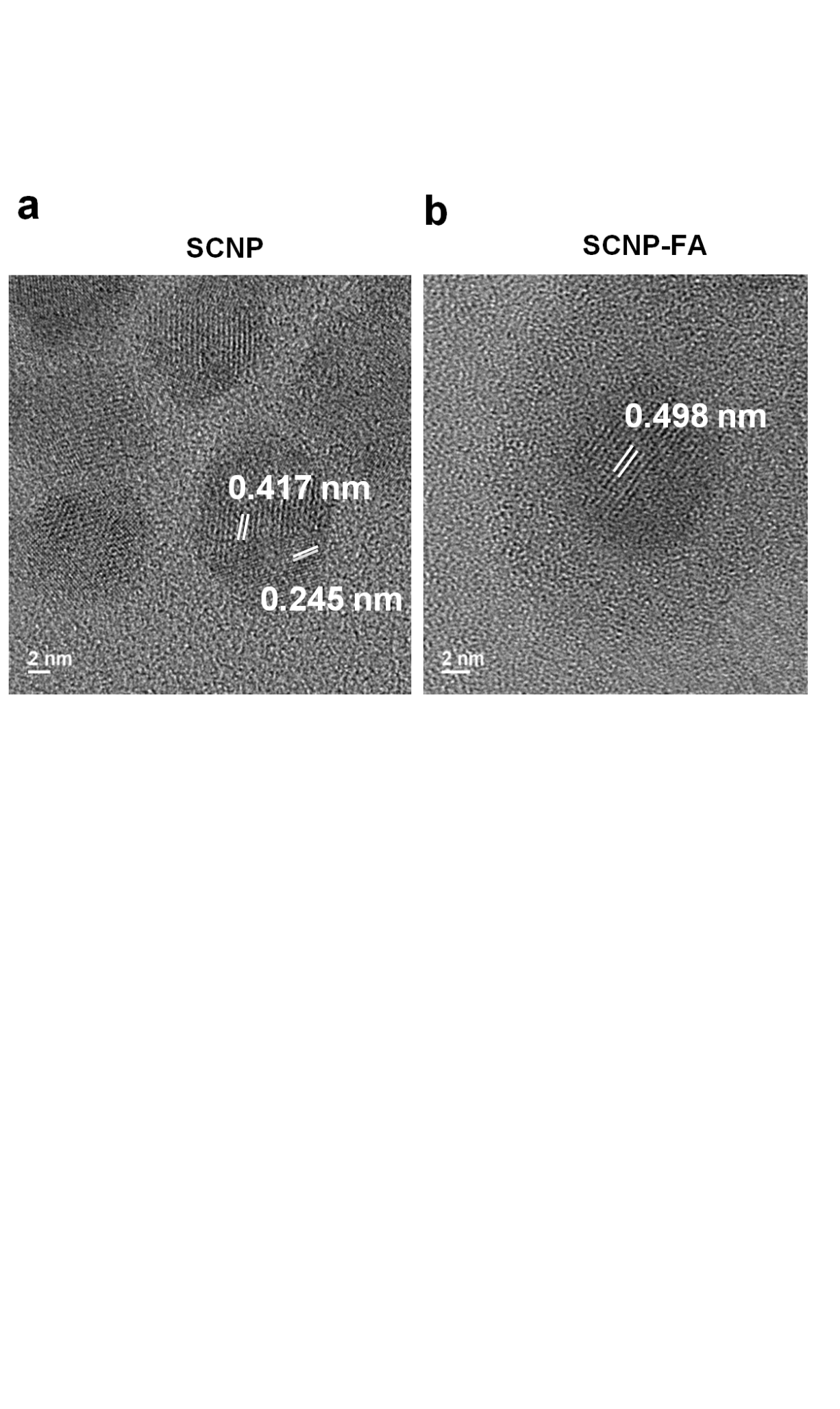


**Supplementary Figure 2. HR-TEM image of synthesized nanoparticles.** (a) SCNP (b) SCNP-FA shows a crystalline pattern with interplanar spacing.


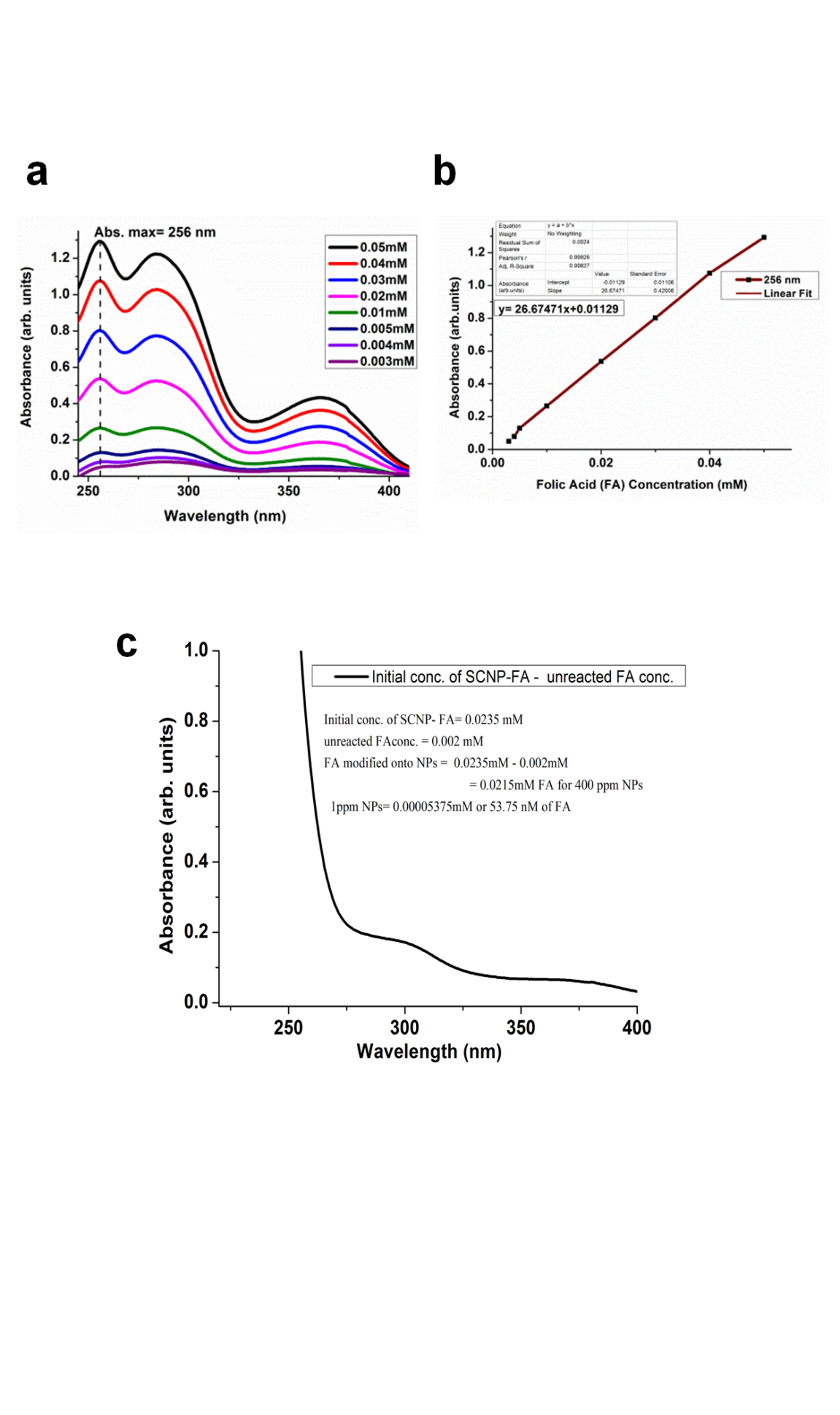


**Supplementary Figure 3. FA drug concentration and quantification.** (a) UV absorbance spectrum determining the absorbance of FA at different concentrations. (b) Linear fitting for a standard for FA concentration from the absorbance spectrum. (c) Quantification of FA in SCNP-FA by absorbance spectrum.


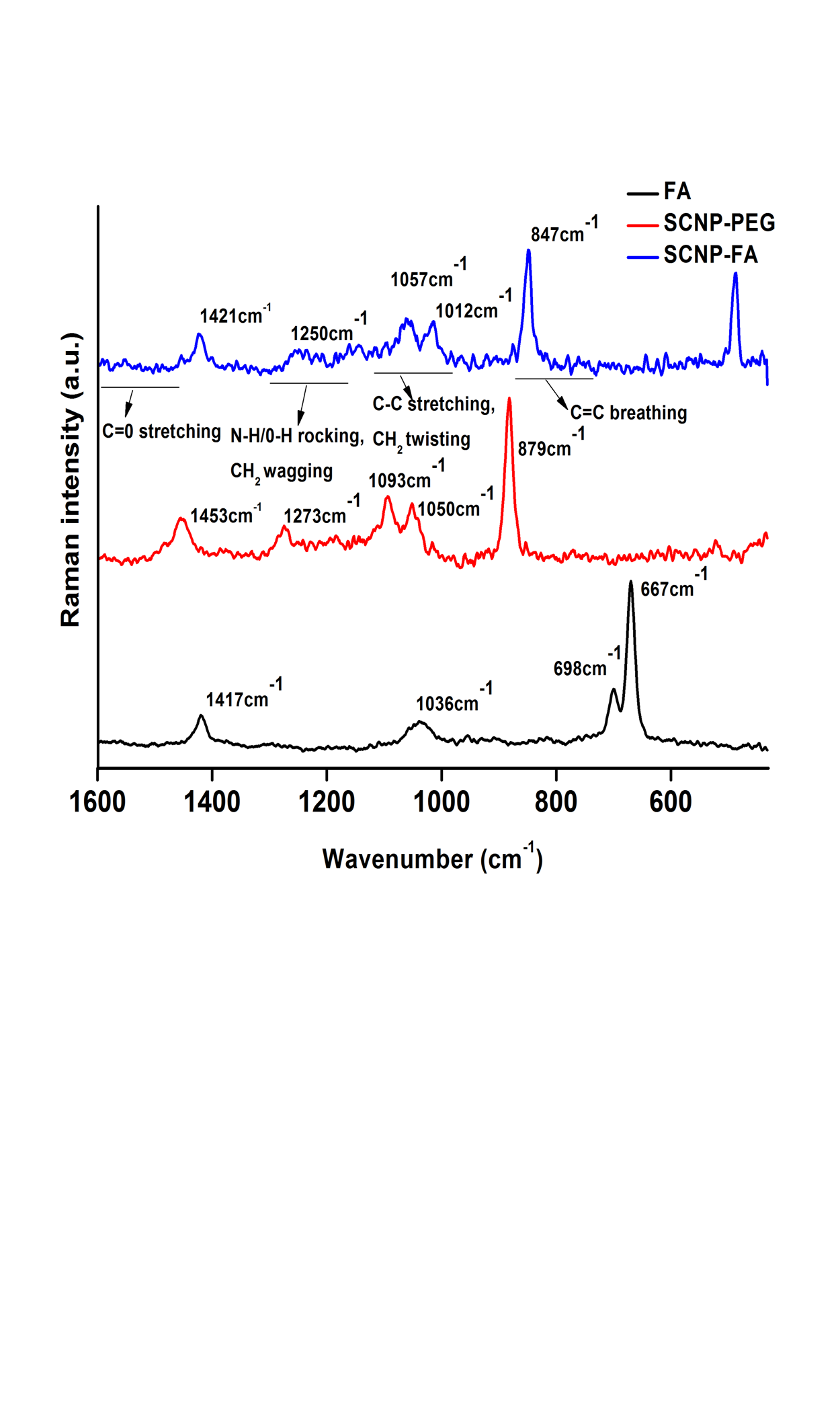


**Supplementary Figure 4.** **Raman spectroscopy showing different vibrational groups in free FA drug, SCNP-PEG and SCNP-FA.**


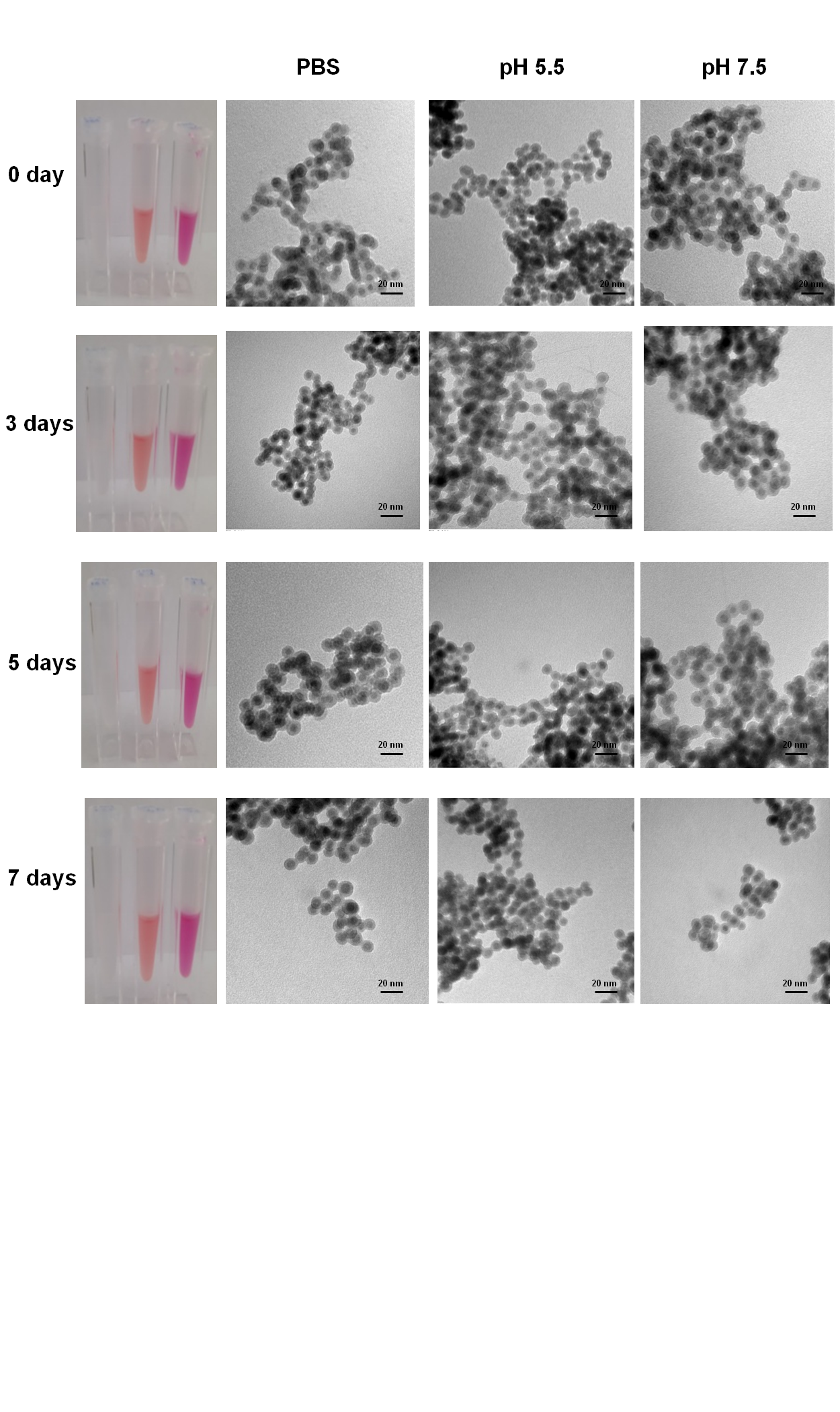


**Supplementary Figure 5. Stability of SCNP-FA under different physiological conditions.**

NPs were dispersed in PBS, culture medium of pH 5.5 and pH 7.5 in an eppendorf tube and incubated at 37°C for 1 week. The images show no observational changes in color or morphological changes through TEM.


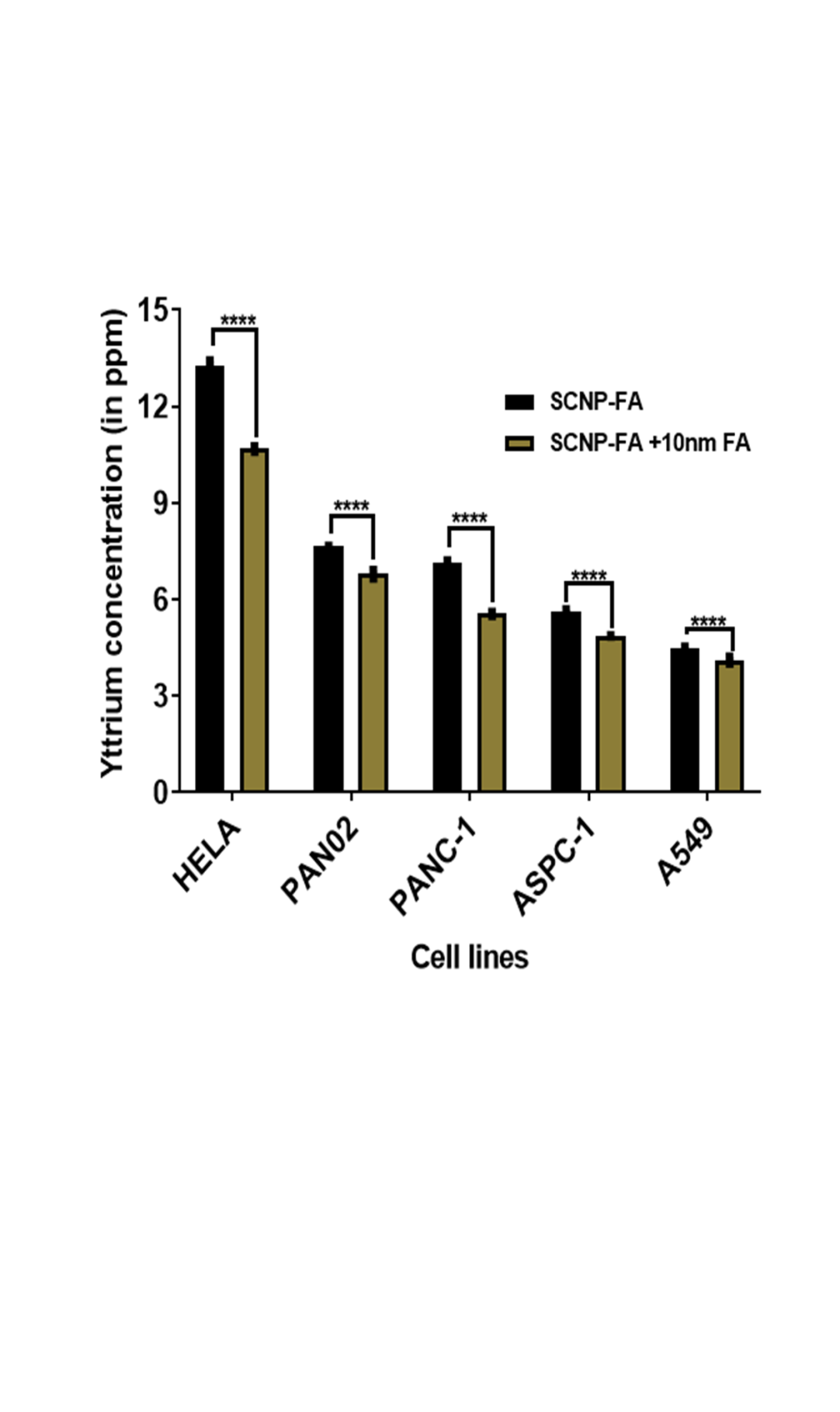


**Supplementary Figure 6. Cellular uptake of NPs in different cancer cell lines.** Y^3+^ concentration determined by ICP upon treatment of different cell lines with SCNP-FA and excess FA for 24 hrs. Data are presented as mean± SD (****P< 0.0001).


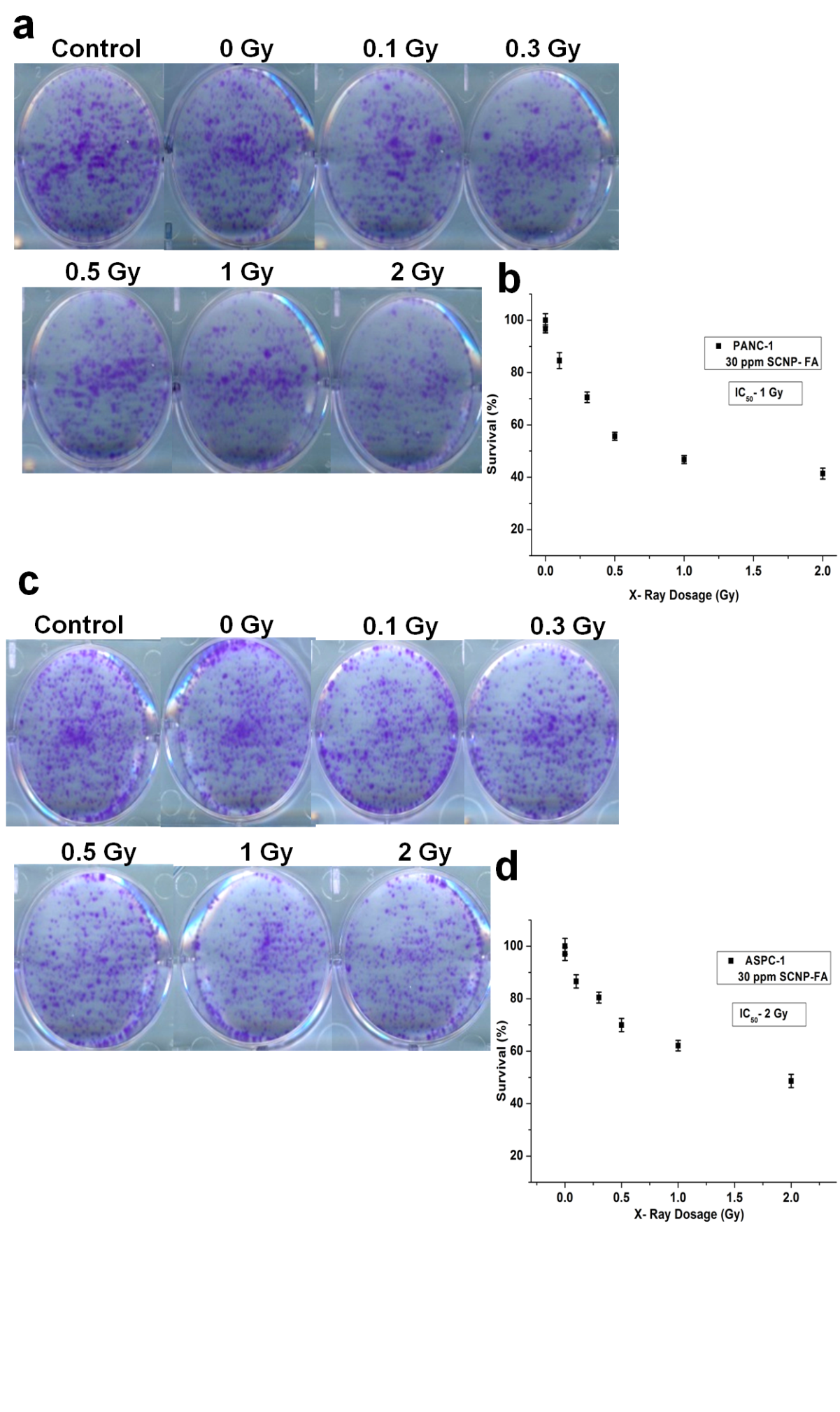


**Supplementary Figure 7. Colony assay for determining cell death in human pancreatic cell lines.** (a) PANC-1 treated with SCNP-FA induced radiation (b) Survival percentage of PANC-1 with IC_50_ of 1 Gy (n=3). (b) ASPC-1 treated with SCNP-FA induced radiation (d) Survival percentage of ASPC-1 with IC_50_ of 2 Gy (n=3).


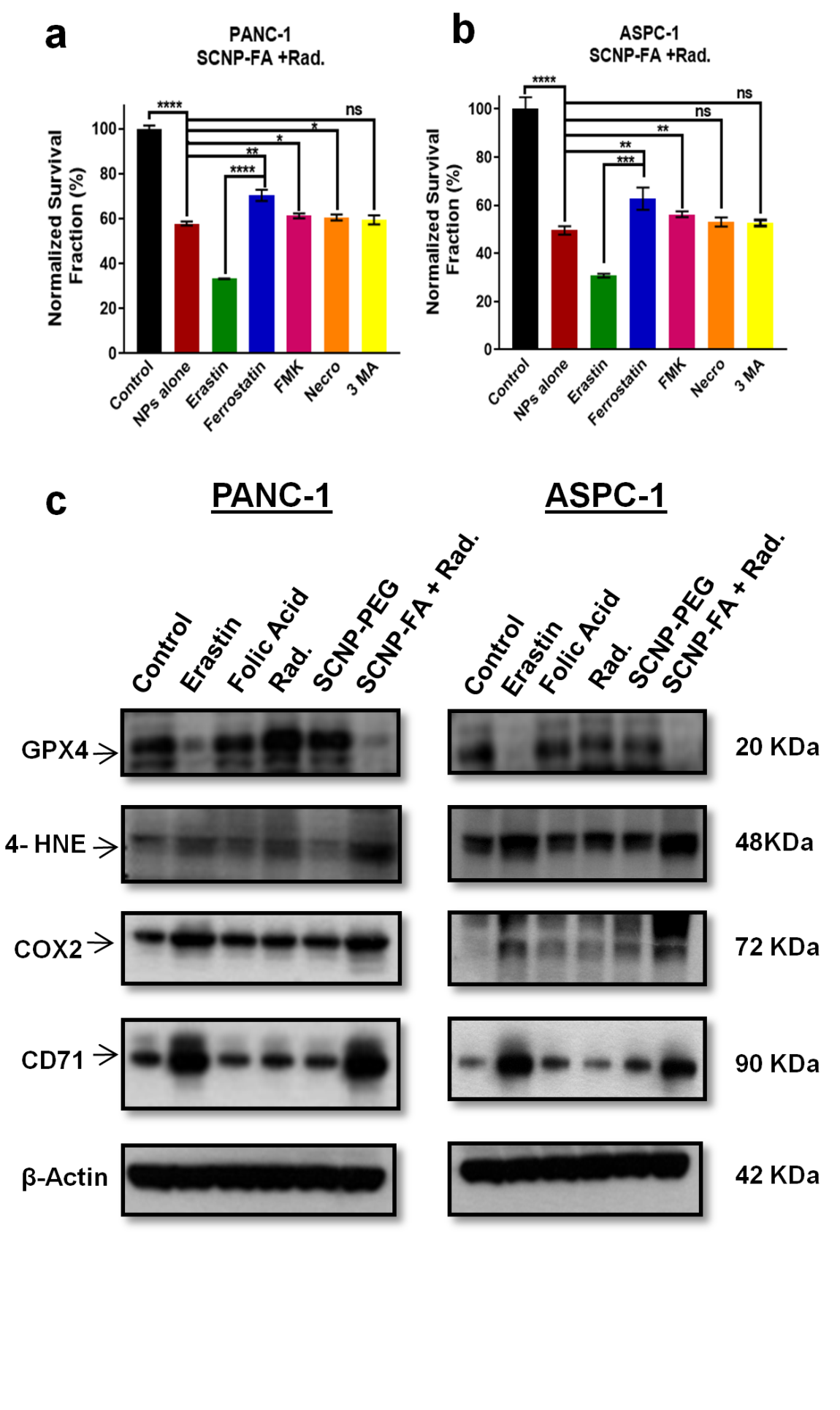


**Supplementary Figure 8.** **SCNP-FA-induced radiation treatment promotes ferroptosis.** (a) Different cell death inhibitors in PANC-1 treated with SCNP-FA induced radiation (n=3). (b) Different cell death inhibitors in ASPC-1 treated with SCNP-FA induced radiation (n=3). (c) Protein expression levels of different markers of ferroptosis in PANC-1 after various treatments. (d) Protein expression levels of different markers of ferroptosis in ASPC-1 after various treatments. Erastin acts as a positive control (n=3). Data are presented as mean± SD (*P <0.05, **P< 0.01, *** P<0.001, ****P< 0.0001).


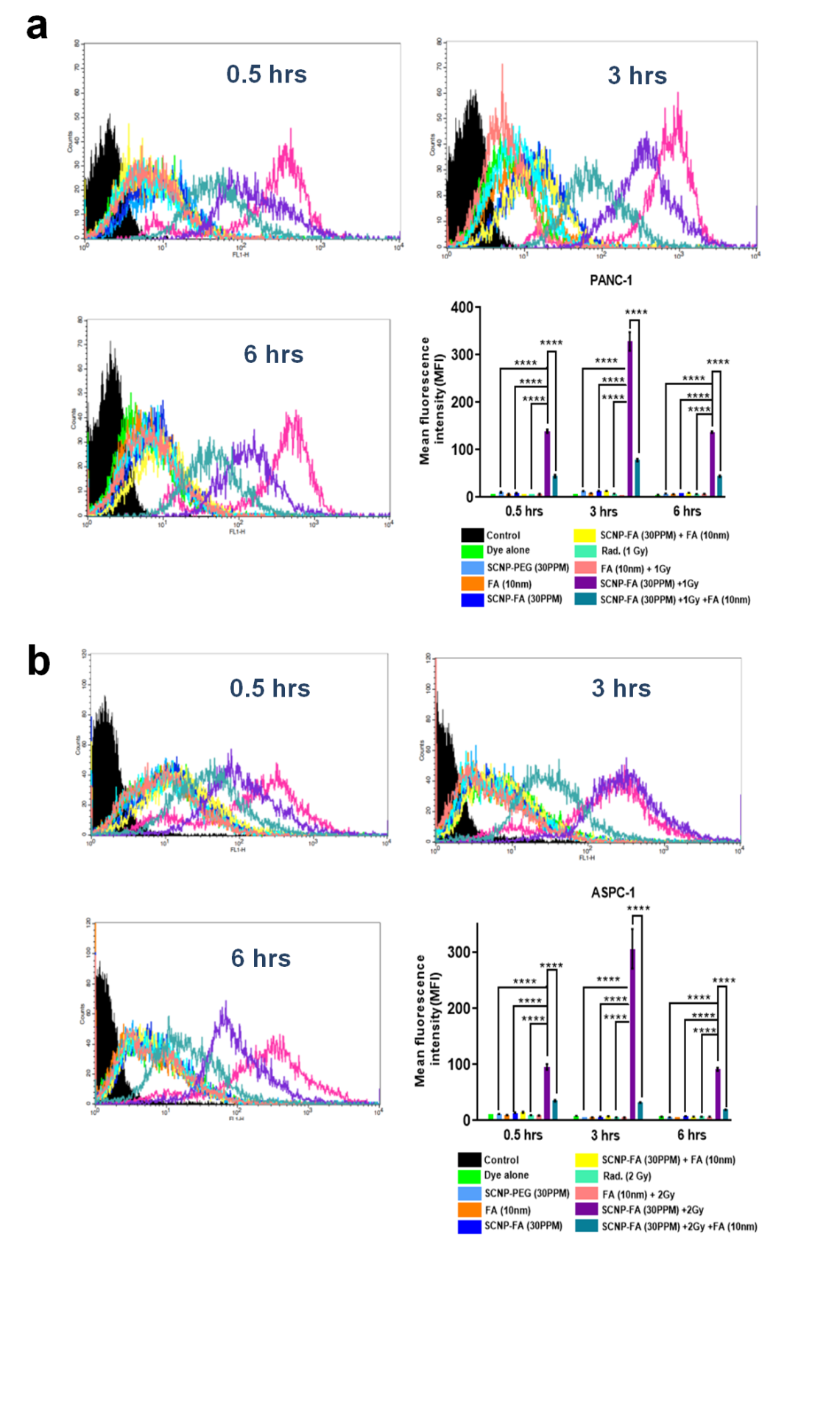


**Supplementary Figure 9. Intracellular ROS production via ferroptosis.** (a) Time-dependent ROS detection by H2DCFDA in PANC-1 after different treatments with their respective histogram peak depending on their fluorescence intensity.The black curve represents the untreated control group, green curve represents the H2DCFDA dye alone group, light blue curve represents the SCNP-PEG alone treated group, orange curve represents the FA alone treated group, dark blue curve represents the SCNP-FA alone treated group, yellow curve represents the SCNP-FA with excess FA treated group, turquoise curve represents radiation alone treated group, pink curve represents FA with radiation treated group, purple curve represents SCNP-FA with radiation treated group, dark green curve represents SCNP-FA with excess FA and radiation treated group and dark pink curve represents the positive H_2_O_2_ control treated group. The significant rightward shift in fluorescence intensity indicative of elevated ROS generation.(b) Time-dependent ROS detection by H2DCFDA in ASPC-1 after different treatments detected by flow cytometric analysis with their respective histogram peak depending on their fluorescence intensity (n=3). Data are presented as mean± SD (****P< 0.0001).


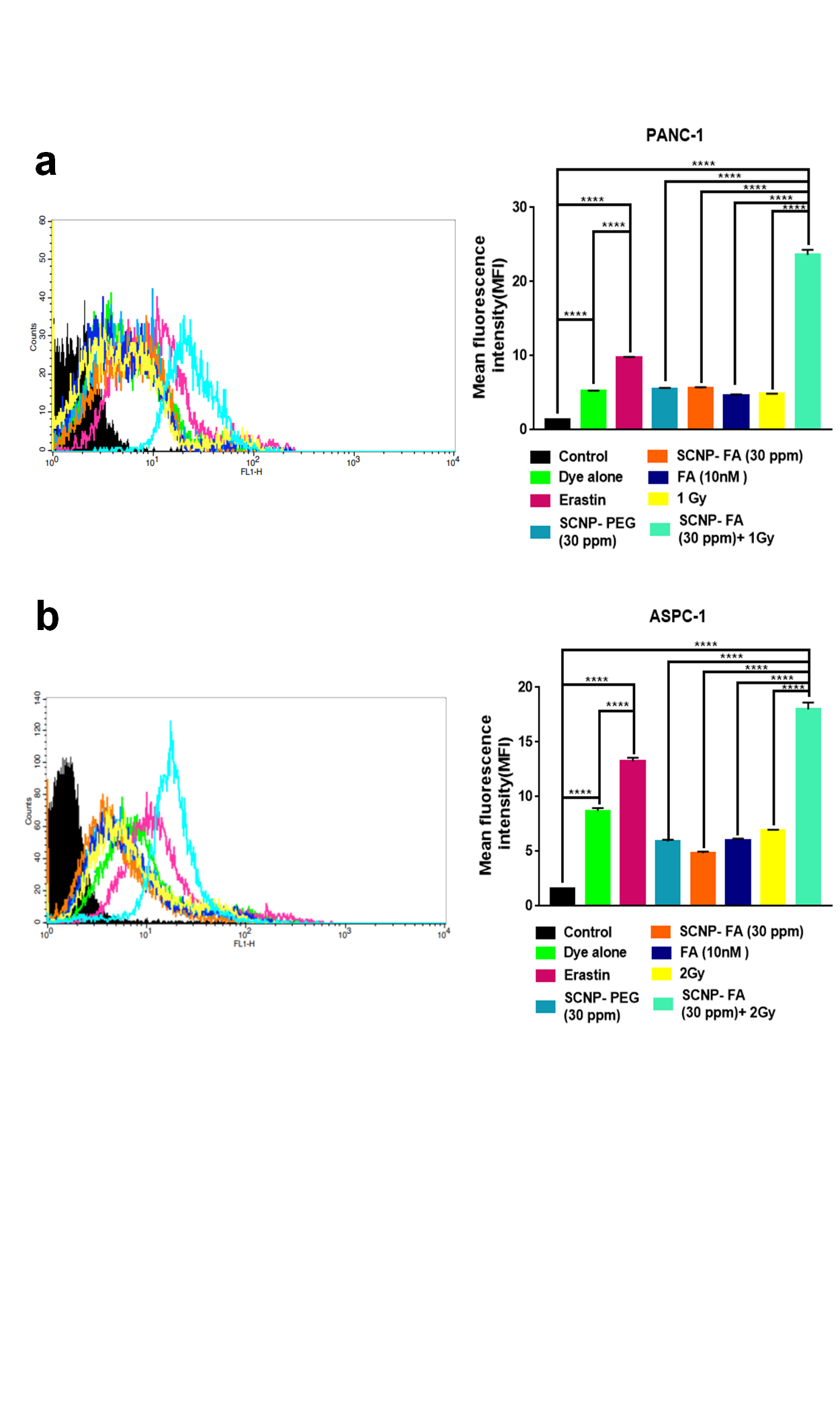


**Supplementary Figure 10. Lipid ROS generation via ferroptosis.** (a) ROS detected by flow cytometric analysis in PANC-1 after various treatments with their respective histogram peak depending on their fluorescence intensity. The black curve represents the untreated control group, green curve represents the C11-BODIDY dye alone group, pink curve represents the positive erastin control treated group, light blue curve represents the SCNP-PEG alone treated group, orange curve represents the SCNP-FA alone treated group, dark blue curve represents the FA alone treated group, yellow represents the radiation alone treated group and turquoise curve represents the SCNP-FA with radiation treated group. The significant rightward shift in fluorescence intensity indicative of elevated lipid ROS generation.(b) ROS detected by flow cytometric analysis in ASPC-1 after various treatments with their respective histogram peak depending on their fluorescence intensity (n=3). Data are presented as mean± SD (****P< 0.0001).


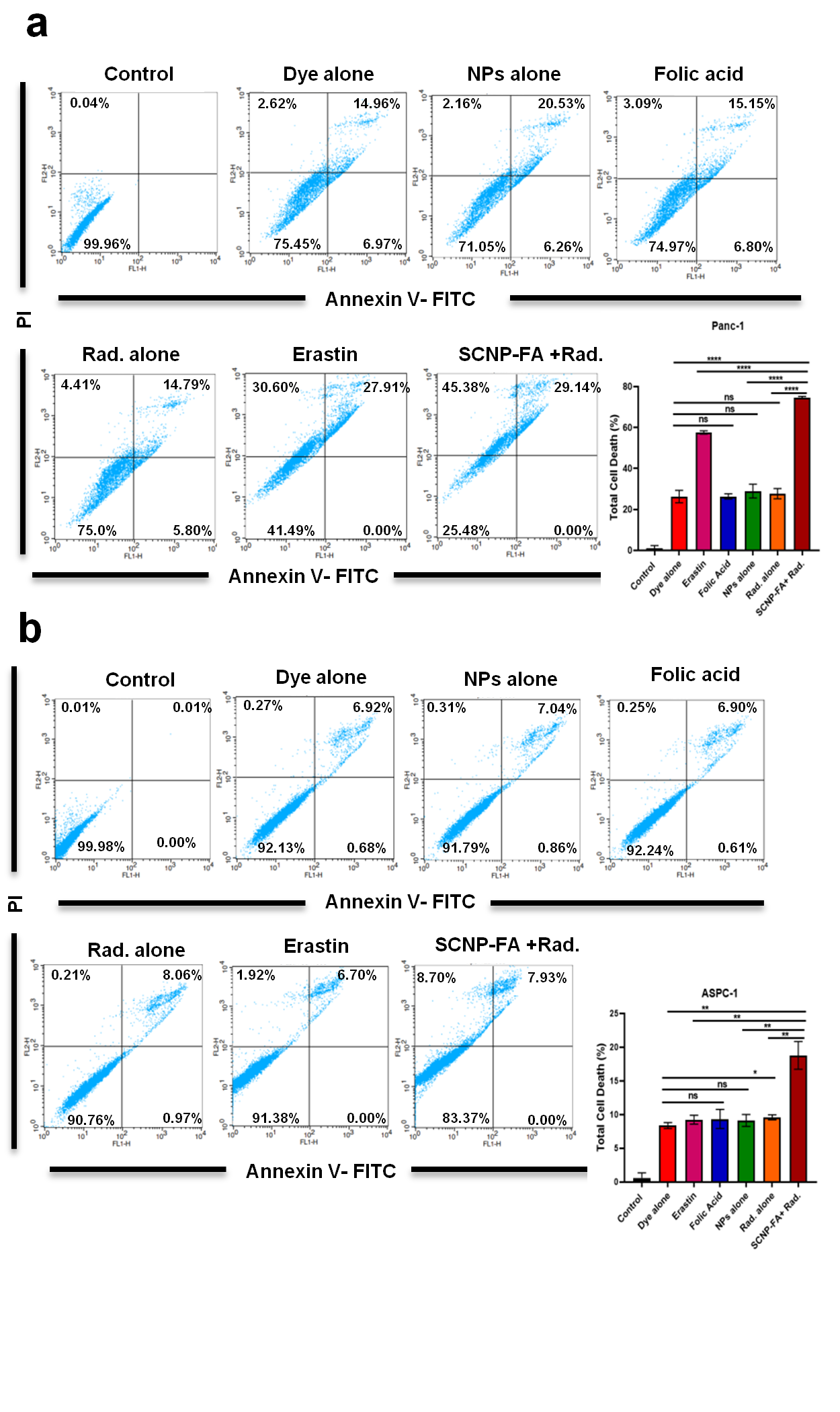


**Supplementary Figure 11. Determining cell death via ferroptosis.** (a) Flow cytometric analysis by Annexin V-FITC/PI staining in PANC-1. (b) Flow cytometric analysis by Annexin V-FITC/PI staining in ASPC-1 upon various treatments with erastin as a positive control (n=3). Data are presented as mean± SD (*P <0.05, **P< 0.01, *** P<0.001).


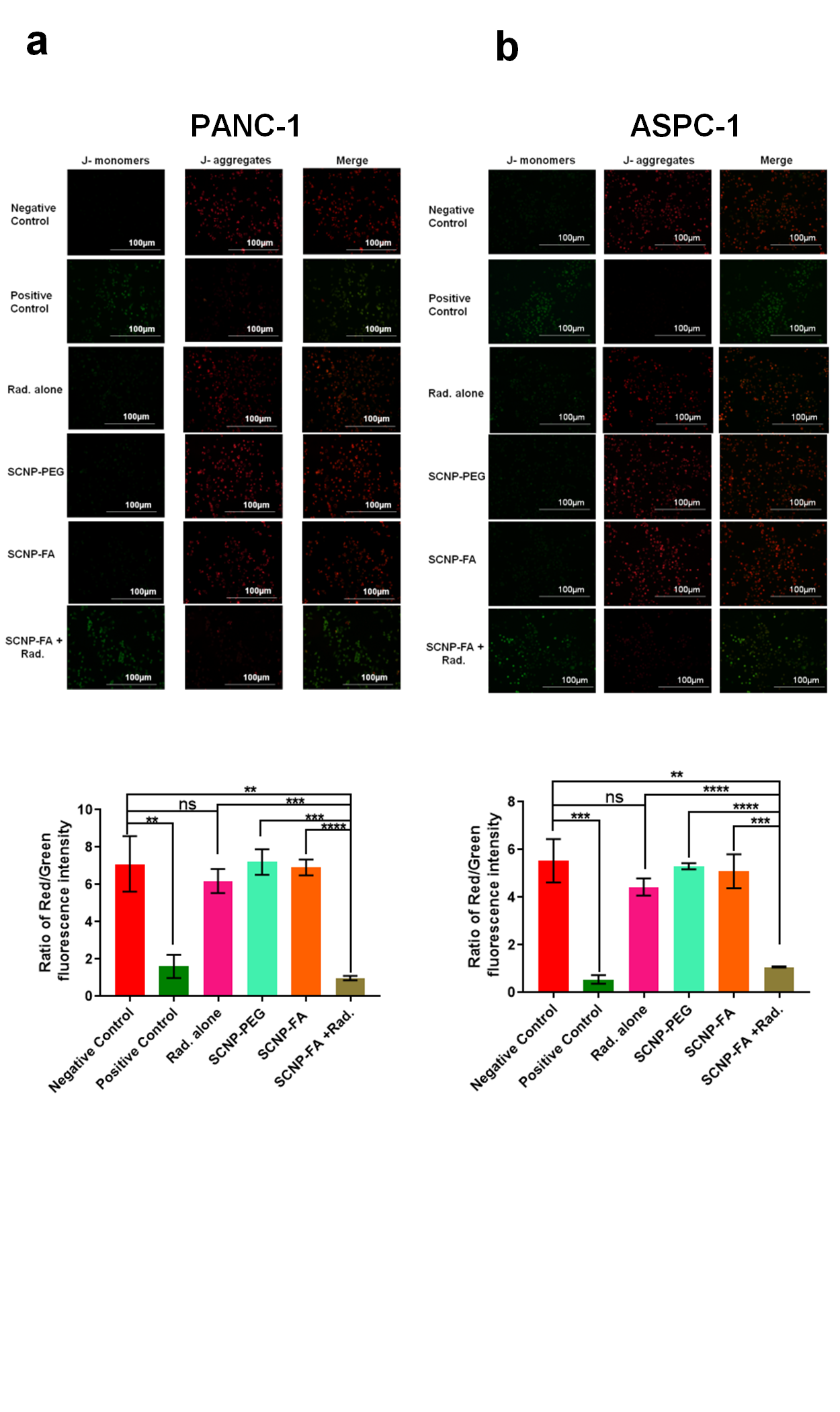


**Supplementary Figure 12. Determining mitochondrial membrane potential (MMP,** **ΔΨm).** (a) MMP in PANC-1 by JC-1 assay upon treatment with different groups. (b) MMP in ASPC-1 by JC-1 assay upon treatment with different groups. The scale bar stands for 100 μm. Data are presented as mean± SD (**P< 0.01 *** P<0.001, ****P< 0.0001).


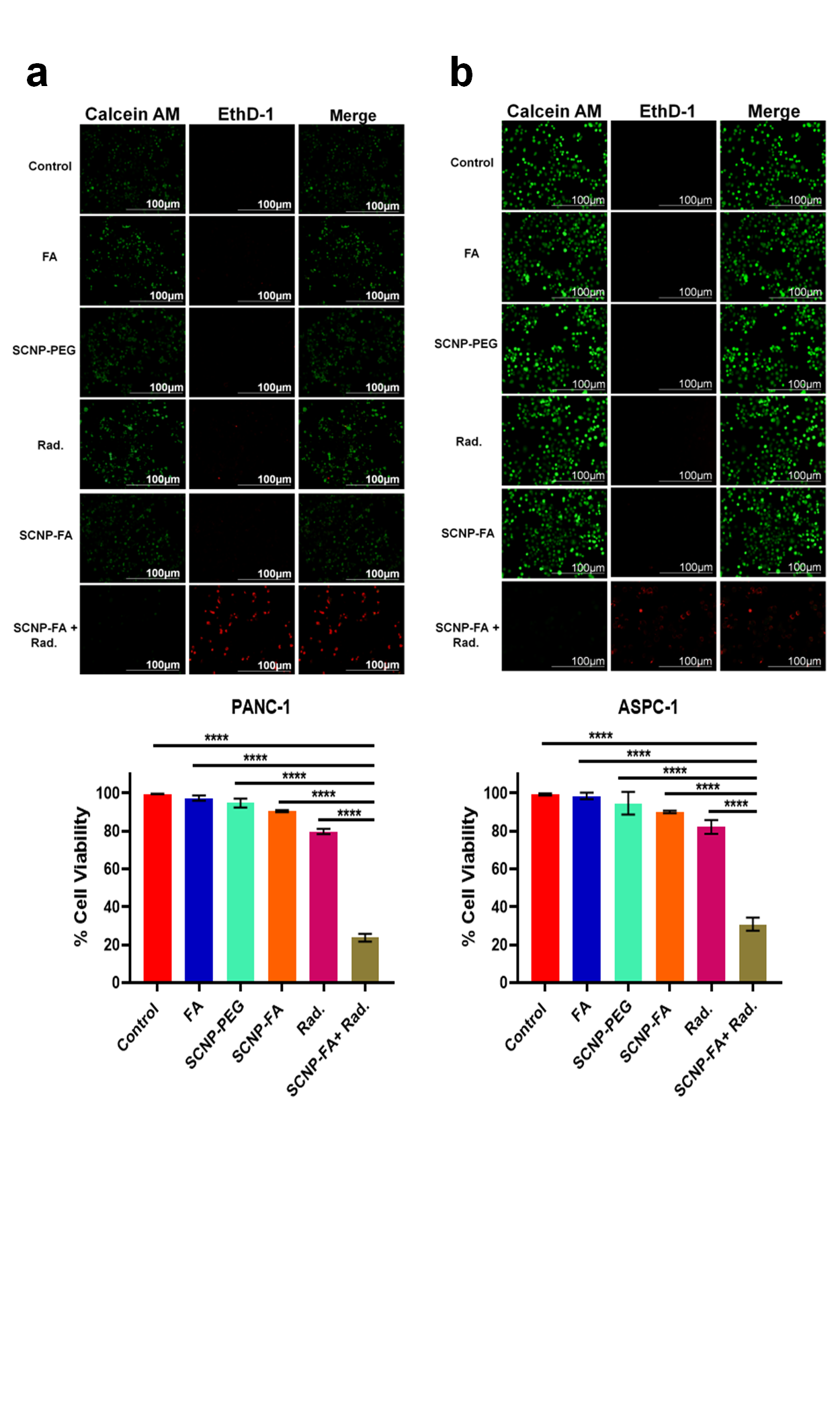


**Supplementary Figure 13. Determining cell death by live/dead staining.** (a) PANC-1 upon various treatments. (b) ASPC-1 upon various treatments. The scale bar stands for 100 μm. Data are presented as mean± SD (****P< 0.0001).


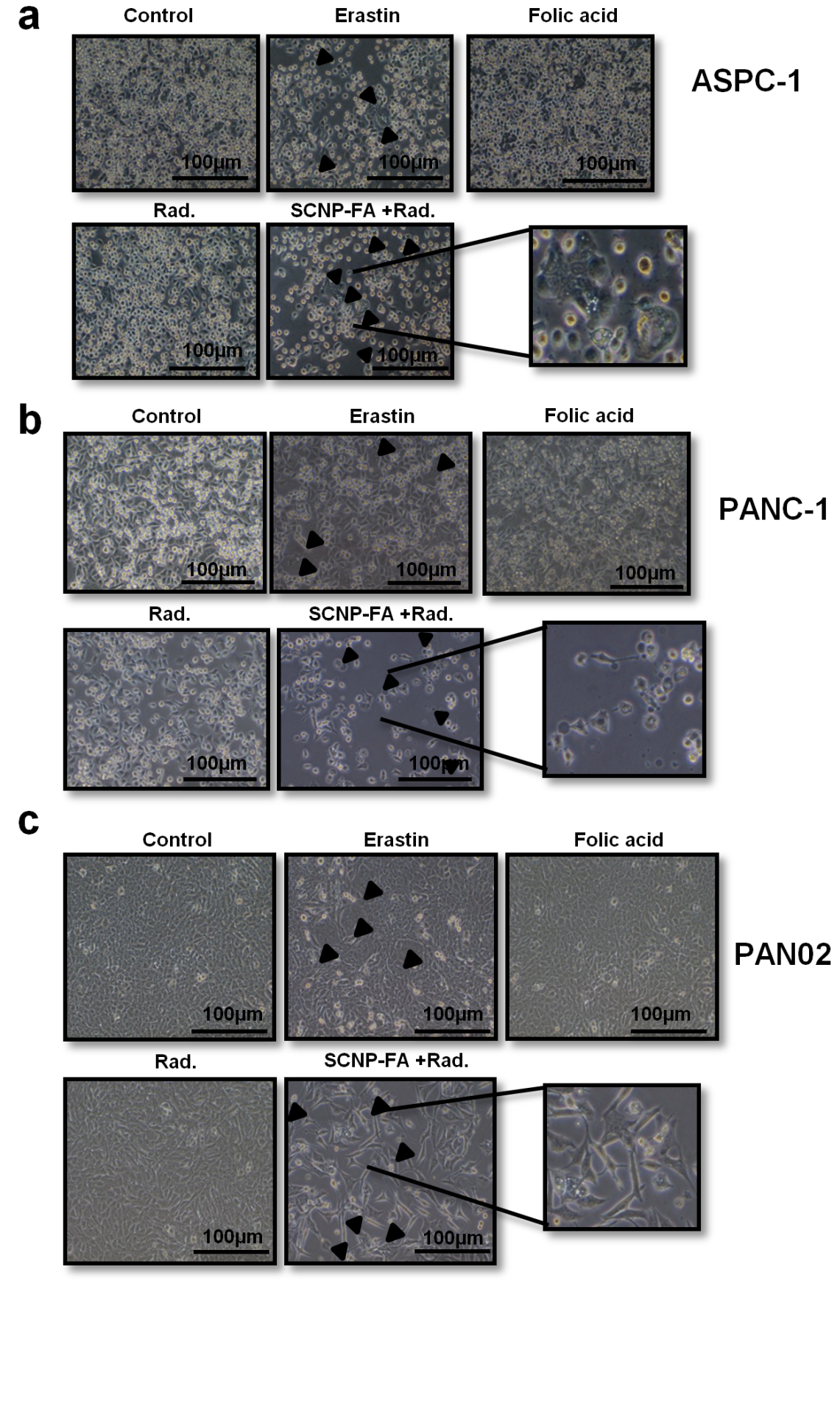


**Supplementary Figure 14. Morphological changes in cells induce ferroptosis.** (a) ASPC-1 (b) PANC-1 (c) PAN02 bright field images and magnification illustrate the ballooning phenotype in the cells. The scale bar stands for 100 μm.


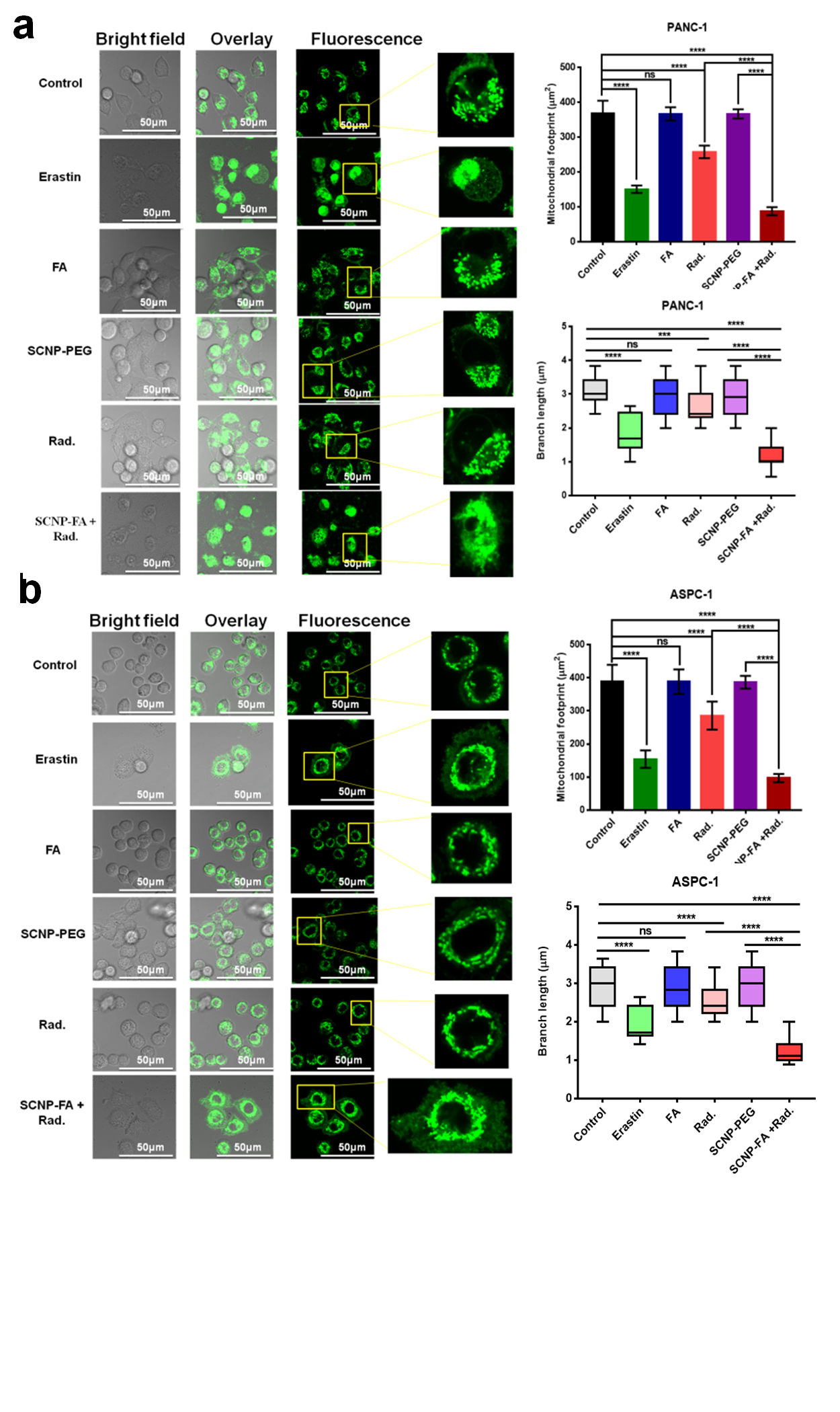


**Supplementary Figure 15. Mitochondrial morphological changes upon treatment.** (a) PANC-1 (b) ASPC-1 fluorescence and magnified images illustrating the fragmented and distorted mitochondria with erastin as a positive control. The scale bar stands for 50 μm. Data are presented as mean± SD (*** P<0.001, ****P< 0.0001).

 
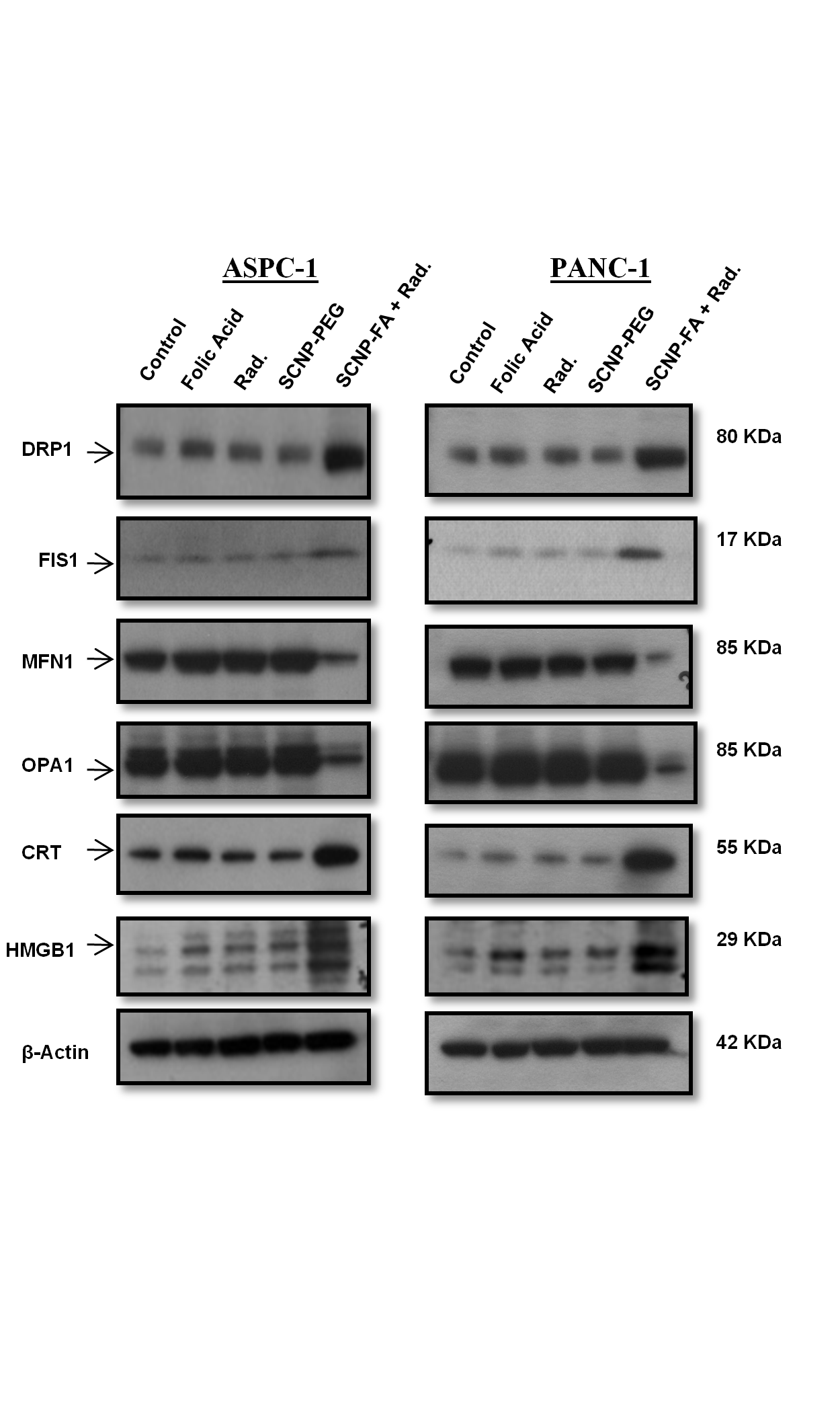


**Supplementary Figure 16. Mitochondrial dynamics induces ICD.** Protein expression levels for fission, fusion and ICD markers analyzed upon various treatments (n=3).


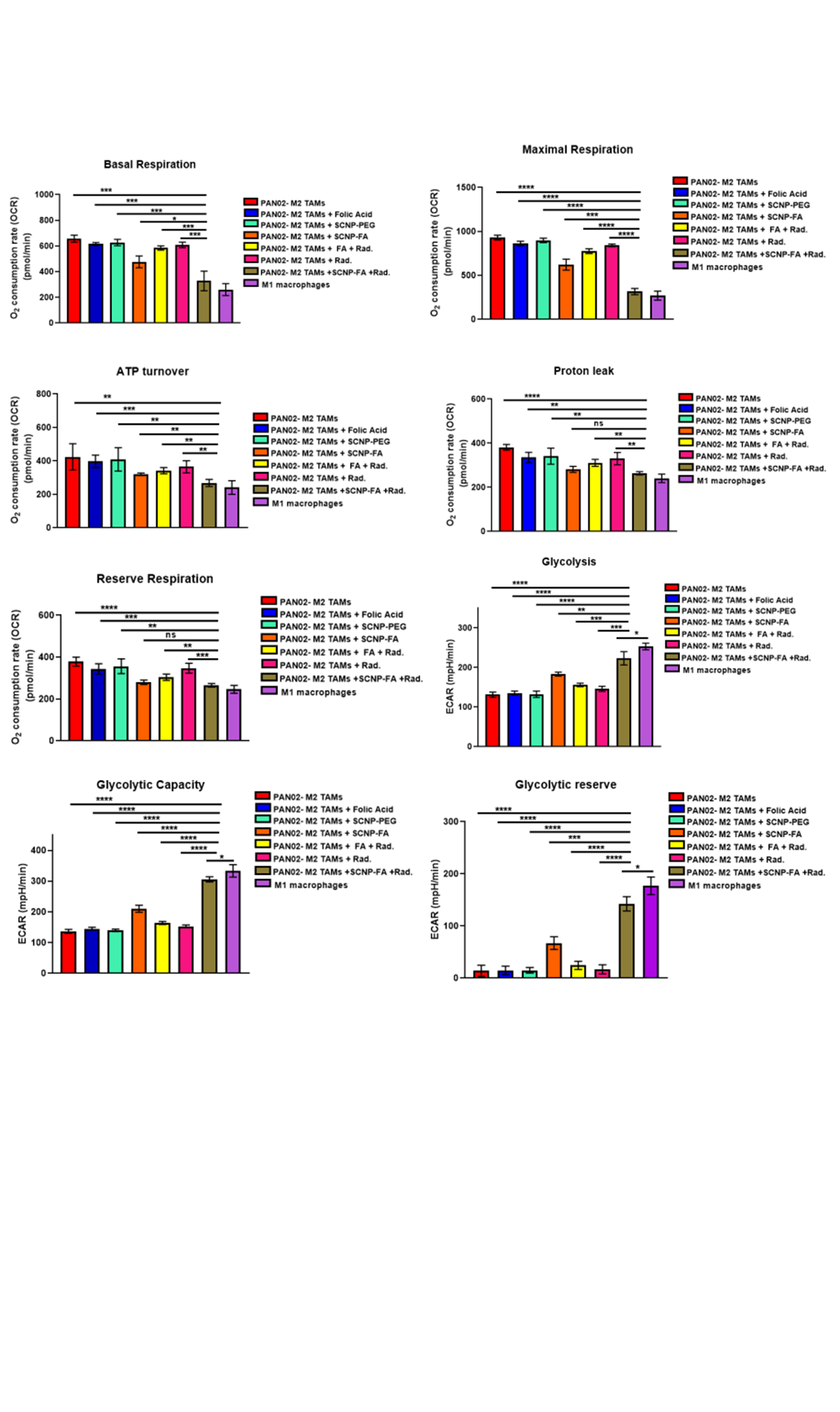


**Supplementary Figure 17. Statistical Analysis of Seahorse OCR and ECAR after treatment in tumor-associated macrophages.** Basal respiration, maximal respiration, ATP turnover, reserve respiration, proton leak, glycolysis, glycolytic capacity, and glycolytic reserve were measured. Data are presented as mean ± SD (*P <0.05, **P< 0.01 *** P<0.001, ****P< 0.0001).


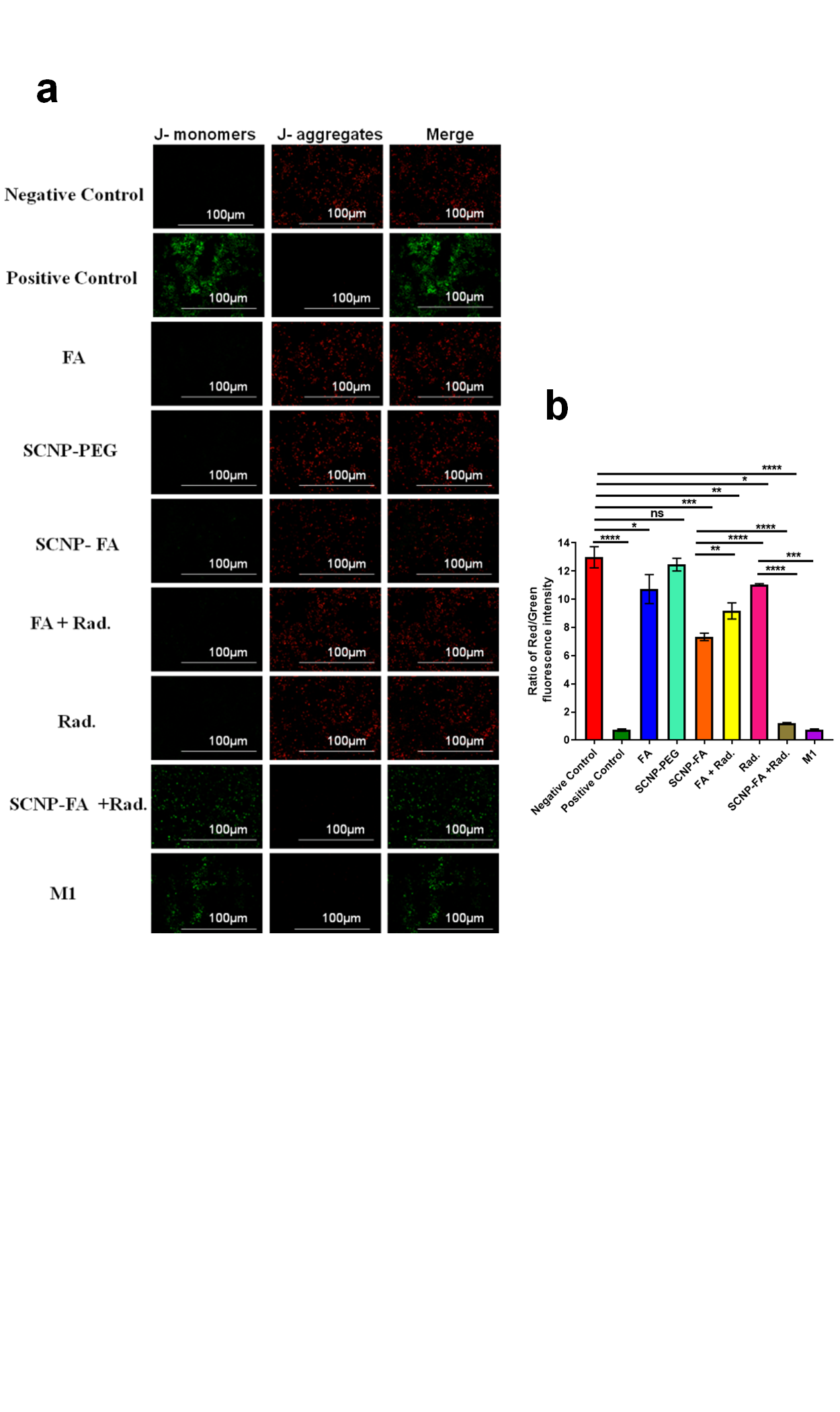


**Supplementary Figure 18. Determining mitochondrial membrane potential (MMP,** **ΔΨm).** (a) MMP in PAN02 TAMs by JC-1 assay upon treatment with different groups. (b) Statistical analysis determining fluorescence intensity upon treatment with different groups. The scale bar stands for 100 μm. Data are presented as mean± SD (*P <0.05, **P< 0.01, *** P<0.001, ****P< 0.0001).


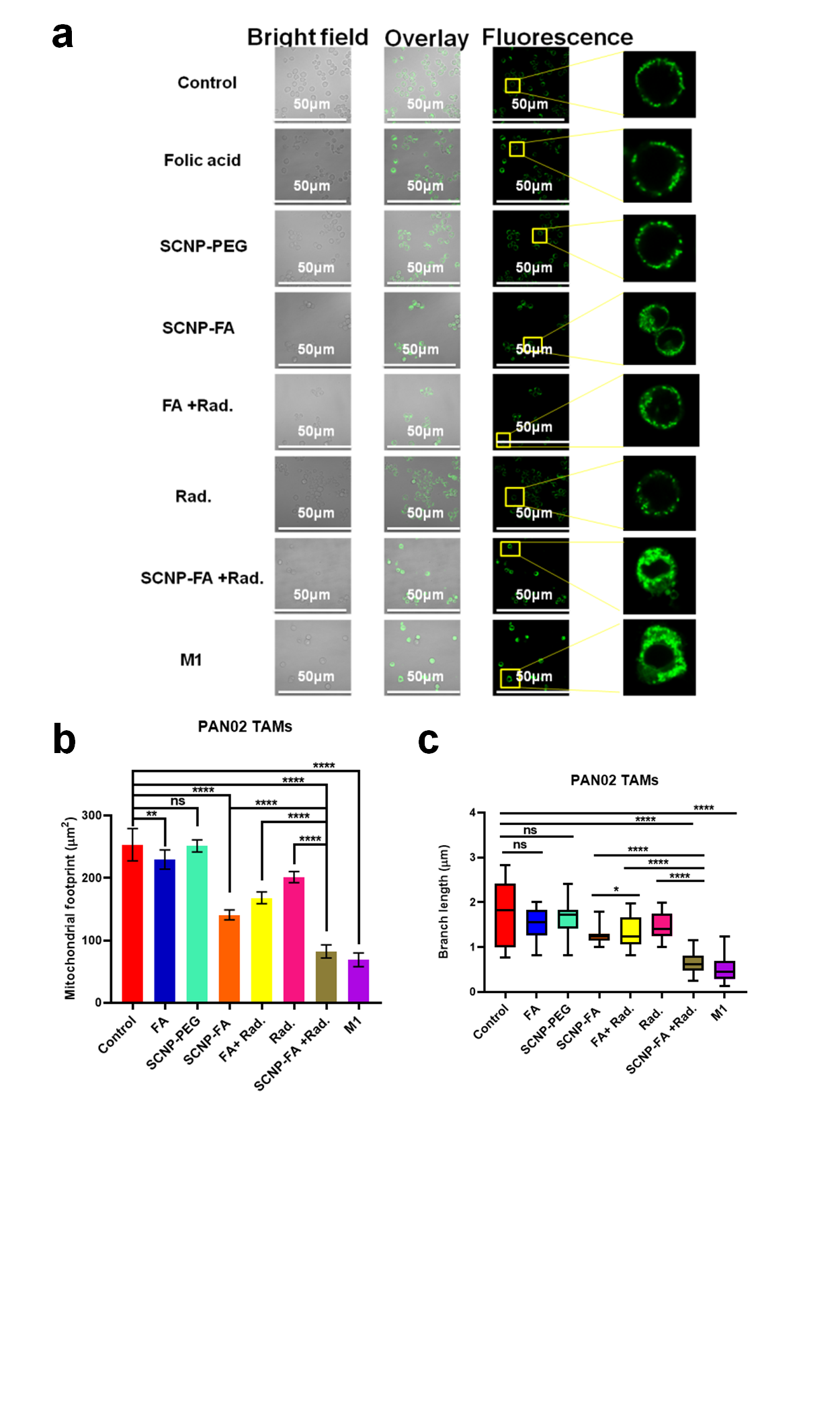


**Supplementary Figure 19. Mitochondrial morphological changes upon treatment in PAN02 TAMs.** (a) fluorescence and magnified images illustrating the fragmented and distorted mitochondria with M1 as positive control (b) mitochondrial footprint (c) branch length. The scale bar stands for 50 μm. Data are presented as mean± SD (**P< 0.01, *** P<0.001, ****P< 0.0001).


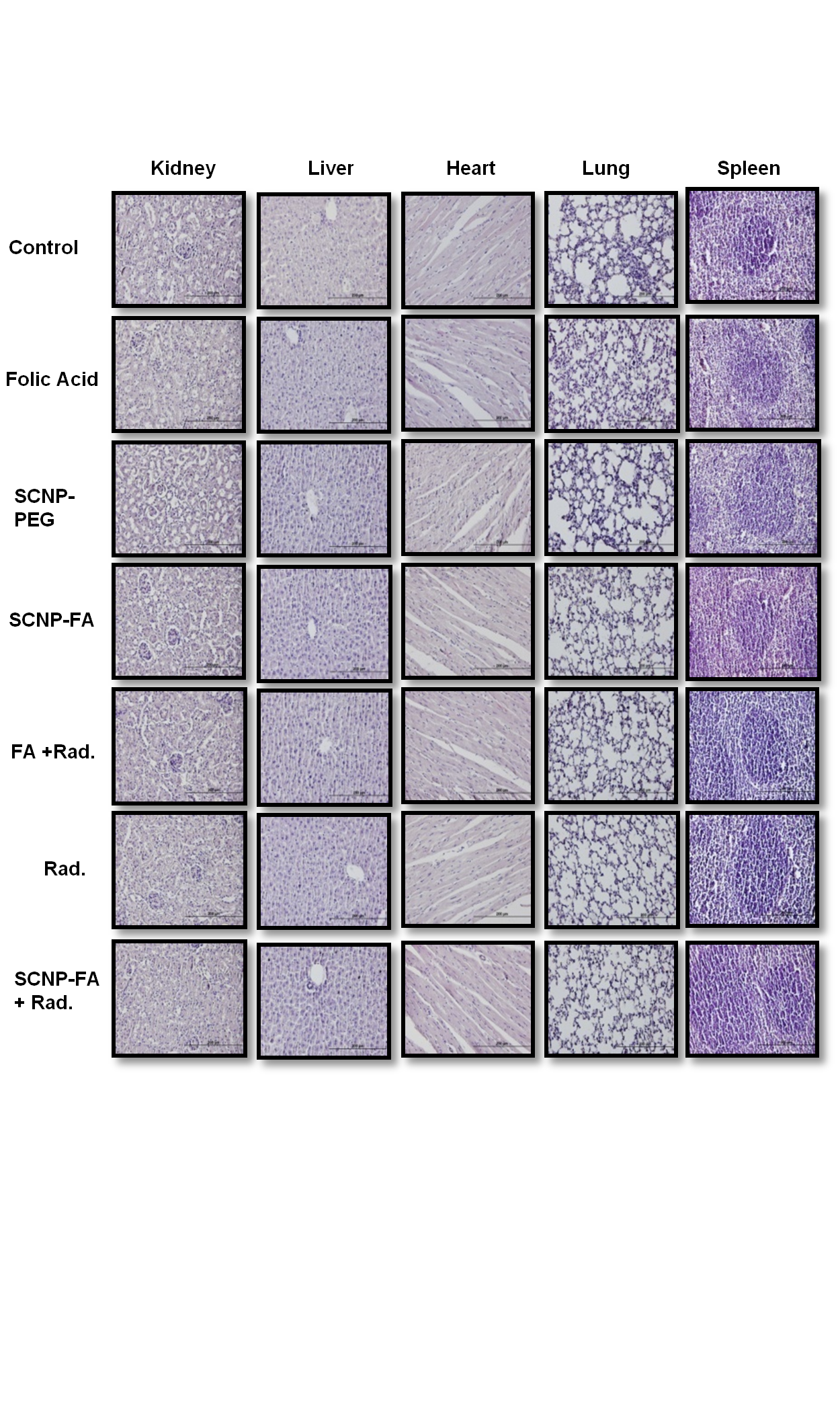


**Supplementary Figure 20. Biosafety confirms no apparent inflammation.** H/E staining of the organs from the mice treated with various groups. The scale bar stands for 200 μm.

.


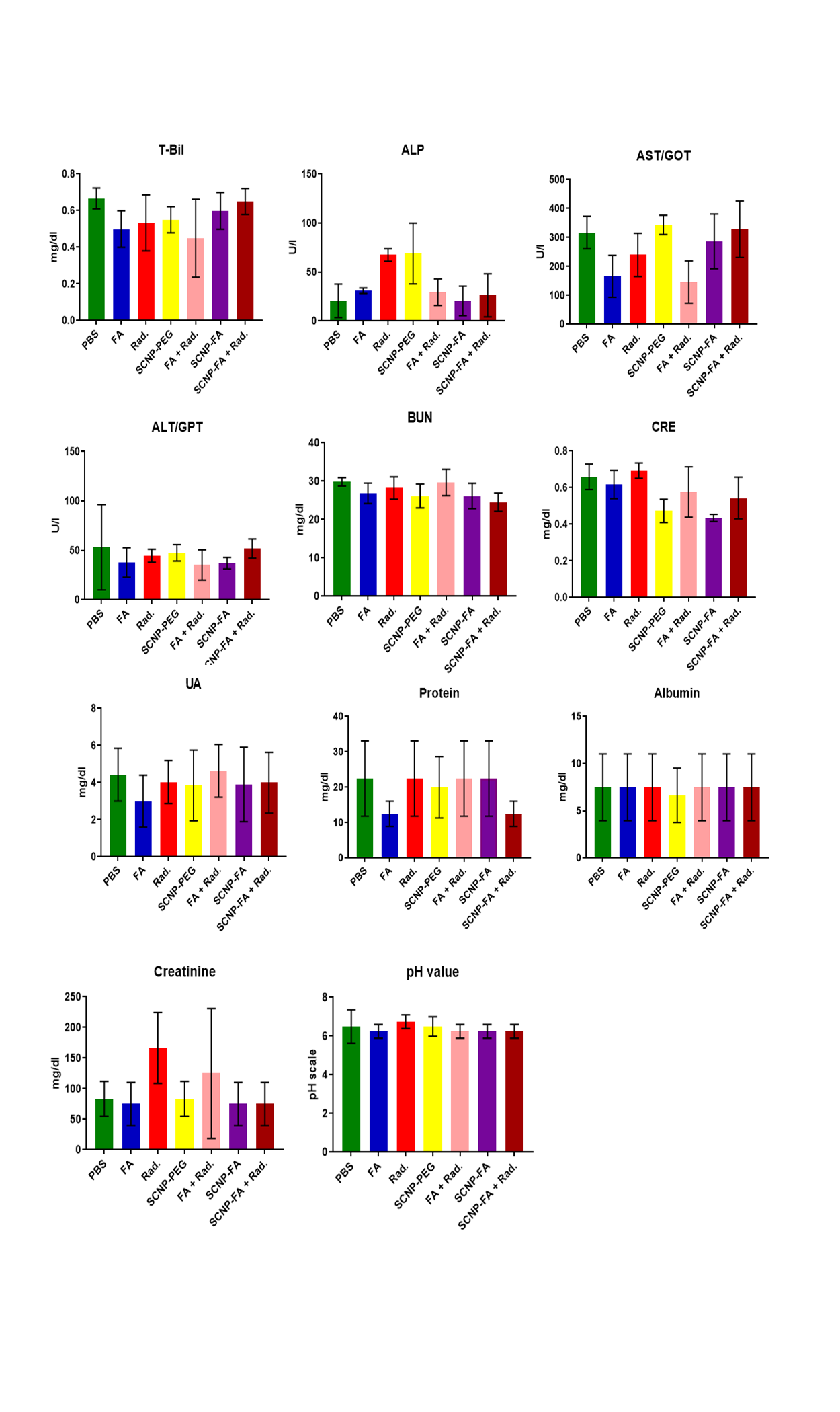


**Supplementary Figure 21. Biochemical analysis confirms no apparent toxicity.** Serum and urine analysis from the mice treated with various groups. Other parameters in urine analysis, such as glucose, bilirubin, blood, ketone bodies, nitrite, and leukocyte, were declared NIL.


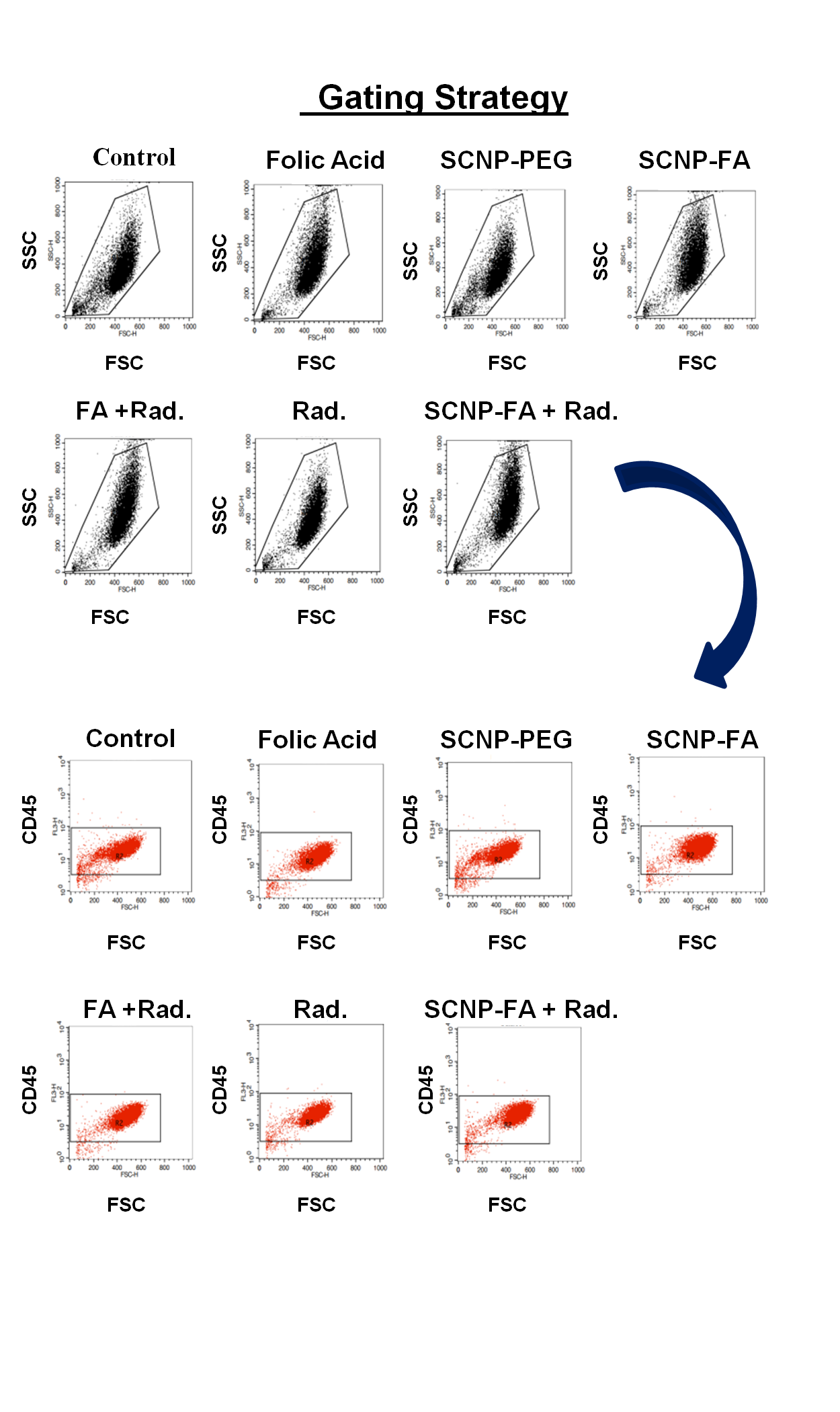


**Supplementary Figure 22. Gating Strategy by flow cytometry.** Gating of cell population in various treatment groups.


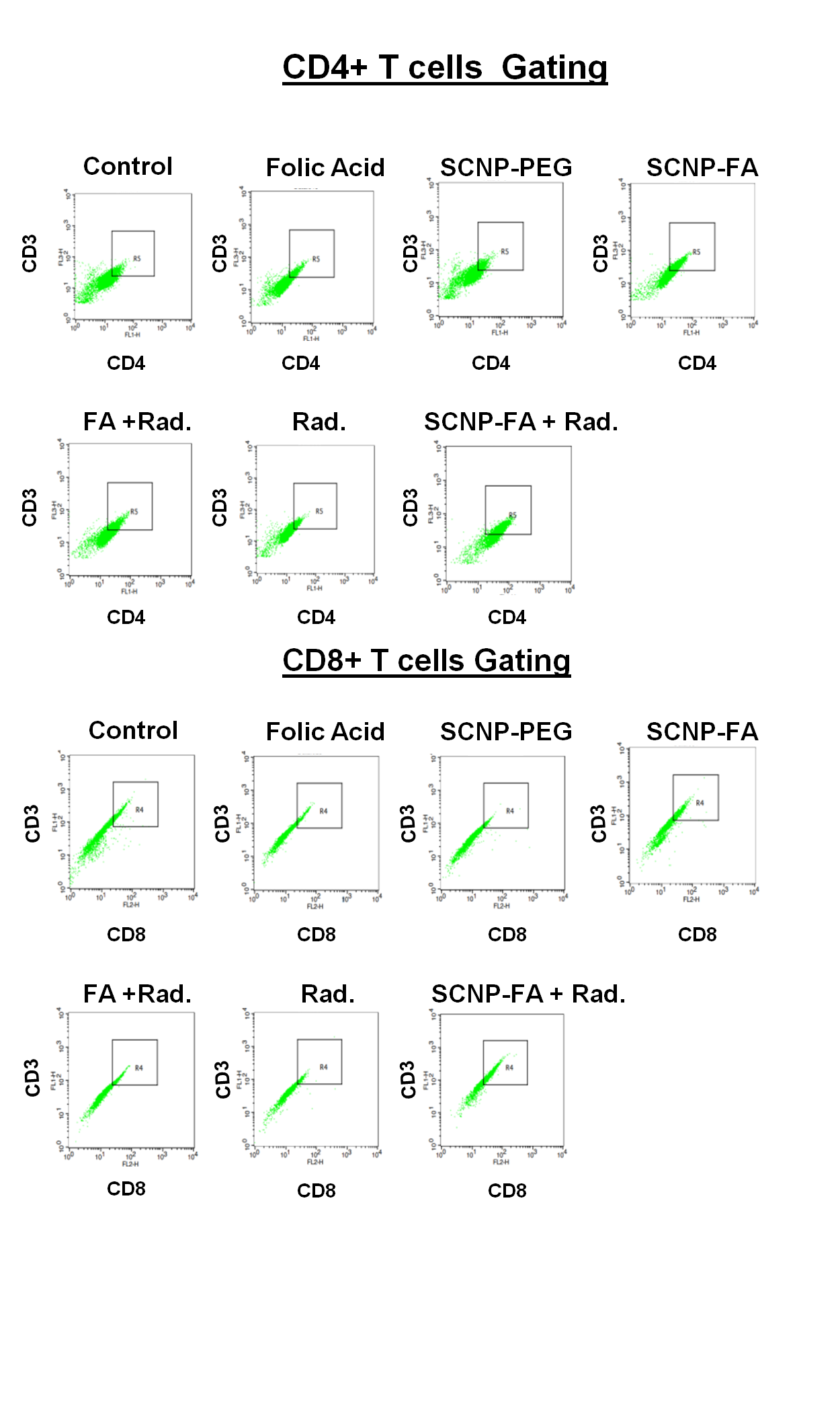


**Supplementary Figure 23. Gating of CD4+ and CD8+ T cells.**


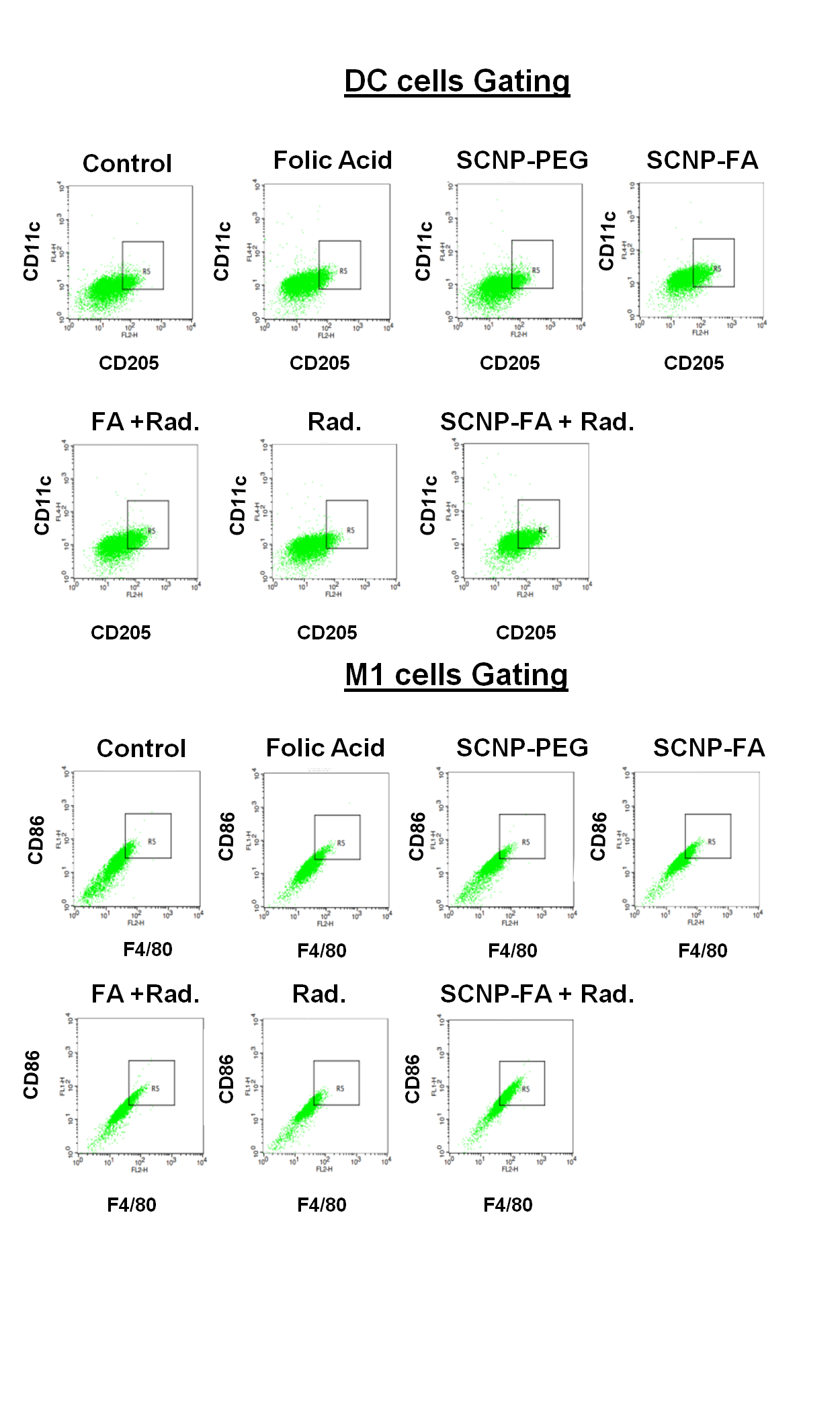


**Supplementary Figure 24. Gating of DCs and M1 TAMs cells.**


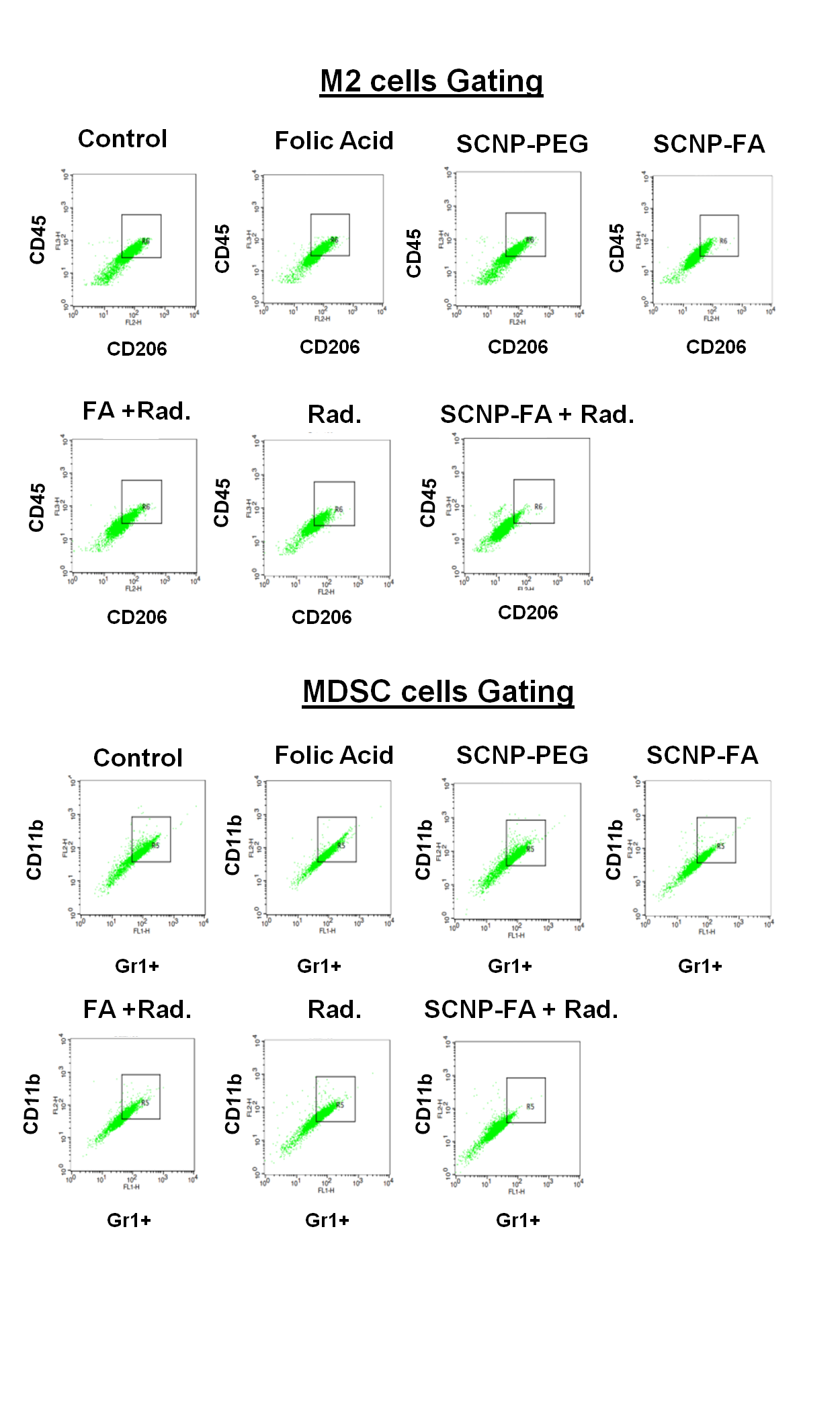


**Supplementary Figure 25. Gating of M2 TAMs and MDSCs cells.**


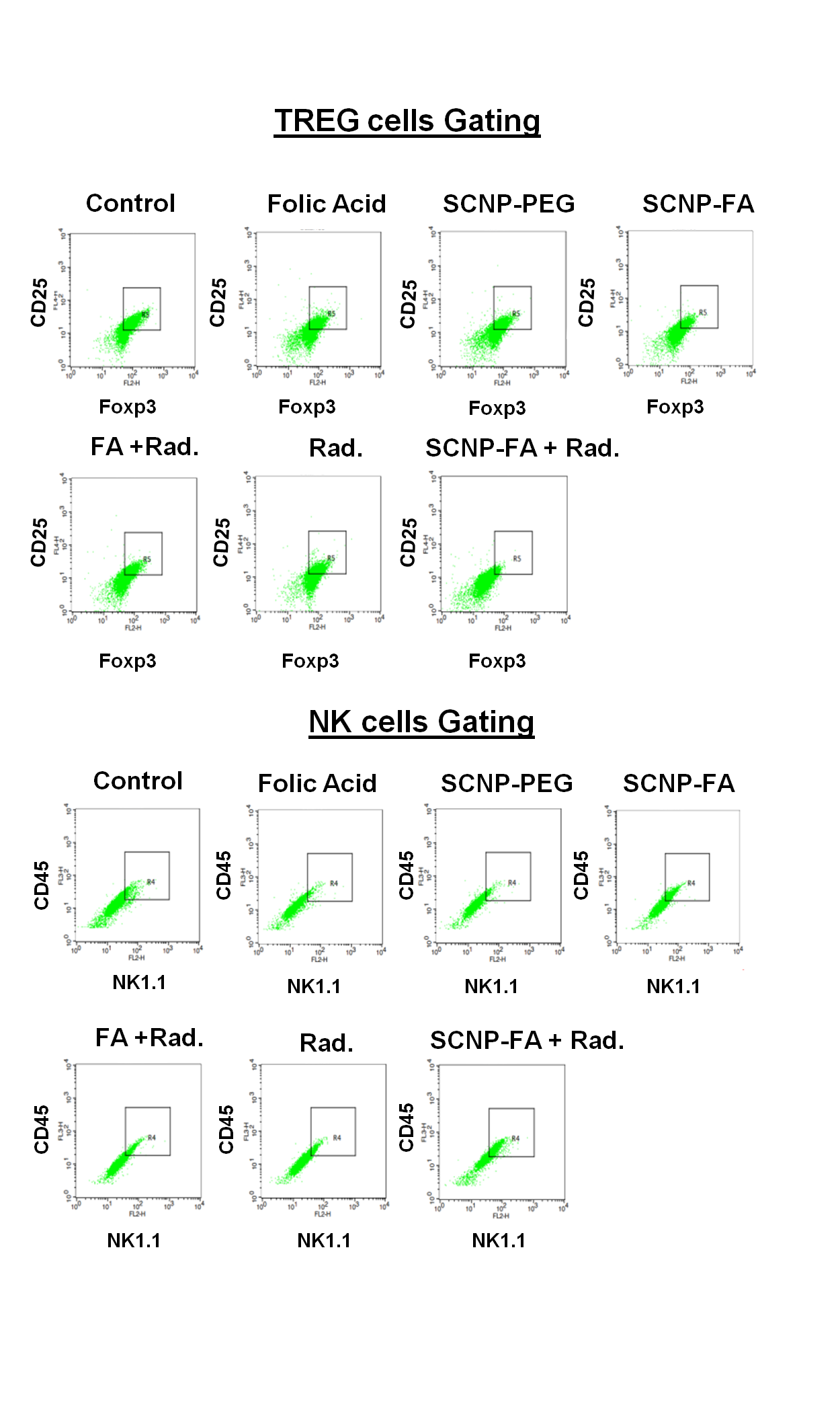


**Supplementary Figure 26. Gating of Tregs and NK cells.**


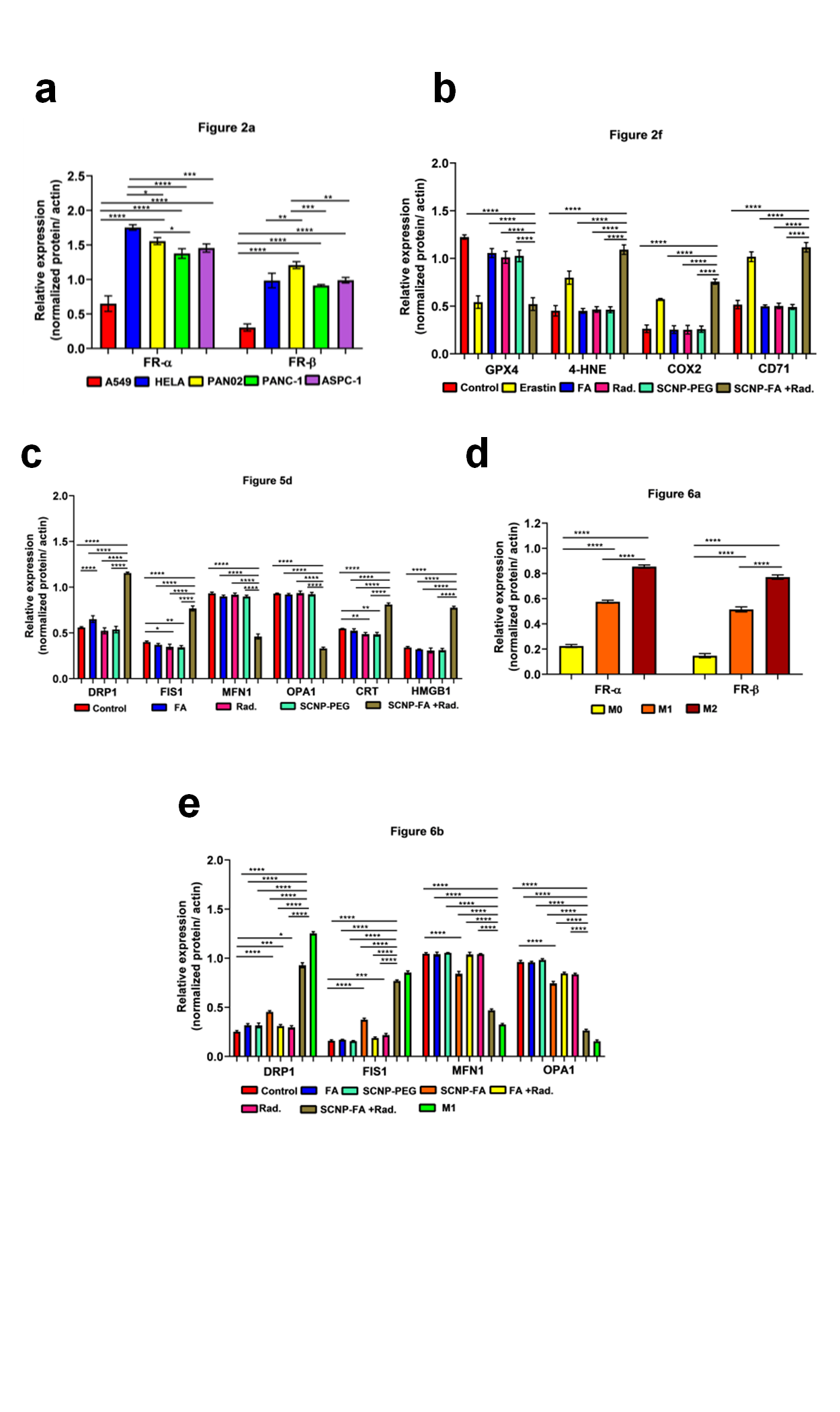


**Supplementary Figure 27. Quantification of Western Blots** (a) Relative expression level of targeting protein compared to actin from Fig. 2a, (b) Fig. 2f, (c) Fig. 5d, (d) Fig. 6a, and (e) Fig. 6b. Statistical significance was determined by ordinary two-way Anova. (*P <0.05, **P< 0.01, *** P<0.001, ****P< 0.0001).

**
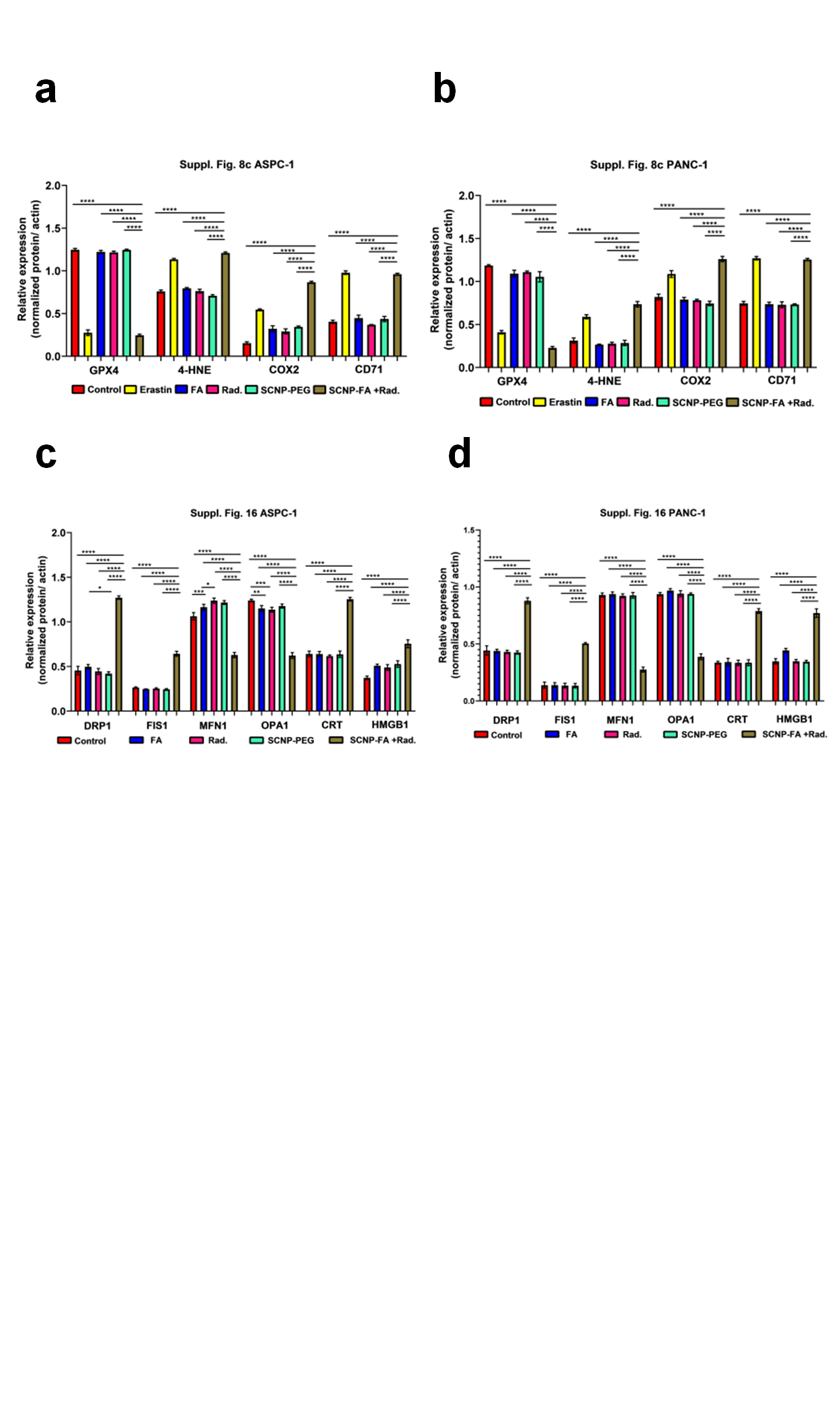
**

**Supplementary Figure 28. Quantification of Western Blots** (a) Relative expression level of targeting protein compared to actin from Suppl. Fig. 8c ASPC-1, (b) Suppl. Fig. 8c PANC-1, (c) Suppl. Fig. 16 ASPC-1, and (d) Suppl. Fig. 16 PANC-1. Statistical significance was determined by ordinary two-way Anova. (*P <0.05, **P< 0.01, *** P<0.001, ****P< 0.0001).

**
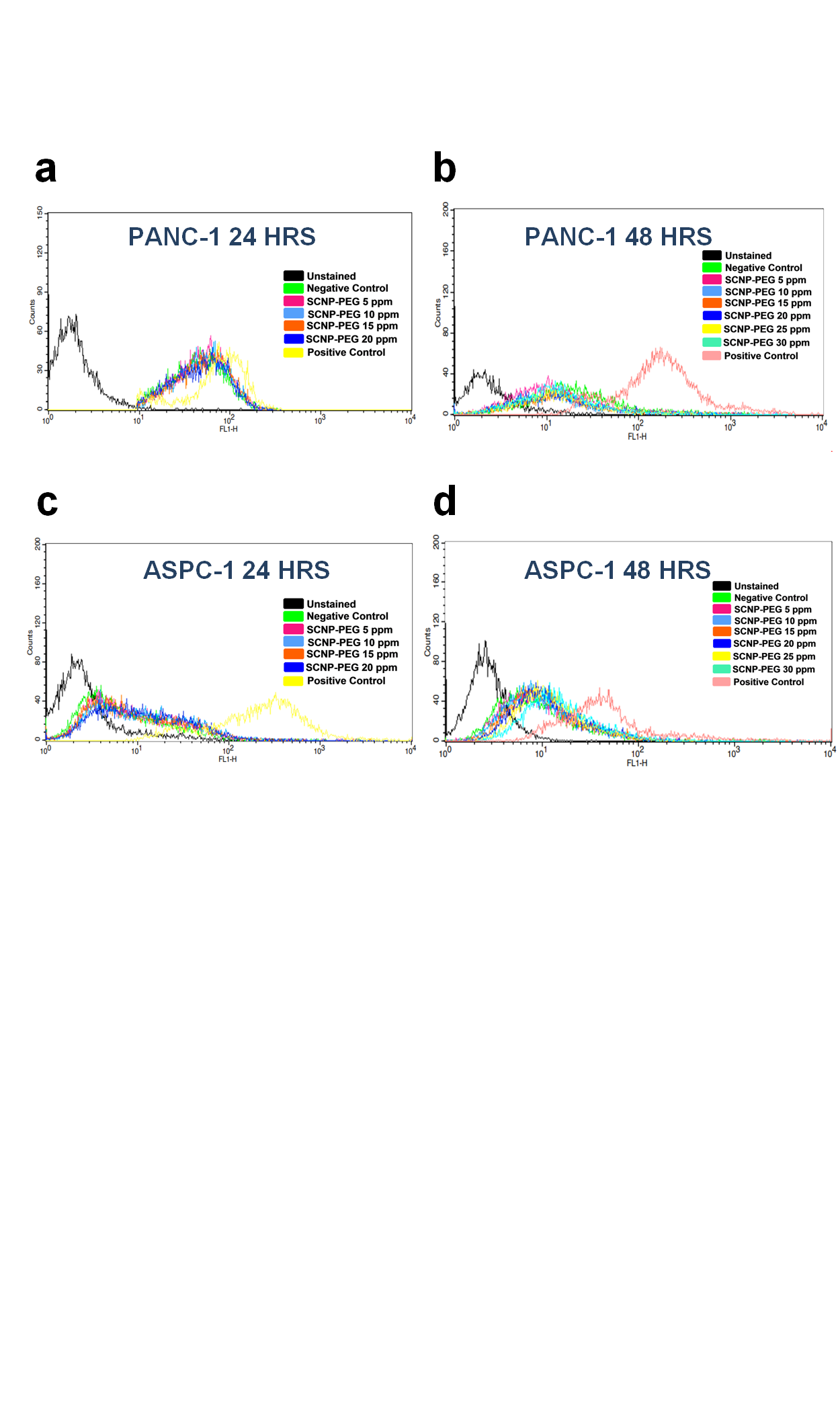
**

**Supplementary Figure 29. Intracellular ROS production with SCNP-PEG shows no significant changes.** (a) Dose-dependent ROS detection by H2DCFDA in PANC-1 after 24 hrs upon SCNP-PEG and radiation treatment with their respective histogram peak depending on their fluorescence intensity. (b) Dose-dependent ROS detection by H2DCFDA in PANC-1 after 48 hrs upon SCNP-PEG and radiation treatment with their respective histogram peak depending on their fluorescence intensity. (c) Dose-dependent ROS detection by H2DCFDA in ASPC-1 after 24 hrs upon SCNP-PEG and radiation treatment with their respective histogram peak depending on their fluorescence intensity. (d) ) Dose-dependent ROS detection by H2DCFDA in ASPC-1 after 48 hrs upon SCNP-PEG and radiation treatment with their respective histogram peak depending on their fluorescence intensity.
